# One-Step PCR Detection of *Salmonella* Pullorum/Gallinarum Using a Novel Target: The Flagellar Biosynthesis Gene *flhB*

**DOI:** 10.3389/fmicb.2016.01863

**Published:** 2016-11-22

**Authors:** Dan Xiong, Li Song, Shizhong Geng, Jing Tao, Shumin An, Zhiming Pan, Xinan Jiao

**Affiliations:** ^1^Jiangsu Key Laboratory of Zoonosis, Yangzhou UniversityYangzhou, China; ^2^Jiangsu Co-innovation Center for Prevention and Control of Important Animal Infectious Diseases and ZoonosesYangzhou, China; ^3^Joint International Research Laboratory of Agriculture and Agri-product Safety of the Ministry of EducationYangzhou, China

**Keywords:** *Salmonella* Pullorum/Gallinarum, *flhB*, PCR detection, chicken farm, one-step

## Abstract

*Salmonella enterica* serovar Pullorum/Gallinarum is an important infectious pathogen that has caused widespread problems for chicken industry. Traditional *Salmonella* serotyping is an expensive and time-consuming process. In this study, we developed a rapid one-step polymerase chain reaction (PCR) method to identify *S.* Pullorum/Gallinarum. The PCR-based assay focuses on *flhB*, which shows a deficient region only in *S.* Pullorum/Gallinarum, compared with that of other serovars. The specificity and sensitivity of the PCR system were evaluated. The developed PCR method could identify *S.* Pullorum/Gallinarum from 27 different *Salmonella* serovars and eight non-*Salmonella* pathogens. The minimum limit of DNA and the lowest number of cells of *S.* Pullorum for the PCR detection were no less than 5.85 pg/μL and 10 CFU, respectively. The method was applied to the analysis of *Salmonella* strains isolated from the chicken farm. The PCR-based testing results of the farm isolates were in concordance with those obtained using traditional serotyping method. This newly developed PCR-based system could be used to accurately screen for the presence of *S.* Pullorum/Gallinarum, and support traditional serotyping methods, especially in high-throughput screening situations.

## Introduction

Although there are more than 2,600 *Salmonella* serovars (Ranieri et al., 2013), most animal infections are caused by relatively few serovars (Nielsen, 2013; Saeki et al., 2013; Zhu et al., 2015). Fowl are the specific host of *S.* Gallinarum biovars Pullorum and Gallinarum, which cause “white diarrhea” (pullorum disease) and fowl typhoid, respectively (Soria et al., 2012). *S.* Gallinarum can spread to reproductive organs, resulting in vertical transmission of the pathogen, as well as egg-related salmonellosis (Keller et al., 1997). Thus, timely detection of *S.* Pullorum/Gallinarum is very important. Because food animals and poultry are important reservoirs of *Salmonella* (Henson, 1997; Lynch et al., 2006), the United States Department of Agriculture and Food Safety Inspection Service carry out an “in plant” Hazard Analysis and Critical Control Point program to reduce the prevalence of food-borne pathogen contamination in meats, eggs, and milk (Hong et al., 2008).

Traditional *Salmonella* serotyping is conducted according to the White–Kauffmann–Le Minor scheme, which identifies the somatic (O) and flagellar (H) antigens based on the agglutination of bacteria with specific antisera (Majchrzak et al., 2014). Serotyping allows comparison with historical data, because it has been used for almost 70 years. Identifying the causative *S. enterica* serovars is a necessary first step in any epidemiological investigation of food-borne outbreaks. Despite its widespread use, traditional serotyping has a number of drawbacks. It takes at least 3 days to complete, is labor-intensive and expensive, requires the maintenance of 250 typing antisera and 350 different antigens, and is unable to differentiate between rough or mucoid strains (Ranieri et al., 2013). Recently, polymerase chain reaction (PCR) has shown great potential as a tool for pathogen detection, as it is a high-throughput approach with a high degree of sensitivity and specificity (Abdissa et al., 2006; Moyo et al., 2007). PCR-based molecular serotyping is a simple and rapid technique for identifying *Salmonella enterica* isolates (Karns et al., 2015).

The bacterial flagellum is a large, complex molecular machine made up of more than 30 different proteins. The membrane protein FlhB is a highly conserved component of the flagellar secretion system (Meshcheryakov et al., 2013), and it plays an important role in the determination of flagellar hook length and regulation of protein export (Hirano et al., 1994). Most *Salmonella* species possess flagella and exhibit motility. However, *S.* Pullorum and *S.* Gallinarum are two notable exceptions, having been shown lack of motility and flagella (Holt and Chaubal, 1997). Thus, the *flhB* gene of *S.* Pullorum/Gallinarum may own some special features that are different from other serovar.

In the present study, we developed a rapid one-step PCR system to identify *S. enterica* serovar Pullorum/Gallinarum. The approach used one pair of primers targeting *flhB*, which *in silico* analysis showed a deficient region in *Salmonella* Pullorum/Gallinarum, compared with that of other serovars. The specificity and sensitivity of the PCR system were evaluated, and the assay was applied to *Salmonella* strains isolated from one chicken farm.

## Materials and Methods

### Bacterial Strains

A mix of commercially available and previously isolated environmental *Salmonella* and non-*Salmonella* isolates, including *S.* Enteritidis, *S.* Pullorum, *S.* Gallinarum, *S.* Dublin, *S.* Uganda, *S.* Meleagridis, *S.* Anatis, *S.* London, *S.* Rissen, *S.* Derby, *S.* Typhimurium, *S.* Choleraesuis, *S.* Indiana, *S.* Sinstorf, *S.* Newlands, *S.* Muenster, *S.* Yoruba, *S.* Dumfries, *S.* Kentucky, *S.* Agona, *S.* Thompson, *S.* Senftenberg, *S.* Blockley, *S.* Inchpark, *S.* Virchow, *S.* Farsta, *S.* Dabou, *Mycobacterium tuberculosis, Campylobacter jejuni, Brucella abortus, Listeria monocytogenes*, and *Escherichia coli*, were used in this study (**Table 1**). These strains were used for testing the specificity and sensitivity of the PCR system.

**Table 1:** *Salmonella* and non-*Salmonella* strains used to evaluate the specificity and sensitivity of the developed PCR system.

	Strain	Serovar/Species	Source	*flhB*-PCR result (182 bp/379 bp)
*Salmonella*	C50041	Enteritidis	Laboratory stock	–/+
	C50336	Enteritidis	Laboratory stock	–/+
	S06004	Pullorum	Laboratory stock	+/–
	6508	Pullorum	Isolate from chicken	+/–
	SG9	Gallinarum	Wigley et al., 2005	+/–
	SL5928	Dublin	Laboratory stock	–/+
	T3	Uganda	Cai et al., 2016	–/+
	T9	Meleagridis	Li et al., 2016	–/+
	T8	Anatis	Li et al., 2016	–/+
	G2	London	Cai et al., 2016	–/+
	ZX	Rissen	Cai et al., 2016	–/+
	Y7	Derby	Cai et al., 2016	–/+
	Y8	Typhimurium	Li et al., 2016	–/+
	C500	Choleraesuis	Laboratory stock	–/+
	ZH65	Indiana	Cai et al., 2016	–/+
	ZH5	Sinstorf	Laboratory stock	–/+
	ZH10	Newlands	Isolate from cattle	–/+
	ZH24	Muenster	Laboratory stock	–/+
	ZH82	Yoruba	Isolate from pig	–/+
	G449	Dumfries	Laboratory stock	–/+
	G241	Kentucky	Laboratory stock	–/+
	G382	Agona	Laboratory stock	–/+
	ZH35	Thompson	Cai et al., 2016	–/+
	P192	Senftenberg	Laboratory stock	–/+
	G439	Blockley	Laboratory stock	–/+
	G86	Inchpark	Laboratory stock	–/+
	P122	Virchow	Laboratory stock	–/+
	P74	Farsta	Laboratory stock	–/+
	G85	Dabou	Laboratory stock	–/+
Non-*Salmonella*	H37Rv	*Mycobacterium tuberculosis*	ATCC 27294	–/–
	11168	*Campylobacter jejuni*	ATCC 700819	–/–
	110	*Campylobacter jejuni*	Isolate from chicken	–/–
	S19	*Brucella abortus*	Laboratory stock	–/–
	EGDe	*Listeria monocytogenes*	ATCC BAA-679	–/–
	JS15	*Listeria monocytogenes*	Isolate from sheep	–/–
	1314	*Escherichia coli*	Isolate from chicken	–/–
	1352	*Escherichia coli*	Isolate from chicken	–/–

### Isolation and Serotyping of *Salmonella*

Additional *Salmonella* isolates of unknown serovars were isolated from naturally contaminated samples from one chicken farm in Jiangsu, China. *Salmonella* were isolated from floors and feces, and characterized as described elsewhere (Cai et al., 2016; Li et al., 2016). The pre-enrichment step was performed by suspending each sample in 50 mL buffered peptone water (BPW; Difco, BD, Sparks, MD, USA), and incubating samples at 37°C for 16–18 h. Then, 0.1 mL of the broth culture was subcultured in 10 mL subpackaged Rappaport–Vassiliadis (RV) enrichment broth (Difco, BD) at 42°C for 24 h. One loopful of each RV broth culture was streaked onto xylose lysine tergitol 4 (Difco, BD) agar plates, which were incubated at 37°C for 24–48 h. The presumptive *Salmonella* colony was picked from each plate and biochemically confirmed using an API-20E test kit (bioMérieux, Marcy l’Etoile, France). All isolated *Salmonella* strains were serotyped by slide agglutination using specific antisera (Tianrun Bio-Pharmaceutical, Ningbo, China) according to the White–Kauffmann–LeMinor scheme (Grimont and Weill, 2007).

### Bacterial Growth and Genomic DNA Isolation

All of the bacterial strains used in this study were grown in Luria-Bertani broth (Oxoid, Basingstoke, Hampshire, England) or Brain Heart Infusion broth (Becton, Dickinson and Company, Sparks, MD, USA) at 37°C and 180 rpm overnight. Genomic DNA was extracted using a TIANamp Bacterial DNA kit (TianGen, Beijing, China) according to the manufacturer’s instructions. The concentration and purity of the isolated genomic DNA were measured using a NanoDrop ND-1000 (Thermo Scientific, Wilmington, DE, USA), and DNA was subsequently stored at -20°C until use.

### *In silico* Analysis

To develop a PCR- and sequence-based serotyping approach for identifying *S.* Pullorum/Gallinarum, the difference of *flhB* gene between *S.* Pullorum/Gallinarum and non-*S.* Pullorum/Gallinarum was analyzed. *S*. Typhimurium *flhB* nucleotide sequence (GenBank accession no. NC_003197.1 segment 2010283-2011434) was used to search the NCBI non-redundant nucleotide database using the basic local alignment search tool (BLAST) algorithm. The maximum number of aligned sequences to display was set to the maximum value of 20,000, and other parameters were set to default values. Primers to amplify *flhB* were designed and checked using Primer Premier 5 (Premier, Palo Alto, CA, USA).

### PCR Procedure

Polymerase chain reactions were performed in a final volume of 25 μL, containing 100 ng of the isolated genomic DNA, 1 U of *Taq* polymerase (Takara Biotechnology Co., Dalian, China), 1x polymerase buffer, 200 μM each deoxynucleoside triphosphate, and 0.4 μM *flhB* primers. PCR amplifications were performed using a T100 Thermal Cycler (Bio-Rad, Hercules, CA, USA), with an initial denaturation step of 95°C for 5 min, 30 cycles of 95°C for 45 s, 59°C for 45 s, and 72°C for 1 min, followed by a final extension step of 72°C for 10 min. The resulting amplicons were resolved by horizontal electrophoresis on a 1% agarose gel in 1x TAE buffer.

### Specificity of the PCR Primers

The specificity of the *flhB* primers was assessed using genomic DNA from 29 *Salmonella* strains, including 27 different serovars, and eight non-*Salmonella* pathogens as negative control strains (**Table 1**).

### Sensitivity of the PCR Assay

The genomic DNA from *S.* Pullorum strain S06004 was serially diluted from 58.5 ng/μL to 5.85 fg/μL in sterile water. Aliquots (2 μL) of each dilution were used as templates for PCR. The objective of the sensitivity analysis was to define the lowest concentration of genomic DNA that could be detected using the PCR assay.

Overnight culture of *S.* Pullorum strain S06004 was consecutively diluted 10-fold in phosphate buffered saline (PBS), and the colony forming unit (CFU) of each dilution was determined by the plate count method. The pure culture was washed with PBS twice, adjusted to the desired bacterial concentrations 2 × 10^6^–2 × 10 CFU/mL, and boiled in a water bath for 10 min to harvest the bacterial genomic DNA, respectively. Finally, 5 μL of each dilution was used for the PCR method to define the least cells of *S.* Pullorum that could be detected using the PCR assay.

### Application of the PCR Assay for Naturally Contaminated Samples

The PCR assay was evaluated using genomic DNA from *Salmonella* isolates collected from the chicken farm (24 *Salmonella* isolates). The results obtained from the assays were compared with the results of the traditional serotyping.

## Results and Discussion

### Primer Design for *S.* Pullorum/Gallinarum-Specific Detection

**FIGURE 1:**
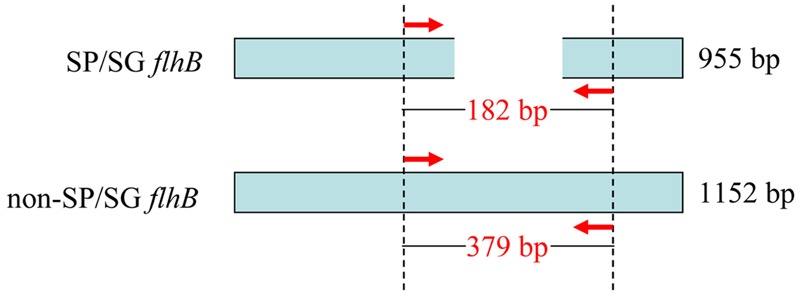
**Schematic for the primer design of *flhB* to distinguish *Salmonella* Pullorum/Gallinarum from other serovars.** *flhB* gene of *S.* Pullorum/Gallinarum has a deficient region compared with that of other serovars, which was exploited to design the primers. The red arrows indicate the positions of the designed primers. The PCR amplifies a product of 182 bp of *S.* Pullorum/Gallinarum and 379 bp of non-*S.* Pullorum/Gallinarum. SP/SG was referred to *S.* Pullorum/Gallinarum.

As the BLAST program is further improved and genomic data continues to be supplemented with newly published *Salmonella* sequences, using comparative genomic analysis to exploit novel serovar-specific genes is becoming more common (Zhai et al., 2014). To develop a PCR- and sequence-based serotyping approach for identifying *S.* Pullorum/Gallinarum, the difference of *flhB* gene between *S.* Pullorum/Gallinarum and non-*S.* Pullorum/Gallinarum was analyzed. *S*. Typhimurium *flhB* nucleotide sequence was used to search the NCBI non-redundant nucleotide database using the BLAST algorithm. The results showed that *flhB* gene of *S.* Pullorum/Gallinarum is 955 bp, covering 83% of other serovars in length (Supplementary Figure S1). Thus, the deficient region of *flhB* could be exploited to distinguish *S.* Pullorum/Gallinarum from other serovars. One pair of oligonucleotide primers covering the deficient region of the *flhB* gene was designed. The sequences of the forward (*flhB*-F) and reverse (*flhB*-R) primers were: 5′-TTC GCG ACG AAT TTA AAG AGA GCG AAG-3′ and 5′-CAG CGT TTA AGC TGC CAG ACC CAG GCC-3′, respectively. These primers amplified a 182-bp fragment of *flhB* of *S.* Pullorum/Gallinarum and a 379-bp fragment of *flhB* of non-*S.* Pullorum/Gallinarum (**Figure 1**). This allowed development of a rapid and reliable one-step PCR assay targeting *flhB* to specifically screen for *S.* Pullorum/Gallinarum. To the best of our knowledge, this is the first single PCR assay to detect *S.* Pullorum/Gallinarum based on the *flhB* gene.

### Specificity of the *flhB*-Based PCR Method for *S.* Pullorum/Gallinarum Detection

**FIGURE 2:**
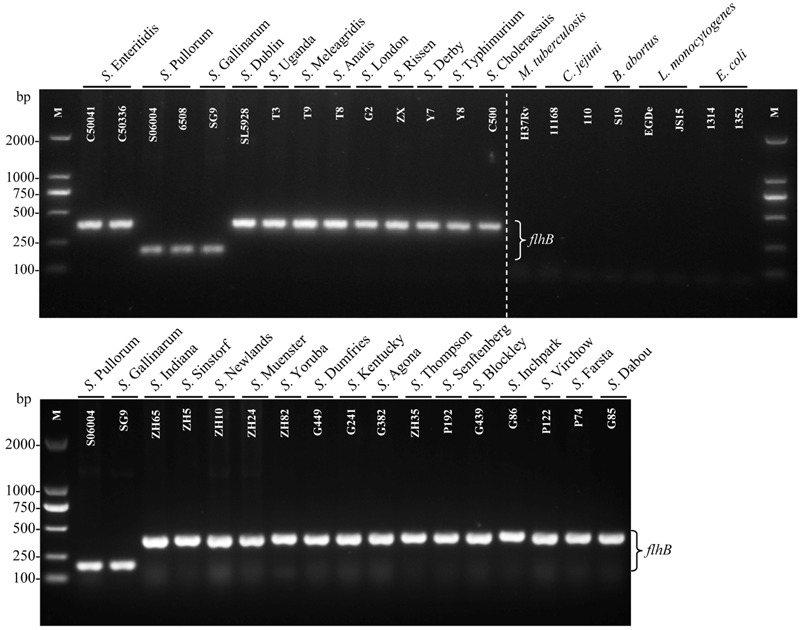
**Specificity of the one-step PCR for the detection of *Salmonella* Pullorum/Gallinarum.** The single PCR assays, using genomic DNA from various *Salmonella* and non-*Salmonella* strains, were conducted using the designed primers targeting *flhB*. The PCR amplifies a product of 182 bp of *S.* Pullorum/Gallinarum. Lane M: DL2000 DNA marker (Takara Biotechnology Co., Dalian, China). Detailed strain information is given in **Table 1**.

The specificity of the PCR system was evaluated using 29 *Salmonella* strains including 27 different serovars and eight non-*Salmonella* strains (**Table 1**). The results revealed that only *S.* Pullorum/Gallinarum generated the specific 182-bp target band. In contrast, the other 25 *Salmonella* serovars generated the specific 379-bp band, and eight non-*Salmonella* strains showed no amplification products using this primer pair (**Figure 2**). Previous studies have shown that *S.* Pullorum and *S.* Gallinarum are lack of motility and flagella (Holt and Chaubal, 1997), which may be related to the difference of *flhB* gene between *S.* Pullorum/Gallinarum and non-*S.* Pullorum/Gallinarum.

### Sensitivity of the *flhB*-Based PCR Method for *S.* Pullorum/Gallinarum Detection

**FIGURE 3:**
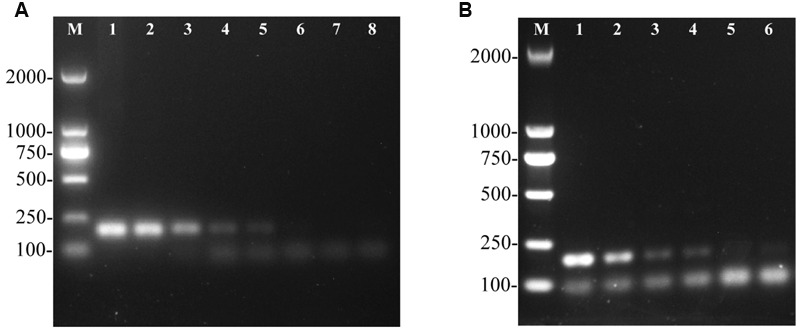
**Sensitivity of the one-step PCR assay for detection of genomic DNA and cells from *Salmonella* Pullorum (strain S06004).** The PCR amplifies a product of 182 bp. Lane M: DL2000 DNA marker (Takara Biotechnology Co., Dalian, China). **(A)** The PCR for the detection of genomic DNA, lanes 1–8, *S.* Pullorum genomic DNA used as template at the following concentrations, respectively: 58.5 ng/μL, 5.85 ng/μL, 585 pg/μL, 58.5 pg/μL, 5.85 pg/μL, 585 fg/μL, 58.5 fg/μL, 5.85 fg/μL; **(B)** The PCR for the detection of *S.* Pullorum cells, lanes 1 to 6, the number of cells per PCR assay, respectively: 10^4^ CFU, 10^3^ CFU, 10^2^ CFU, 10^1^ CFU, 10^0^ and 10^-1^ CFU.

To determine the sensitivity of the PCR assay, genomic DNA from *S.* Pullorum strain S06004 was serially diluted from 58.5 ng/μL to 5.85 fg/μL and used as the template for the assay. The target fragment was amplified at concentrations of 58.5 ng/μL to 5.85 pg/μL DNA (**Figure 3A**). The results suggested that at least 5.85 pg/μL of genomic DNA was required for detection of *S.* Pullorum using this assay, which was slightly lower than previous studies (10 pg/μL) using *Salmonella* genomic DNA (Nithya et al., 2015). On the other hand, a 10-fold serial dilution of *S.* Pullorum cells that ranged from 10^4^ CFU to 10^-1^ CFU per PCR system was evaluated. Using the developed PCR assay on different concentrations of *S.* Pullorum, we validated that the limit of detection was 10 CFU (**Figure 3B**).

### Application of the *S.* Pullorum/Gallinarum-Specific PCR Method

**FIGURE 4:**
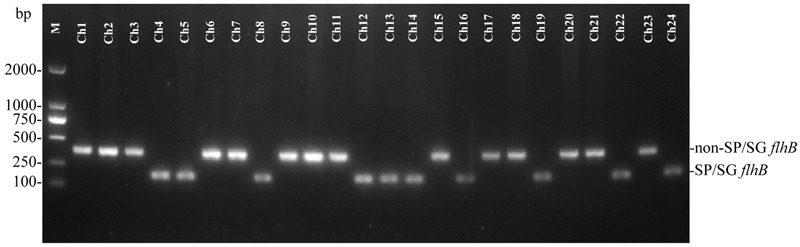
**One-step PCR for the detection of *Salmonella* Pullorum/Gallinarum from *Salmonella* isolates from one chicken farm.** The PCR assay produces a target amplicon of 182 bp of *S.* Pullorum/Gallinarum. Lane M: DL2000 DNA marker (Takara Biotechnology Co., Dalian, China). SP/SG was referred to *S.* Pullorum/Gallinarum. Detailed information on the *Salmonella* isolates is given in **Table 2**.

To evaluate the effectiveness of the established PCR assay, additional *Salmonella* isolates of unknown serovars were isolated from naturally contaminated samples from one chicken farm. The isolated *Salmonella* strains were examined by the developed PCR system. The PCR results showed that 10 samples from the chicken farm contained the specific 182-bp target band of *flhB*, suggesting that these 182-bp *flhB*-positive strains were *S.* Pullorum/Gallinarum (**Figure 4**). PCR results were confirmed by the traditional serotyping, the results of which showed concordance between the two methods for all samples (**Table 2**).

**Table 2:** *Salmonella* strains isolated from one chicken farm to examine the application of the developed PCR system.

Serovar (no. of isolates)	Isolate no.	*flhB*-PCR result (182 bp/379 bp)	Serovar (no. of isolates)	Isolate no.	*flhB*-PCR result (182 bp/379 bp)
Pullorum (10)	Ch4	+/–	Enteritidis (4)	Ch2	–/+
	Ch5	+/–		Ch7	–/+
	Ch8	+/–		Ch11	–/+
	Ch12	+/–		Ch23	–/+
	Ch13	+/–	Indiana (7)	Ch1	–/+
	Ch14	+/–		Ch3	–/+
	Ch16	+/–		Ch9	–/+
	Ch19	+/–		Ch10	–/+
	Ch22	+/–		Ch15	–/+
	Ch24	+/–		Ch17	–/+
Thompson (3)	Ch6	–/+		Ch21	–/+
	Ch18	–/+			
	Ch20	–/+			

*The serotyping of the *Salmonella* isolates was determined based on the traditional serotyping tests according to the White–Kauffmann–Le Minor scheme.*

This PCR assay was also very rapid, taking less than 3 h to complete. The obtained experimental results were in agreement with the comparative genomic analysis used for primer design, and the proposed application was validated by screening for *S.* Pullorum/Gallinarum in samples isolated from the chicken farm. Although traditional serotyping should still be performed, a rapid screen using this *flhB*-based PCR assay may greatly reduce the need for antisera, and may assist in further investigation of *Salmonella* strains. In addition, the combination of PCR-based serotyping and traditional serotyping approaches will allow improved serovar classification of *Salmonella* isolates.

## Conclusion

*flhB*, a gene found a deficient region only in *S.* Pullorum/Gallinarum to be exploited to distinguish this serovar from others, was identified in this study. The difference of *flhB* sequence between *S.* Pullorum/Gallinarum and non-*S.* Pullorum/Gallinarum was used to design a one-step PCR assay specific for *S.* Pullorum/Gallinarum. The assay was used to examine an extensive library of *Salmonella* isolates from one farm, thereby validating the specificity and effectiveness of the method. Our results suggest that this simple and economical PCR system could be used as a rapid diagnostic method for detection of *S.* Pullorum/Gallinarum accurately, especially in a high-throughput screen.

## Author Contributions

ZP and XJ designed the experiments; DX and LS performed the PCR assays; DX, SG, and JT isolated the samples from the chicken farm; SA participated in the data analysis and interpretation; ZP, XJ, and DX wrote the paper. All authors read and approved the final manuscript.

## Conflict of Interest Statement

The authors declare that the research was conducted in the absence of any commercial or financial relationships that could be construed as a potential conflict of interest.
