# Supplementary material for: One-Step PCR Detection of Salmonella Pullorum/Gallinarum Using a Novel Target: The Flagellar Biosynthesis Gene flhB
Source: Front Microbiol. 2016 Nov 22;7:1863. doi: 10.3389/fmicb.2016.01863 (PMC5118417; doi:10.3389/fmicb.2016.01863)
Supplement: Supplementary file 1 [file Image_1.PDF]

BLAST ® » blastn suite » RID-VX0CZ9TS014

BLAST Results

STFlhB

**RID**	VX0CZ9TS014 (Expires on 08-26 13:26 pm)
**Query ID**	lcl|Query_82235
**Description**	STFlhB
**Molecule type**	nucleic acid
**Query Length**	1152
**Database Name**	nr
**Description**	Nucleotide collection (nt)
**Program**	BLASTN 2.5.0+

Graphic Summary

Distribution of 100 Blast Hits on the Query Sequence

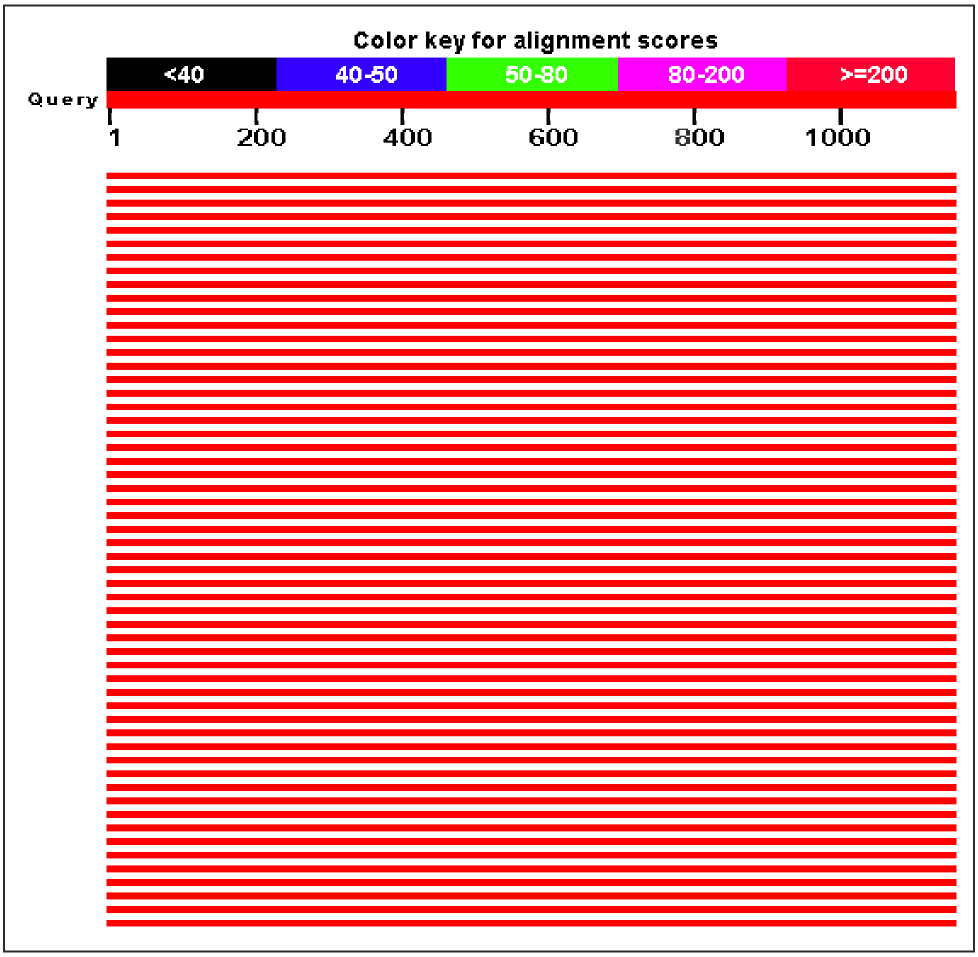

Descriptions

Sequences producing significant alignments:

Description	Max score	Total score	Query cover	E value	Ident	Accession
Salmonella enterica strain FORC_019, complete genome	2128	2128	100%	0.0	100%	CP012396.1
Salmonella enterica subsp. enterica serovar Enteritidis strain OLF-00D989 87-1, complete genome	2128	2128	100%	0.0	100%	CP011942.1
Salmonella enterica subsp. enterica serovar Enteritidis str. SA20094177 genome	2128	2128	100%	0.0	100%	CP007468.2
Salmonella enterica subsp. enterica serovar Enteritidis str. EC20110222 genome	2128	2128	100%	0.0	100%	CP007323.2
Salmonella enterica subsp. enterica serovar Enteritidis str. EC20120685 genome	2128	2128	100%	0.0	100%	CP007339.2
Salmonella enterica subsp. enterica serovar Enteritidis str. EC20120213 genome	2128	2128	100%	0.0	100%	CP007344.2
Salmonella enterica subsp. enterica serovar Enteritidis str. EC20120968 genome	2128	2128	100%	0.0	100%	CP007378.2
Salmonella enterica subsp. enterica serovar Enteritidis str. EC20122022 genome	2128	2128	100%	0.0	100%	CP007412.2
Salmonella enterica subsp. enterica serovar Enteritidis str. SA20082034, complete genome	2128	2128	100%	0.0	100%	CP007425.2
Salmonella enterica subsp. enterica serovar Enteritidis str. EC20111515 genome	2128	2128	100%	0.0	100%	CP007325.2
Salmonella enterica subsp. enterica serovar Enteritidis str. EC20111510 genome	2128	2128	100%	0.0	100%	CP007498.2
Salmonella enterica subsp. enterica serovar Enteritidis str. SA20094301 genome	2128	2128	100%	0.0	100%	CP007469.2
Salmonella enterica subsp. enterica serovar Enteritidis str. SA20084824 genome	2128	2128	100%	0.0	100%	CP007467.2
Salmonella enterica subsp. enterica serovar Enteritidis str. SA20084644 genome	2128	2128	100%	0.0	100%	CP007466.2
Salmonella enterica subsp. enterica serovar Enteritidis str. EC20121747 genome	2128	2128	100%	0.0	100%	CP007464.2
Salmonella enterica subsp. enterica serovar Enteritidis str. EC20120929 genome	2128	2128	100%	0.0	100%	CP007463.2
Salmonella enterica subsp. enterica serovar Enteritidis str. EC20120009 genome	2128	2128	100%	0.0	100%	CP007438.2
Salmonella enterica subsp. enterica serovar Enteritidis str. EC20120051 genome	2128	2128	100%	0.0	100%	CP007433.2
Salmonella enterica subsp. enterica serovar Enteritidis str. EC20121765 genome	2128	2128	100%	0.0	100%	CP007429.2
Salmonella enterica subsp. enterica serovar Enteritidis str. EC20120677 genome	2128	2128	100%	0.0	100%	CP007428.2
Salmonella enterica subsp. enterica serovar Enteritidis str. SA20100239 genome	2128	2128	100%	0.0	100%	CP007427.2
Salmonella enterica subsp. enterica serovar Enteritidis str. EC20100131 genome	2128	2128	100%	0.0	100%	CP007432.2
Salmonella enterica subsp. enterica serovar Enteritidis str. SA20094682 genome	2128	2128	100%	0.0	100%	CP007431.2
Salmonella enterica subsp. enterica serovar Enteritidis str. EC20090195 genome	2128	2128	100%	0.0	100%	CP007430.2
Salmonella enterica subsp. enterica serovar Enteritidis str. SA20085285, complete genome	2128	2128	100%	0.0	100%	CP007426.2
Salmonella enterica subsp. enterica serovar Enteritidis str. EC20120774 genome	2128	2128	100%	0.0	100%	CP007404.2
Salmonella enterica subsp. enterica serovar Enteritidis str. EC20120738 genome	2128	2128	100%	0.0	100%	CP007401.2
Salmonella enterica subsp. enterica serovar						

Enteritidis str. EC20121542 genome	2128	2128	100%	0.0	100%	CP007368.2
Salmonella enterica subsp. enterica serovar Enteritidis str. EC20121541 genome	2128	2128	100%	0.0	100%	CP007367.2
Salmonella enterica subsp. enterica serovar Enteritidis str. EC20121004 genome	2128	2128	100%	0.0	100%	CP007366.2
Salmonella enterica subsp. enterica serovar Enteritidis str. EC20121744 genome	2128	2128	100%	0.0	100%	CP007373.2
Salmonella enterica subsp. enterica serovar Enteritidis str. SA20121703 genome	2128	2128	100%	0.0	100%	CP007372.2
Salmonella enterica subsp. enterica serovar Enteritidis str. EC20121689 genome	2128	2128	100%	0.0	100%	CP007371.2
Salmonella enterica subsp. enterica serovar Enteritidis str. EC20121672 genome	2128	2128	100%	0.0	100%	CP007370.2
Salmonella enterica subsp. enterica serovar Enteritidis str. EC20121671 genome	2128	2128	100%	0.0	100%	CP007369.2
Salmonella enterica subsp. enterica serovar Enteritidis str. EC20090531 genome	2128	2128	100%	0.0	100%	CP007422.2
Salmonella enterica subsp. enterica serovar Enteritidis str. EC20090884 genome	2128	2128	100%	0.0	100%	CP007421.2
Salmonella enterica subsp. enterica serovar Enteritidis str. EC20100103 genome	2128	2128	100%	0.0	100%	CP007420.2
Salmonella enterica subsp. enterica serovar Enteritidis str. EC20120200, complete genome	2128	2128	100%	0.0	100%	CP007434.2
Salmonella enterica subsp. enterica serovar Enteritidis str. EC20130348 genome	2128	2128	100%	0.0	100%	CP007424.2
Salmonella enterica subsp. enterica serovar Enteritidis str. EC20130347 genome	2128	2128	100%	0.0	100%	CP007423.2
Salmonella enterica subsp. enterica serovar Enteritidis str. EC20130346 genome	2128	2128	100%	0.0	100%	CP007419.2
Salmonella enterica subsp. enterica serovar Enteritidis str. EC20130345 genome	2128	2128	100%	0.0	100%	CP007418.2
Salmonella enterica subsp. enterica serovar Enteritidis str. SA20123395 genome	2128	2128	100%	0.0	100%	CP007417.2
Salmonella enterica subsp. enterica serovar Enteritidis str. EC20122045 genome	2128	2128	100%	0.0	100%	CP007416.2
Salmonella enterica subsp. enterica serovar Enteritidis str. EC20122033 genome	2128	2128	100%	0.0	100%	CP007415.2
Salmonella enterica subsp. enterica serovar Enteritidis str. EC20120528 genome	2128	2128	100%	0.0	100%	CP007354.2
Salmonella enterica subsp. enterica serovar Enteritidis str. SA20100349 genome	2128	2128	100%	0.0	100%	CP007361.2
Salmonella enterica subsp. enterica serovar Enteritidis str. EC20100134 genome	2128	2128	100%	0.0	100%	CP007359.2
Salmonella enterica subsp. enterica serovar Enteritidis str. EC20100130 genome	2128	2128	100%	0.0	100%	CP007358.2
Salmonella enterica subsp. enterica serovar Enteritidis str. EC20100100 genome	2128	2128	100%	0.0	100%	CP007357.2
Salmonella enterica subsp. enterica serovar Enteritidis str. EC20121825 genome	2128	2128	100%	0.0	100%	CP007382.2
Salmonella enterica subsp. enterica serovar Enteritidis str. EC20121812 genome	2128	2128	100%	0.0	100%	CP007381.2
Salmonella enterica subsp. enterica serovar Enteritidis str. EC20120970 genome	2128	2128	100%	0.0	100%	CP007380.2
Salmonella enterica subsp. enterica serovar Enteritidis str. EC20120969 genome	2128	2128	100%	0.0	100%	CP007379.2
Salmonella enterica subsp. enterica serovar Enteritidis str. EC20120963 genome	2128	2128	100%	0.0	100%	CP007377.2
Salmonella enterica subsp. enterica serovar Enteritidis str. EC20120927 genome	2128	2128	100%	0.0	100%	CP007376.2
Salmonella enterica subsp. enterica serovar Enteritidis str. EC20120994 genome	2128	2128	100%	0.0	100%	CP007365.2
Salmonella enterica subsp. enterica serovar Enteritidis str. SA20094350 genome	2128	2128	100%	0.0	100%	CP007311.2

Salmonella enterica subsp. enterica serovar Enteritidis str. SA20094079 genome	2128	2128	100%	0.0	100%	CP007310.2
Salmonella enterica subsp. enterica serovar Enteritidis str. SA20093977 genome	2128	2128	100%	0.0	100%	CP007309.2
Salmonella enterica subsp. enterica serovar Enteritidis str. SA20093950 genome	2128	2128	100%	0.0	100%	CP007308.2
Salmonella enterica subsp. enterica serovar Enteritidis str. SA20093788 genome	2128	2128	100%	0.0	100%	CP007307.2
Salmonella enterica subsp. enterica serovar Enteritidis str. SA20092320 genome	2128	2128	100%	0.0	100%	CP007334.2
Salmonella enterica subsp. enterica serovar Enteritidis str. EC20121177, complete genome	2128	2128	100%	0.0	100%	CP007333.2
Salmonella enterica subsp. enterica serovar Enteritidis str. EC20120555 genome	2128	2128	100%	0.0	100%	CP007364.2
Salmonella enterica subsp. enterica serovar Enteritidis str. EC20120548 genome	2128	2128	100%	0.0	100%	CP007363.2
Salmonella enterica subsp. enterica serovar Enteritidis str. EC20120544 genome	2128	2128	100%	0.0	100%	CP007362.2
Salmonella enterica subsp. enterica serovar Enteritidis str. EC20121750 genome	2128	2128	100%	0.0	100%	CP007396.2
Salmonella enterica subsp. enterica serovar Enteritidis str. EC20121746 genome	2128	2128	100%	0.0	100%	CP007374.2
Salmonella enterica subsp. enterica serovar Enteritidis str. EC20120925 genome	2128	2128	100%	0.0	100%	CP007375.2
Salmonella enterica subsp. enterica serovar Enteritidis str. EC20120775 genome	2128	2128	100%	0.0	100%	CP007405.2
Salmonella enterica subsp. enterica serovar Enteritidis str. EC20100325 genome	2128	2128	100%	0.0	100%	CP007360.2
Salmonella enterica subsp. enterica serovar Enteritidis str. EC20120505 genome	2128	2128	100%	0.0	100%	CP007353.2
Salmonella enterica subsp. enterica serovar Enteritidis str. EC20120498 genome	2128	2128	100%	0.0	100%	CP007352.2
Salmonella enterica subsp. enterica serovar Enteritidis str. EC20120497 genome	2128	2128	100%	0.0	100%	CP007351.2
Salmonella enterica subsp. enterica serovar Enteritidis str. EC20120496 genome	2128	2128	100%	0.0	100%	CP007350.2
Salmonella enterica subsp. enterica serovar Enteritidis str. EC20120469 genome	2128	2128	100%	0.0	100%	CP007349.2
Salmonella enterica subsp. enterica serovar Enteritidis str. EC20120356 genome	2128	2128	100%	0.0	100%	CP007348.2
Salmonella enterica subsp. enterica serovar Enteritidis str. EC20120240 genome	2128	2128	100%	0.0	100%	CP007347.2
Salmonella enterica subsp. enterica serovar Enteritidis str. EC20120229 genome	2128	2128	100%	0.0	100%	CP007346.2
Salmonella enterica subsp. enterica serovar Enteritidis str. EC20120219 genome	2128	2128	100%	0.0	100%	CP007345.2
Salmonella enterica subsp. enterica serovar Enteritidis str. EC20120686 genome	2128	2128	100%	0.0	100%	CP007340.2
Salmonella enterica subsp. enterica serovar Enteritidis str. EC20120597 genome	2128	2128	100%	0.0	100%	CP007338.2
Salmonella enterica subsp. enterica serovar Enteritidis str. EC20120581 genome	2128	2128	100%	0.0	100%	CP007336.2
Salmonella enterica subsp. enterica serovar Enteritidis str. EC20120580 genome	2128	2128	100%	0.0	100%	CP007335.2
Salmonella enterica subsp. enterica serovar Enteritidis str. EC20111576 genome	2128	2128	100%	0.0	100%	CP007328.2
Salmonella enterica subsp. enterica serovar Enteritidis str. EC20111554 genome	2128	2128	100%	0.0	100%	CP007326.2
Salmonella enterica subsp. enterica serovar Enteritidis str. EC20111514 genome	2128	2128	100%	0.0	100%	CP007324.2
Salmonella enterica subsp. enterica serovar Enteritidis str. SA20095440, complete genome	2128	2128	100%	0.0	100%	CP007319.2
Salmonella enterica subsp. enterica serovar Enteritidis str. SA20094389 genome	2128	2128	100%	0.0	100%	CP007314.2

Salmonella enterica subsp. enterica serovar Enteritidis str. SA20094383 genome	2128	2128	100%	0.0	100%	CP007313.2
Salmonella enterica subsp. enterica serovar Enteritidis str. SA20094352 complete genome	2128	2128	100%	0.0	100%	CP007312.2
Salmonella enterica subsp. enterica serovar Enteritidis str. SA20093784 genome	2128	2128	100%	0.0	100%	CP007306.2
Salmonella enterica subsp. enterica serovar Enteritidis str. SA20093543 genome	2128	2128	100%	0.0	100%	CP007305.2
Salmonella enterica subsp. enterica serovar Enteritidis str. SA20093538 genome	2128	2128	100%	0.0	100%	CP007304.2
Salmonella enterica subsp. enterica serovar Enteritidis str. SA20093430 genome	2128	2128	100%	0.0	100%	CP007303.2
Salmonella enterica subsp. enterica serovar Enteritidis str. SA20093421 genome	2128	2128	100%	0.0	100%	CP007302.2
Salmonella enterica subsp. enterica serovar Enteritidis str. EC20100089 genome	2128	2128	100%	0.0	100%	CP007356.2
Salmonella enterica subsp. enterica serovar Enteritidis str. EC20100088 genome	2128	2128	100%	0.0	100%	CP007355.2
Salmonella enterica subsp. enterica serovar Enteritidis str. EC20090530 genome	2128	2128	100%	0.0	100%	CP007298.2
Salmonella enterica subsp. enterica serovar Enteritidis str. SA20090435 genome	2128	2128	100%	0.0	100%	CP007297.2
Salmonella enterica subsp. enterica serovar Enteritidis str. SA20090419 genome	2128	2128	100%	0.0	100%	CP007296.2
Salmonella enterica subsp. enterica serovar Enteritidis str. SA19981522, complete genome	2128	2128	100%	0.0	100%	CP007286.2
Salmonella enterica subsp. enterica serovar Enteritidis str. SA19980677, complete genome	2128	2128	100%	0.0	100%	CP007285.2
Salmonella enterica subsp. enterica serovar Enteritidis str. SA19970769, complete genome	2128	2128	100%	0.0	100%	CP007283.2
Salmonella enterica subsp. enterica serovar Enteritidis str. SA19992322 genome	2128	2128	100%	0.0	100%	CP007291.2
Salmonella enterica subsp. enterica serovar Enteritidis str. SA19983126 genome	2128	2128	100%	0.0	100%	CP007290.2
Salmonella enterica subsp. enterica serovar Enteritidis str. SA19982831 genome	2128	2128	100%	0.0	100%	CP007289.2
Salmonella enterica subsp. enterica serovar Enteritidis str. SA19981857, complete genome	2128	2128	100%	0.0	100%	CP007288.2
Salmonella enterica subsp. enterica serovar Enteritidis str. SA19930684 genome	2128	2128	100%	0.0	100%	CP007277.2
Salmonella enterica subsp. enterica serovar Enteritidis str. SA20084384 genome	2128	2128	100%	0.0	100%	CP007295.2
Salmonella enterica subsp. enterica serovar Enteritidis str. SA20083636, complete genome	2128	2128	100%	0.0	100%	CP007294.2
Salmonella enterica subsp. enterica serovar Enteritidis str. SA20083456 genome	2128	2128	100%	0.0	100%	CP007293.2
Salmonella enterica subsp. enterica serovar Enteritidis str. SA19994216, complete genome	2128	2128	100%	0.0	100%	CP007292.2
Salmonella enterica subsp. enterica serovar Enteritidis str. SA19970510, complete genome	2128	2128	100%	0.0	100%	CP007282.2
Salmonella enterica subsp. enterica serovar Enteritidis str. SA19961622 genome	2128	2128	100%	0.0	100%	CP007281.2
Salmonella enterica subsp. enterica serovar Enteritidis str. SA19960848, complete genome	2128	2128	100%	0.0	100%	CP007280.2
Salmonella enterica subsp. enterica serovar Enteritidis str. SA19943269 genome	2128	2128	100%	0.0	100%	CP007279.2
Salmonella enterica subsp. enterica serovar Enteritidis str. SA19942384 genome	2128	2128	100%	0.0	100%	CP007278.2
Salmonella enterica subsp. enterica serovar Enteritidis str. SA20091739 genome	2128	2128	100%	0.0	100%	CP007301.2
Salmonella enterica subsp. enterica serovar Enteritidis str. SA20090877 genome	2128	2128	100%	0.0	100%	CP007300.2
Salmonella enterica subsp. enterica serovar Enteritidis str. SA20093266 genome	2128	2128	100%	0.0	100%	CP007274.2

Salmonella enterica subsp. enterica serovar Enteritidis str. EC20121180 genome	2128	2128	100%	0.0	100%	CP007273.2
Salmonella enterica subsp. enterica serovar Enteritidis str. EC20121179 genome	2128	2128	100%	0.0	100%	CP007272.2
Salmonella enterica subsp. enterica serovar Enteritidis str. EC20121178, complete genome	2128	2128	100%	0.0	100%	CP007271.2
Salmonella enterica subsp. enterica serovar Enteritidis str. EC20121176 genome	2128	2128	100%	0.0	100%	CP007270.2
Salmonella enterica subsp. enterica serovar Enteritidis str. EC20122031 genome	2128	2128	100%	0.0	100%	CP007414.2
Salmonella enterica subsp. enterica serovar Enteritidis str. EC20122026 genome	2128	2128	100%	0.0	100%	CP007413.2
Salmonella enterica subsp. enterica serovar Enteritidis str. EC20121990 genome	2128	2128	100%	0.0	100%	CP007411.2
Salmonella enterica subsp. enterica serovar Enteritidis str. SA19971331 genome	2128	2128	100%	0.0	100%	CP007284.2
Salmonella enterica subsp. enterica serovar Enteritidis str. EC20110223 genome	2128	2128	100%	0.0	100%	CP007266.2
Salmonella enterica subsp. enterica serovar Enteritidis str. EC20120918 genome	2128	2128	100%	0.0	100%	CP007408.2
Salmonella enterica subsp. enterica serovar Enteritidis str. EC20120917 genome	2128	2128	100%	0.0	100%	CP007407.2
Salmonella enterica subsp. enterica serovar Enteritidis str. EC20120776 genome	2128	2128	100%	0.0	100%	CP007406.2
Salmonella enterica subsp. enterica serovar Enteritidis str. EC20120773 genome	2128	2128	100%	0.0	100%	CP007403.2
Salmonella enterica subsp. enterica serovar Enteritidis str. EC20120765 genome	2128	2128	100%	0.0	100%	CP007402.2
Salmonella enterica subsp. enterica serovar Enteritidis str. EC20120734 genome	2128	2128	100%	0.0	100%	CP007400.2
Salmonella enterica subsp. enterica serovar Enteritidis str. EC20121753 genome	2128	2128	100%	0.0	100%	CP007398.2
Salmonella enterica subsp. enterica serovar Enteritidis str. EC20121751 genome	2128	2128	100%	0.0	100%	CP007397.2
Salmonella enterica subsp. enterica serovar Enteritidis str. EC20121748 genome	2128	2128	100%	0.0	100%	CP007395.2
Salmonella enterica subsp. enterica serovar Enteritidis str. EC20121989 genome	2128	2128	100%	0.0	100%	CP007388.2
Salmonella enterica subsp. enterica serovar Enteritidis str. EC20121986 genome	2128	2128	100%	0.0	100%	CP007387.2
Salmonella enterica subsp. enterica serovar Enteritidis str. EC20121976 genome	2128	2128	100%	0.0	100%	CP007386.2
Salmonella enterica subsp. enterica serovar Enteritidis str. EC20121970 genome	2128	2128	100%	0.0	100%	CP007385.2
Salmonella enterica subsp. enterica serovar Enteritidis str. EC20121969 genome	2128	2128	100%	0.0	100%	CP007384.2
Salmonella enterica subsp. enterica serovar Enteritidis str. EC20121826 genome	2128	2128	100%	0.0	100%	CP007383.2
Salmonella enterica subsp. enterica serovar Enteritidis str. EC20120722 genome	2128	2128	100%	0.0	100%	CP007343.2
Salmonella enterica subsp. enterica serovar Enteritidis str. EC20120697 genome	2128	2128	100%	0.0	100%	CP007342.2
Salmonella enterica subsp. enterica serovar Enteritidis str. EC20120687 genome	2128	2128	100%	0.0	100%	CP007341.2
Salmonella enterica subsp. enterica serovar Enteritidis str. EC20120007 genome	2128	2128	100%	0.0	100%	CP007331.2
Salmonella enterica subsp. enterica serovar Enteritidis str. EC20120003 genome	2128	2128	100%	0.0	100%	CP007330.2
Salmonella enterica subsp. enterica serovar Enteritidis str. EC20111561 genome	2128	2128	100%	0.0	100%	CP007327.2
Salmonella enterica subsp. enterica serovar Enteritidis str. EC20090332 genome	2128	2128	100%	0.0	100%	CP007322.2
Salmonella enterica subsp. enterica serovar Enteritidis str. EC20090193 genome	2128	2128	100%	0.0	100%	CP007321.2

Salmonella enterica subsp. enterica serovar Enteritidis str. EC20090135 genome	2128	2128	100%	0.0	100%	CP007320.2
Salmonella enterica subsp. enterica serovar Enteritidis str. SA20095309 genome	2128	2128	100%	0.0	100%	CP007318.2
Salmonella enterica subsp. enterica serovar Enteritidis str. SA20094803 genome	2128	2128	100%	0.0	100%	CP007317.2
Salmonella enterica subsp. enterica serovar Enteritidis str. SA20094642 genome	2128	2128	100%	0.0	100%	CP007316.2
Salmonella enterica subsp. enterica serovar Enteritidis str. SA20094521 genome	2128	2128	100%	0.0	100%	CP007315.2
Salmonella enterica subsp. enterica serovar Enteritidis str. EC20120590 genome	2128	2128	100%	0.0	100%	CP007337.2
Salmonella enterica subsp. enterica serovar Enteritidis strain CMCC50041, complete genome	2128	2128	100%	0.0	100%	CP013097.1
Salmonella enterica subsp. enterica serovar Enteritidis strain FORC_007, complete genome	2128	2128	100%	0.0	100%	CP009768.1
Salmonella enterica subsp. enterica serovar Enteritidis strain SEE2, complete genome	2128	2128	100%	0.0	100%	CP011791.1
Salmonella enterica subsp. enterica serovar Enteritidis strain SEE1, complete genome	2128	2128	100%	0.0	100%	CP011790.1
Salmonella enterica subsp. enterica serovar Enteritidis str. EC20120005, complete genome	2128	2128	100%	0.0	100%	CP007267.2
Salmonella enterica subsp. enterica serovar Enteritidis str. EC20120002 genome	2128	2128	100%	0.0	100%	CP007329.2
Salmonella enterica subsp. enterica serovar Enteritidis str. EC20090641, complete genome	2128	2128	100%	0.0	100%	CP007249.2
Salmonella enterica subsp. enterica serovar Enteritidis str. 18569, complete genome	2128	2128	100%	0.0	100%	CP011394.1
Salmonella enterica subsp. enterica serovar Enteritidis strain OLF-SE2-98984-6, complete genome	2128	2128	100%	0.0	100%	CP009084.2
Salmonella enterica subsp. enterica serovar Enteritidis strain OLF-SE3-98983-4, complete genome	2128	2128	100%	0.0	100%	CP009085.2
Salmonella enterica subsp. enterica serovar Enteritidis strain SEJ, complete genome	2128	2128	100%	0.0	100%	CP008928.1
Salmonella enterica subsp. enterica serovar Enteritidis strain OLF-SE6-00219-16, complete genome	2128	2128	100%	0.0	100%	CP009088.1
Salmonella enterica subsp. enterica serovar Enteritidis strain OLF-SE1-1019-1, complete genome	2128	2128	100%	0.0	100%	CP009083.1
Salmonella enterica subsp. enterica serovar Enteritidis strain OLF-SE5-1104-2, complete genome	2128	2128	100%	0.0	100%	CP009087.1
Salmonella enterica subsp. enterica serovar Enteritidis strain OLF-SE4-0317-8, complete genome	2128	2128	100%	0.0	100%	CP009086.1
Salmonella enterica subsp. enterica serovar Enteritidis strain OLF-SE11-10058, complete genome	2128	2128	100%	0.0	100%	CP009093.1
Salmonella enterica subsp. enterica serovar Enteritidis strain OLF-SE10-10052, complete genome	2128	2128	100%	0.0	100%	CP009092.1
Salmonella enterica subsp. enterica serovar Enteritidis strain OLF-SE9-10012, complete genome	2128	2128	100%	0.0	100%	CP009091.1
Salmonella enterica subsp. enterica serovar Enteritidis strain OLF-SE8-1021710, complete genome	2128	2128	100%	0.0	100%	CP009090.1
Salmonella enterica subsp. enterica serovar Enteritidis strain OLF-SE7-100819, complete genome	2128	2128	100%	0.0	100%	CP009089.1
Salmonella enterica subsp. enterica serovar Enteritidis str. 77-1427, complete genome	2128	2128	100%	0.0	100%	CP007598.1

Salmonella enterica subsp. enterica serovar Enteritidis str. CDC_2010K_0968, complete genome	2128	2128	100%	0.0	100%	CP007528.1
Salmonella enterica subsp. enterica serovar Enteritidis strain Durban, complete genome	2128	2128	100%	0.0	100%	CP007507.1
Salmonella enterica subsp. enterica serovar Enteritidis str. EC20090698, complete genome	2128	2128	100%	0.0	100%	CP007248.1
Salmonella enterica subsp. enterica serovar Enteritidis str. EC20110221, complete genome	2128	2128	100%	0.0	100%	CP007247.1
Salmonella enterica subsp. enterica serovar Enteritidis str. EC20100101, complete genome	2128	2128	100%	0.0	100%	CP007246.1
Salmonella enterica subsp. enterica serovar Enteritidis str. EC20120008, complete genome	2128	2128	100%	0.0	100%	CP007245.1
Salmonella enterica subsp. enterica serovar Enteritidis str. EC20110354, complete genome	2128	2128	100%	0.0	100%	CP007175.1
Salmonella enterica subsp. enterica serovar Enteritidis str. EC20111174, complete genome	2128	2128	100%	0.0	100%	CP007253.1
Salmonella enterica subsp. enterica serovar Enteritidis str. EC20111175, complete genome	2128	2128	100%	0.0	100%	CP007252.1
Salmonella enterica subsp. enterica serovar Enteritidis str. EC20110353, complete genome	2128	2128	100%	0.0	100%	CP007251.1
Salmonella enterica subsp. enterica serovar Enteritidis str. EC20110355, complete genome	2128	2128	100%	0.0	100%	CP007250.1
Salmonella enterica subsp. enterica serovar Enteritidis str. SA19940857 genome	2128	2128	100%	0.0	100%	CP007465.1
Salmonella enterica subsp. enterica serovar Enteritidis str. EC20111095, complete genome	2128	2128	100%	0.0	100%	CP007254.1
Salmonella enterica subsp. enterica serovar Enteritidis str. EC20120916 genome	2128	2128	100%	0.0	100%	CP007332.1
Salmonella enterica subsp. enterica serovar Enteritidis str. EC20110356, complete genome	2128	2128	100%	0.0	100%	CP007262.1
Salmonella enterica subsp. enterica serovar Enteritidis str. EC20110357, complete genome	2128	2128	100%	0.0	100%	CP007261.1
Salmonella enterica subsp. enterica serovar Enteritidis str. EC20110358, complete genome	2128	2128	100%	0.0	100%	CP007260.1
Salmonella enterica subsp. enterica serovar Enteritidis str. EC20110359, complete genome	2128	2128	100%	0.0	100%	CP007259.1
Salmonella enterica subsp. enterica serovar Enteritidis str. EC20110360, complete genome	2128	2128	100%	0.0	100%	CP007258.1
Salmonella enterica subsp. enterica serovar Enteritidis str. EC20110361, complete genome	2128	2128	100%	0.0	100%	CP007263.1
Salmonella enterica subsp. enterica serovar Enteritidis str. P125109 complete genome	2128	2128	100%	0.0	100%	AM933172.1
Salmonella enterica subsp. enterica serovar Java strain NCTC5706 genome assembly, chromosome: 1	2117	2117	100%	0.0	99%	LT571437.1
Salmonella enterica subsp. enterica serovar Bareilly str. CFSAN000189, complete genome	2117	2117	100%	0.0	99%	CP006053.1
Salmonella enterica subsp. enterica serovar Heidelberg strain SH13-004, complete genome	2111	2111	100%	0.0	99%	CP016586.1
Salmonella enterica subsp. enterica serovar Heidelberg strain SH14-009, complete genome	2111	2111	100%	0.0	99%	CP016581.1
Salmonella enterica subsp. enterica serovar Heidelberg strain SH13-006, complete genome	2111	2111	100%	0.0	99%	CP016579.1
Salmonella enterica subsp. enterica serovar Heidelberg strain AMR588-04-00437, complete genome	2111	2111	100%	0.0	99%	CP016576.1
Salmonella enterica subsp. enterica serovar Heidelberg strain AMR588-04-00435, complete genome	2111	2111	100%	0.0	99%	CP016573.1
Salmonella enterica subsp. enterica serovar Heidelberg strain AMR588-04-00320, complete genome	2111	2111	100%	0.0	99%	CP016569.1
Salmonella enterica subsp. enterica serovar Heidelberg strain AMR588-04-00318, complete	2111	2111	100%	0.0	99%	CP016565.1

sequence						
Salmonella enterica subsp. enterica serovar Heidelberg strain A3EZ223, complete genome	2111	2111	100%	0.0	99%	CP016563.1
Salmonella enterica subsp. enterica serovar Heidelberg strain A3ES40, complete genome	2111	2111	100%	0.0	99%	CP016561.1
Salmonella enterica subsp. enterica serovar Heidelberg strain 09-036813-1A, complete genome	2111	2111	100%	0.0	99%	CP016525.1
Salmonella enterica subsp. enterica serovar Heidelberg strain SA01AB09084001, complete genome	2111	2111	100%	0.0	99%	CP016530.1
Salmonella enterica subsp. enterica serovar Heidelberg strain SA02DT09004001, complete genome	2111	2111	100%	0.0	99%	CP016521.1
Salmonella enterica subsp. enterica serovar Heidelberg strain CE-R2-11-0435, complete genome	2111	2111	100%	0.0	99%	CP016517.1
Salmonella enterica subsp. enterica serovar Heidelberg strain 11-004736-1-7, complete genome	2111	2111	100%	0.0	99%	CP016514.1
Salmonella enterica subsp. enterica serovar Heidelberg strain SH14-028, complete genome	2111	2111	100%	0.0	99%	CP016510.1
Salmonella enterica subsp. enterica serovar Heidelberg strain SH12-003, complete genome	2111	2111	100%	0.0	99%	CP016507.1
Salmonella enterica subsp. enterica serovar Heidelberg strain SH12-007, complete genome	2111	2111	100%	0.0	99%	CP016504.1
Salmonella enterica subsp. enterica serovar Anatum str. USDA-ARS-USMARC-1735, complete genome	2111	2111	100%	0.0	99%	CP007584.2
Salmonella enterica subsp. enterica serovar Anatum str. USDA-ARS-USMARC-1781, complete genome	2111	2111	100%	0.0	99%	CP014666.1
Salmonella enterica subsp. enterica serovar Anatum str. USDA-ARS-USMARC-1766, complete genome	2111	2111	100%	0.0	99%	CP014665.1
Salmonella enterica subsp. enterica serovar Anatum str. USDA-ARS-USMARC-1728, complete genome	2111	2111	100%	0.0	99%	CP014664.1
Salmonella enterica subsp. enterica serovar Anatum str. USDA-ARS-USMARC-1677, complete genome	2111	2111	100%	0.0	99%	CP014663.1
Salmonella enterica subsp. enterica serovar Anatum str. USDA-ARS-USMARC-1783, complete sequence	2111	2111	100%	0.0	99%	CP014661.1
Salmonella enterica subsp. enterica serovar Anatum str. USDA-ARS-USMARC-1765, complete genome	2111	2111	100%	0.0	99%	CP014659.1
Salmonella enterica subsp. enterica serovar Anatum str. USDA-ARS-USMARC-1736, complete genome	2111	2111	100%	0.0	99%	CP014657.1
Salmonella enterica subsp. enterica serovar Anatum str. USDA-ARS-USMARC-1727, complete genome	2111	2111	100%	0.0	99%	CP014621.1
Salmonella enterica subsp. enterica serovar Anatum str. USDA-ARS-USMARC-1676, complete genome	2111	2111	100%	0.0	99%	CP014620.1
Salmonella enterica subsp. enterica serovar Typhimurium strain FORC_020, complete genome	2111	2111	100%	0.0	99%	CP012144.1
Salmonella enterica subsp. enterica serovar Anatum strain GT-38, complete genome	2111	4223	100%	0.0	99%	CP013226.1
Salmonella enterica subsp. enterica serovar Anatum strain GT-01, complete genome	2111	2111	100%	0.0	99%	CP013222.1
Salmonella enterica subsp. enterica serovar Heidelberg strain N13-01290, complete genome	2111	2111	100%	0.0	99%	CP012930.1
Salmonella enterica subsp. enterica serovar	2111	2111	100%	0.0	99%	CP012924.1

Heidelberg strain 12-4374, complete genome						
Salmonella enterica subsp. enterica serovar Heidelberg strain SA02DT10168701, complete genome	2111	2111	100%	0.0	99%	CP012921.1
Salmonella enterica subsp. enterica serovar Newport str. CDC 2010K-2159, complete genome	2111	2111	100%	0.0	99%	CP007559.1
Salmonella enterica subsp. enterica serovar Anatum str. USDA-ARS-USMARC-1175, complete genome	2111	2111	100%	0.0	99%	CP007483.1
Salmonella enterica subsp. enterica serovar Anatum str. CDC 06-0532, complete genome	2111	2111	100%	0.0	99%	CP007211.1
Salmonella enterica subsp. enterica serovar Infantis genome assembly SINFA, chromosome : 1	2111	2111	100%	0.0	99%	LN649235.1
Salmonella enterica subsp. enterica serovar Dublin genome assembly SC50_1, chromosome : I	2111	2111	100%	0.0	99%	LK931502.1
Salmonella enterica subsp. enterica serovar Anatum str. ATCC BAA-1592, complete genome	2111	2111	100%	0.0	99%	CP007531.1
Salmonella enterica subsp. enterica serovar Heidelberg str. CFSAN002064, complete genome	2111	2111	100%	0.0	99%	CP005995.1
Salmonella enterica subsp. enterica serovar Bovismorbificans str. 3114 complete genome	2111	2111	100%	0.0	99%	HF969015.2
Salmonella enterica subsp. enterica serovar Heidelberg str. CFSAN002069, complete genome	2111	2111	100%	0.0	99%	CP005390.2
Salmonella enterica subsp. enterica serovar Heidelberg str. 41578, complete genome	2111	2111	100%	0.0	99%	CP004086.1
Salmonella enterica subsp. enterica serovar Heidelberg str. B182, complete genome	2111	2111	100%	0.0	99%	CP003416.1
Salmonella enterica subsp. enterica serovar Dublin str. CT_02021853, complete genome	2111	2111	100%	0.0	99%	CP001144.1
Salmonella enterica subsp. enterica serovar Heidelberg str. SL476, complete genome	2111	2111	100%	0.0	99%	CP001120.1
Salmonella enterica subsp. enterica serovar Tennessee strain CFSAN001387, complete genome	2106	2106	100%	0.0	99%	CP014994.1
Salmonella enterica subsp. enterica serovar Cerro str. CFSAN001588, complete genome	2106	2106	100%	0.0	99%	CP012833.1
Salmonella enterica subsp. enterica serovar Abony str. 0014, complete genome	2106	2106	100%	0.0	99%	CP007534.1
Salmonella enterica subsp. enterica serovar Tennessee str. TXSC_TXSC08-19, complete genome	2106	2106	100%	0.0	99%	CP007505.1
Salmonella enterica subsp. enterica serovar Typhimurium strain NC983, complete genome	2100	2100	100%	0.0	99%	CP015157.1
Salmonella enterica subsp. enterica strain SA972816, complete genome	2100	2100	100%	0.0	99%	CP007484.1
Salmonella enterica subsp. enterica serovar Typhimurium str. CDC H2662, complete genome	2100	2100	100%	0.0	99%	CP014979.1
Salmonella enterica subsp. enterica serovar Typhimurium str. USDA-ARS-USMARC-1896, complete genome	2100	2100	100%	0.0	99%	CP014977.1
Salmonella enterica subsp. enterica serovar Typhimurium str. CDC 2009K-1640, complete genome	2100	2100	100%	0.0	99%	CP014975.1
Salmonella enterica subsp. enterica serovar Typhimurium str. USDA-ARS-USMARC-1898, complete genome	2100	2100	100%	0.0	99%	CP014971.1
Salmonella enterica subsp. enterica serovar Typhimurium str. USDA-ARS-USMARC-1808, complete genome	2100	2100	100%	0.0	99%	CP014969.1
Salmonella enterica subsp. enterica serovar						

Typhimurium str. CDC 2011K-1702, complete genome	2100	2100	100%	0.0	99%	CP014967.1
Salmonella enterica subsp. enterica serovar Typhimurium str. CDC 2010K-1587, complete genome	2100	2100	100%	0.0	99%	CP014965.1
Salmonella enterica subsp. enterica serovar Typhimurium str. CDC 2009K-2059, complete genome	2100	2100	100%	0.0	99%	CP014983.1
Salmonella enterica subsp. enterica serovar Typhimurium str. USDA-ARS-USMARC-1810, complete genome	2100	2100	100%	0.0	99%	CP014982.1
Salmonella enterica subsp. enterica serovar Typhimurium str. USDA-ARS-USMARC-1880, complete genome	2100	2100	100%	0.0	99%	CP014981.1
Salmonella enterica subsp. enterica serovar Typhimurium strain RM9437, complete genome	2100	2100	100%	0.0	99%	CP012985.1
Salmonella enterica subsp. enterica serovar Typhimurium strain YU15, complete genome	2100	2100	100%	0.0	99%	CP014358.1
Salmonella enterica subsp. enterica serovar Typhimurium strain SO2, complete genome	2100	2100	100%	0.0	99%	CP014356.1
Salmonella enterica subsp. enterica serovar Typhimurium strain SO3, complete genome	2100	2100	100%	0.0	99%	CP014536.1
Salmonella enterica strain LT2, complete genome	2100	2100	100%	0.0	99%	CP014051.1
Salmonella enterica subsp. enterica serovar Typhimurium isolate SO4698-09 genome assembly, chromosome: I	2100	2100	100%	0.0	99%	LN999997.1
Salmonella enterica subsp. enterica serovar Typhimurium str. LT2, complete genome	2100	2100	100%	0.0	99%	AE006468.2
Salmonella enterica subsp. enterica serovar Typhimurium strain 33676, complete genome	2100	2100	100%	0.0	99%	CP012681.1
Salmonella enterica subsp. enterica strain YU39, complete genome	2100	2100	100%	0.0	99%	CP011428.1
Salmonella enterica subsp. enterica serovar Typhimurium str. CDC 2011K-0870, complete genome	2100	2100	100%	0.0	99%	CP007523.1
Salmonella enterica subsp. enterica serovar Typhimurium genome assembly NCTC13348, chromosome : 1	2100	2100	100%	0.0	99%	LN829401.1
Salmonella enterica subsp. enterica serovar Newport str. CVM 21550, complete genome	2100	2100	100%	0.0	99%	CP010283.1
Salmonella enterica subsp. enterica serovar Newport str. CVM 21538, complete genome	2100	2100	100%	0.0	99%	CP010282.1
Salmonella enterica subsp. enterica serovar Newport str. CVM 22513, complete genome	2100	2100	100%	0.0	99%	CP010281.1
Salmonella enterica subsp. enterica serovar Newport str. CVM 22425, complete genome	2100	2100	100%	0.0	99%	CP010279.1
Salmonella enterica subsp. enterica serovar Newport str. CVM N1543, complete genome	2100	2100	100%	0.0	99%	CP010284.1
Salmonella enterica subsp. enterica serovar Typhimurium str. USDA-ARS-USMARC-1899, complete genome	2100	2100	100%	0.0	99%	CP007235.1
Salmonella enterica subsp. enterica serovar Newport str. CVM 22462, complete genome	2100	2100	100%	0.0	99%	CP010280.1
Salmonella enterica subsp. enterica serovar Newport str. CVM N18486, complete genome	2100	2100	100%	0.0	99%	CP009561.1
Salmonella enterica subsp. enterica serovar Typhimurium strain ATCC 13311, complete genome	2100	2100	100%	0.0	99%	CP009102.1
Salmonella enterica subsp. enterica serovar Choleraesuis strain C500, complete genome	2100	2100	100%	0.0	99%	CP007639.1
Salmonella enterica subsp. enterica serovar Typhimurium strain VNP20009, complete genome	2100	2100	100%	0.0	99%	CP007804.2
Salmonella enterica subsp. enterica serovar Typhimurium str. L-3553 DNA, complete	2100	2100	100%	0.0	99%	AP014565.1

genome						
Salmonella enterica subsp. enterica serovar Typhimurium strain 138736, complete genome	2100	2100	100%	0.0	99%	CP007581.1
Salmonella enterica subsp. enterica serovar Typhimurium str. DT2, complete genome	2100	2100	100%	0.0	99%	HG326213.1
Salmonella enterica subsp. enterica serovar Typhimurium DT104 main chromosome, complete genome	2100	2100	100%	0.0	99%	HF937208.1
Salmonella enterica subsp. enterica serovar Newport str. USMARC-S3124.1, complete genome	2100	2100	100%	0.0	99%	CP006631.1
Salmonella enterica subsp. enterica serovar Typhimurium str. 08-1736, complete genome	2100	2100	100%	0.0	99%	CP006602.1
Salmonella enterica subsp. enterica serovar Typhimurium var. 5- str. CFSAN001921, complete genome	2100	2100	100%	0.0	99%	CP006048.1
Salmonella enterica subsp. enterica serovar Typhimurium str. U288, complete genome	2100	2100	100%	0.0	99%	CP003836.1
Salmonella enterica subsp. enterica serovar Typhimurium str. UK-1, complete genome	2100	2100	100%	0.0	99%	CP002614.1
Salmonella enterica subsp. enterica serovar Typhimurium str. T000240 DNA, complete genome	2100	2100	100%	0.0	99%	AP011957.1
Salmonella enterica subsp. enterica serovar Typhimurium str. 14028S, complete genome	2100	2100	100%	0.0	99%	CP001363.1
Salmonella enterica subsp. enterica serovar Typhimurium str. D23580 complete genome	2100	2100	100%	0.0	99%	FN424405.1
Salmonella enterica subsp. enterica serovar Paratyphi C strain RKS4594, complete genome	2100	2100	100%	0.0	99%	CP000857.1
Salmonella enterica subsp. enterica serovar Newport str. SL254, complete genome	2100	2100	100%	0.0	99%	CP001113.1
Salmonella enterica subsp. enterica serovar Choleraesuis str. SC-B67, complete genome	2100	2100	100%	0.0	99%	AE017220.1
Salmonella enterica subsp. enterica serovar Typhimurium flhB operon encoding flagellar proteins FlhB, FlhA and FlhE, complete cds	2100	2100	100%	0.0	99%	D32203.1
Salmonella enterica subsp. enterica serovar Weltevreden str. 1655, complete genome	2095	2095	100%	0.0	99%	CP014996.1
Salmonella enterica subsp. enterica serovar Typhimurium strain SL1344RX genome	2095	2095	100%	0.0	99%	CP011233.1
Salmonella enterica subsp. enterica serovar Weltevreden genome assembly 99_3134, chromosome : 1	2095	2095	100%	0.0	99%	LN890524.1
Salmonella enterica subsp. enterica serovar Weltevreden genome assembly 98_11262, chromosome : 1	2095	2095	100%	0.0	99%	LN890522.1
Salmonella enterica subsp. enterica serovar Weltevreden genome assembly C2346, chromosome : 1	2095	2095	100%	0.0	99%	LN890520.1
Salmonella enterica subsp. enterica serovar Weltevreden genome assembly 10259, chromosome : 1	2095	2095	100%	0.0	99%	LN890518.1
Salmonella enterica subsp. enterica serovar Typhi strain B/SF/13/03/195, complete genome	2095	2095	100%	0.0	99%	CP012151.1
Salmonella enterica subsp. enterica serovar Typhi strain PM016/13, complete genome	2095	2095	100%	0.0	99%	CP012091.1
Salmonella enterica subsp. enterica serovar Typhimurium strain FORC_015, complete genome	2095	2095	100%	0.0	99%	CP011365.1
Salmonella enterica subsp. enterica serovar Typhi str. CT18, complete chromosome	2095	2095	100%	0.0	99%	AL513382.1
Salmonella enterica subsp. enterica serovar Typhi str. Ty21a, complete genome	2095	2095	100%	0.0	99%	CP002099.1
Salmonella enterica subsp. enterica serovar Typhi str. P-stx-12, complete genome	2095	2095	100%	0.0	99%	CP003278.1
Salmonella enterica subsp. enterica serovar	2095	2095	100%	0.0	99%	CP002487.1

Typhimurium str. ST4/74, complete genome						
Salmonella enterica subsp. enterica serovar Weltevreden str. 2007-60-3289-1 complete genome, contig 23	2095	2095	100%	0.0	99%	FR775210.1
Salmonella enterica subsp. enterica serovar Typhimurium SL1344 complete genome	2095	2095	100%	0.0	99%	FQ312003.1
Salmonella enterica subsp. enterica serovar Typhi Ty2, complete genome	2095	2095	100%	0.0	99%	AE014613.1
Salmonella enterica strain C629, complete genome	2089	2089	100%	0.0	99%	CP015724.1
Salmonella enterica subsp. enterica serovar Sloterdijk str. ATCC 15791, complete genome	2089	2089	100%	0.0	99%	CP012349.1
Salmonella enterica subsp. enterica serovar Ouakam strain GNT-01 genome	2089	2089	100%	0.0	99%	CP012038.1
Salmonella enterica subsp. enterica serovar Paratyphi A strain CMCC50093 genome	2089	2089	100%	0.0	99%	CP011967.1
Salmonella enterica subsp. enterica serovar Newport str. USDA-ARS-USMARC-1927, complete genome	2089	2089	100%	0.0	99%	CP007216.1
Salmonella enterica subsp. enterica serovar Paratyphi A strain CMCC 50973, complete genome	2089	2089	100%	0.0	99%	CP009049.1
Salmonella enterica subsp. enterica serovar Paratyphi A strain CMCC 50503, complete genome	2089	2089	100%	0.0	99%	CP009559.1
Salmonella enterica subsp. enterica Serovar Cubana str. CFSAN002050, complete genome	2089	2089	100%	0.0	99%	CP006055.1
Salmonella enterica subsp. enterica serovar Typhimurium str. 798, complete genome	2089	2089	100%	0.0	99%	CP003386.1
Salmonella enterica subsp. enterica serovar Paratyphi A str. AKU_12601 complete genome, strain AKU_12601	2089	2089	100%	0.0	99%	FM200053.1
Salmonella enterica subsp. enterica serovar Paratyphi A str. ATCC 9150, complete genome	2089	2089	100%	0.0	99%	CP000026.1
Salmonella enterica subsp. enterica serovar Newport str. CVM 21554, complete genome	2087	2087	100%	0.0	99%	CP009565.1
Salmonella enterica subsp. enterica serovar Agona str. 392869-2, complete genome	2084	2084	100%	0.0	99%	CP015024.1
Salmonella enterica subsp. enterica serovar Panama str. ATCC 7378, complete genome	2084	2084	100%	0.0	99%	CP012346.1
Salmonella enterica subsp. enterica serovar Agona str. 460004 2-1, complete genome	2084	2084	100%	0.0	99%	CP011259.1
Salmonella enterica subsp. enterica serovar Agona str. 24249, complete genome	2084	2084	100%	0.0	99%	CP006876.1
Salmonella enterica subsp. enterica serovar Agona str. SL483, complete genome	2084	2084	100%	0.0	99%	CP001138.1
Salmonella enterica subsp. enterica serovar Paratyphi B str. SPB7, complete genome	2084	2084	100%	0.0	99%	CP000886.1
Salmonella enterica subsp. enterica serovar Thompson strain RM1986, complete genome	2078	2078	100%	0.0	99%	CP012514.1
Salmonella enterica subsp. enterica serovar Thompson strain RM1984, complete genome	2078	2078	100%	0.0	99%	CP012513.1
Salmonella enterica subsp. enterica serovar Thompson str. ATCC 8391, complete genome	2078	2078	100%	0.0	99%	CP011396.1
Salmonella enterica subsp. enterica serovar Bredeney str. CFSAN001080, complete genome	2078	2078	100%	0.0	99%	CP007533.1
Salmonella enterica subsp. enterica serovar Thompson str. RM6836, complete genome	2078	2078	100%	0.0	99%	CP006717.1
Salmonella enterica subsp. enterica serovar Javiana str. CFSAN001992, complete genome	2078	2078	100%	0.0	99%	CP004027.1
Salmonella enterica subsp. enterica serovar Schwarzengrund str. CVM19633, complete genome	2078	2078	100%	0.0	99%	CP001127.1
Salmonella enterica subsp. enterica serovar						

Senftenberg genome assembly NCTC10384, chromosome : 1	2073	2073	100%	0.0	99%	LN868943.1
Salmonella enterica subsp. enterica serovar Abaetetuba str. ATCC 35640, complete genome	2067	2067	100%	0.0	99%	CP007532.1
Salmonella enterica subsp. enterica serovar Montevideo str. 507440-20, complete genome	2067	2067	100%	0.0	99%	CP007530.1
Salmonella enterica subsp. enterica serovar Choleraesuis str. ATCC 10708, complete genome	2061	2061	100%	0.0	99%	CP012344.1
Salmonella enterica subsp. enterica serovar Montevideo str. USDA-ARS-USMARC-1921, complete genome	2061	2061	100%	0.0	99%	CP007540.1
Salmonella enterica subsp. enterica serovar Montevideo str. USDA-ARS-USMARC-1903, complete genome	2061	2061	100%	0.0	99%	CP007222.1
Salmonella enterica subsp. diarizonae strain 11-01854, complete genome	1879	1879	100%	0.0	96%	CP011292.1
Salmonella enterica subsp. diarizonae strain 11-01853, complete genome	1879	1879	100%	0.0	96%	CP011289.1
Salmonella enterica subsp. diarizonae strain 11-01855, complete genome	1879	1879	100%	0.0	96%	CP011288.1
Salmonella enterica subsp. arizonae serovar 62:z36:- str. RKS2983, complete genome	1735	1735	100%	0.0	94%	CP006693.1
Salmonella enterica subsp. arizonae serovar 62:z4,z23:--, complete genome	1735	1735	100%	0.0	94%	CP000880.1
Salmonella bongori NCTC 12419, culture collection SGSC SARC11, complete genome	1580	1580	100%	0.0	91%	FR877557.1
Salmonella bongori serovar 48:z41:-- str. RKS3044, complete genome	1574	1574	100%	0.0	91%	CP006692.1
Salmonella bongori N268-08, complete genome	1574	1574	100%	0.0	91%	CP006608.1
Salmonella enterica subsp. enterica serovar Pullorum str. ATCC 9120, complete genome	1395	1784	83%	0.0	100%	CP012347.1
Salmonella enterica subsp. enterica serovar Pullorum genome assembly S44987_1, chromosome : I	1395	1784	83%	0.0	100%	LK931482.1
Salmonella enterica subsp. enterica serovar Gallinarum/pullorum str. CDC1983-67, complete genome	1395	1784	83%	0.0	100%	CP003786.1
Salmonella enterica subsp. enterica serovar Pullorum str. S06004, complete genome	1395	1784	83%	0.0	100%	CP006575.1
Salmonella enterica subsp. enterica serovar Gallinarum/pullorum str. RKS5078, complete genome	1395	1784	83%	0.0	100%	CP003047.1
Salmonella enterica subsp. enterica serovar Gallinarum str. 287/91 complete genome	1395	1784	83%	0.0	100%	AM933173.1
S.typhimurium cheZ gene, complete cds cheY gene, 3' end, and flaM gene (put.), 5' end	1153	1153	55%	0.0	99%	M16691.1
Citrobacter koseri genome assembly PRJEB6512_assembly_1, scaffold CONTIG000001	1131	1131	100%	0.0	84%	LK931336.1
Citrobacter koseri ATCC BAA-895, complete genome	1125	1125	100%	0.0	84%	CP000822.1
Citrobacter rodentium ICC168, complete genome	1040	1040	98%	0.0	83%	FN543502.1
Citrobacter freundii strain B38, complete genome	1020	1020	100%	0.0	83%	CP016762.1
Citrobacter freundii strain P10159, complete genome	1009	1009	100%	0.0	83%	CP012554.1
Citrobacter freundii strain CAV1741, complete genome	1003	1003	100%	0.0	82%	CP011657.1
Citrobacter freundii strain CAV1321, complete genome	1003	1003	100%	0.0	82%	CP011612.1
Citrobacter sp. FDAARGOS_156, complete genome	965	965	100%	0.0	82%	CP014030.1

Citrobacter amalonaticus strain FDAARGOS_165, complete genome	952	952	98%	0.0	82%	CP014070.1
Citrobacter amalonaticus strain FDAARGOS_122, complete genome	939	939	100%	0.0	82%	CP014015.1
Citrobacter amalonaticus Y19, complete genome	939	939	100%	0.0	82%	CP011132.1
Escherichia coli strain SF-166, complete genome	904	904	97%	0.0	81%	CP012633.1
Escherichia coli strain SF-173, complete genome	904	904	97%	0.0	81%	CP012631.1
Escherichia coli strain SF-088, complete genome	904	904	97%	0.0	81%	CP012635.1
Escherichia coli strain SF-468, complete genome	904	904	97%	0.0	81%	CP012625.1
Escherichia coli strain NMEC O18, complete genome	904	904	97%	0.0	81%	CP007275.1
Escherichia coli genome assembly NCTC9001, plasmid : 6	904	904	97%	0.0	81%	LN831045.1
Escherichia coli RS218, complete genome	904	904	97%	0.0	81%	CP007149.1
Escherichia coli APEC IMT5155, complete genome	904	904	97%	0.0	81%	CP005930.1
Escherichia coli PMV-1 main chromosome, complete genome	904	904	97%	0.0	81%	HG428755.1
Escherichia coli UM146, complete genome	904	904	97%	0.0	81%	CP002167.1
Escherichia coli IHE3034, complete genome	904	904	97%	0.0	81%	CP001969.1
Escherichia coli S88 chromosome, complete genome	904	904	97%	0.0	81%	CU928161.2
Escherichia coli APEC O1, complete genome	904	904	97%	0.0	81%	CP000468.1
Escherichia coli UTI89, complete genome	904	904	97%	0.0	81%	CP000243.1
Escherichia coli APEC O18, complete genome	898	898	97%	0.0	81%	CP006830.1
Escherichia coli genome assembly FHI72, scaffold scaffold-30_contig-3.2_199093_533186_[organism:Escherichia	898	898	97%	0.0	81%	LM996875.1
Escherichia coli strain UPEC 26-1, complete genome	893	893	97%	0.0	81%	CP016497.1
Escherichia coli strain K-15KW01, complete genome	893	893	97%	0.0	81%	CP016358.1
Escherichia coli genome assembly FHI74, scaffold scaffold-23_contig-0.1_129766_967476_[organism:Escherichia	893	893	97%	0.0	81%	LM996616.1
Escherichia coli ATCC 25922, complete genome	893	893	97%	0.0	81%	CP009072.1
Escherichia coli Nissle 1917, complete genome	893	893	97%	0.0	81%	CP007799.1
Citrobacter freundii CFNIH1, complete genome	893	893	100%	0.0	81%	CP007557.1
Escherichia coli str. 'clone D i14', complete genome	893	893	97%	0.0	81%	CP002212.1
Escherichia coli str. 'clone D i2', complete genome	893	893	97%	0.0	81%	CP002211.1
Escherichia coli ABU 83972, complete genome	893	893	97%	0.0	81%	CP001671.1
Escherichia coli 536, complete genome	893	893	97%	0.0	81%	CP000247.1
Escherichia coli CFT073, complete genome	893	893	97%	0.0	81%	AE014075.1
Escherichia coli strain ECONIH2, complete genome	891	891	97%	0.0	81%	CP014667.1
Escherichia coli strain NGF1, complete genome	887	887	97%	0.0	81%	CP016007.1
Escherichia coli genome assembly FHI23, scaffold scaffold-32_contig-0.0_1_1236124_[organism:Escherichia	887	887	97%	0.0	81%	LM995659.1
Escherichia coli O83:H1 str. NRG 857C, complete genome	887	887	97%	0.0	81%	CP001855.1
Escherichia coli LF82 chromosome, complete sequence	887	887	97%	0.0	81%	CU651637.1

Escherichia coli strain 2013C-4465, complete genome	881	881	97%	0.0	81%	CP015241.1
Escherichia coli strain ST2747, complete genome	881	881	97%	0.0	81%	CP007394.1
Escherichia coli strain ST2747, complete genome	881	881	97%	0.0	81%	CP007392.1
Escherichia coli O55:H7 str. RM12579, complete genome	881	881	97%	0.0	81%	CP003109.1
Escherichia coli O55:H7 str. CB9615, complete genome	881	881	97%	0.0	81%	CP001846.1
Escherichia coli strain TB182A flagellar biosynthesis protein FlhB (ECs2590) gene, complete cds	881	881	97%	0.0	81%	EU899413.1
Escherichia coli O25b:H4, complete genome	876	876	97%	0.0	81%	CP015085.1
Escherichia coli strain Ecol_732, complete genome	876	876	97%	0.0	81%	CP015138.1
Escherichia coli JJ1887, complete genome	876	876	97%	0.0	81%	CP014316.1
Escherichia coli strain ZH063, complete genome	876	876	97%	0.0	81%	CP014522.1
Escherichia coli strain ZH193, complete genome	876	876	97%	0.0	81%	CP014497.1
Escherichia coli strain SaT040, complete geome	876	876	97%	0.0	81%	CP014495.1
Escherichia coli strain G749, complete genome	876	876	97%	0.0	81%	CP014488.1
Escherichia coli strain JJ1897, complete genome	876	876	97%	0.0	81%	CP013837.1
Escherichia coli strain JJ2434, complete genome	876	876	97%	0.0	81%	CP013835.1
Escherichia coli strain CD306, complete genome	876	876	97%	0.0	81%	CP013831.1
Escherichia coli strain uk_P46212, complete sequence	876	876	97%	0.0	81%	CP013658.1
Escherichia coli strain MNCRE44, complete genome	876	876	97%	0.0	81%	CP010876.1
Escherichia coli ST131 strain EC958 chromosome, complete genome	876	876	97%	0.0	81%	HG941718.1
Escherichia coli JJ1886, complete genome	876	876	97%	0.0	81%	CP006784.1
Escherichia coli NA114, complete genome	876	876	97%	0.0	81%	CP002797.2
Escherichia coli strain 493/89 flagellar biosynthesis protein FlhB (ECs2590) gene, complete cds	876	876	97%	0.0	81%	EU899410.1
Escherichia coli SMS-3-5, complete genome	876	876	97%	0.0	81%	CP000970.1
Escherichia coli strain EDL933-1 genome	870	870	97%	0.0	81%	CP015855.1
Escherichia coli O157:H7 strain FRIK944, complete genome	870	870	97%	0.0	81%	CP016625.1
Escherichia coli strain Ecol_745, complete genome	870	870	97%	0.0	81%	CP015074.2
Escherichia coli strain H1827/12, complete genome	870	870	97%	0.0	81%	CP013031.1
Escherichia coli strain 08-00022, complete genome	870	870	97%	0.0	81%	CP013662.1
Escherichia coli strain Eco889, complete genome	870	870	97%	0.0	81%	CP015159.1
Escherichia coli B strain C2566, complete genome	870	870	97%	0.0	81%	CP014268.2
Escherichia coli strain S51, complete genome	870	870	97%	0.0	81%	CP015995.1
Escherichia coli O157:H7 strain FRIK2069, complete genome	870	870	97%	0.0	81%	CP015846.1
Escherichia coli O157:H7 strain FRIK2455, complete genome	870	870	97%	0.0	81%	CP015843.1
Escherichia coli O157:H7 strain FRIK2533, complete genome	870	870	97%	0.0	81%	CP015842.1
Escherichia coli O157 strain 180-PT54, complete						

genome	870	870	97%	0.0	81%	CP015832.1
Escherichia coli O157 strain 644-PT8, complete genome	870	870	97%	0.0	81%	CP015831.1
Escherichia coli strain Ecol_448, complete genome	870	870	97%	0.0	81%	CP015076.1
Escherichia coli strain Ecol_743, complete genome	870	870	97%	0.0	81%	CP015069.1
Escherichia coli strain SRCC 1675, complete genome	870	870	97%	0.0	81%	CP015023.1
Escherichia coli strain 28RC1, complete genome	870	870	97%	0.0	81%	CP015020.1
Shigella sp. PAMC 28760, complete genome	870	870	97%	0.0	81%	CP014768.1
Escherichia coli B strain C3029, complete genome	870	870	97%	0.0	81%	CP014269.1
Escherichia coli O157:H7 strain JEONG-1266, complete genome	870	870	97%	0.0	81%	CP014314.1
Escherichia coli strain RS76 genome	870	870	97%	0.0	81%	CP013048.1
Escherichia coli strain 2009C-3133, complete genome	870	870	97%	0.0	81%	CP013025.1
Escherichia coli O157:H7 strain WS4202, complete genome	870	870	97%	0.0	81%	CP012802.1
Escherichia coli APEC O2, complete genome	870	870	97%	0.0	81%	CP006834.1
Escherichia coli strain C43(DE3), complete genome	870	870	97%	0.0	81%	CP011938.1
Escherichia coli PCN061, complete genome	870	870	97%	0.0	81%	CP006636.1
Escherichia coli O104:H4 str. C227-11, complete genome	870	870	97%	0.0	81%	CP011331.1
Escherichia coli strain BL21 (TaKaRa), complete genome	870	870	97%	0.0	81%	CP010816.1
Escherichia coli ECC-1470, complete genome	870	870	97%	0.0	81%	CP010344.1
Escherichia coli strain C41(DE3), complete genome	870	870	97%	0.0	81%	CP010585.1
Escherichia coli HUSEC2011 complete genome	870	870	97%	0.0	81%	HF572917.2
Escherichia coli O157:H7 str. SS52, complete genome	870	870	97%	0.0	81%	CP010304.1
Escherichia coli genome assembly FHI102, scaffold scaffold-26_contig-3.1_194786_322630_[organism:Escherichia	870	870	97%	0.0	81%	LM995509.1
Escherichia coli genome assembly FHI98, scaffold scaffold-26_contig-5.0_1_360948_[organism:Escherichia	870	870	97%	0.0	81%	LM997363.1
Escherichia coli O157:H7 str. EDL933, complete genome	870	870	97%	0.0	81%	CP008957.1
Escherichia coli O157:H7 str. SS17, complete genome	870	870	97%	0.0	81%	CP008805.1
Escherichia coli O104:H4 str. 2009EL-2071, complete genome	870	870	97%	0.0	81%	CP003301.1
Escherichia coli O104:H4 str. 2011C-3493, complete genome	870	870	97%	0.0	81%	CP003289.1
Escherichia coli O104:H4 str. 2009EL-2050, complete genome	870	870	97%	0.0	81%	CP003297.1
Escherichia coli Xuzhou21, complete genome	870	870	97%	0.0	81%	CP001925.1
Escherichia coli O7:K1 str. CE10, complete genome	870	870	97%	0.0	81%	CP003034.1
Escherichia coli BL21(DE3), complete genome	870	870	97%	0.0	81%	AM946981.2
Escherichia coli BL21(DE3), complete genome	870	870	97%	0.0	81%	CP001509.3
Escherichia coli SE15 DNA, complete genome	870	870	97%	0.0	81%	AP009378.1
Escherichia coli O157:H7 str. TW14359, complete genome	870	870	97%	0.0	81%	CP001368.1
Escherichia coli B str. REL606, complete						

genome	870	870	97%	0.0	81%	CP000819.1
Escherichia coli 'BL21-Gold(DE3)pLysS AG', complete genome	870	870	97%	0.0	81%	CP001665.1
Escherichia coli strain 86-24 flagellar biosynthesis protein FlhB (ECs2590) gene, complete cds	870	870	97%	0.0	81%	EU899411.1
Escherichia coli 55989 chromosome, complete genome	870	870	97%	0.0	81%	CU928145.2
Escherichia coli O157:H7 str. EC4115, complete genome	870	870	97%	0.0	81%	CP001164.1
Escherichia coli ATCC 8739, complete genome	870	870	97%	0.0	81%	CP000946.1
Escherichia coli HS, complete genome	870	870	97%	0.0	81%	CP000802.1
Escherichia coli O157:H7 str. Sakai DNA, complete genome	870	870	97%	0.0	81%	BA000007.2
Escherichia coli strain MVAST0167, complete genome	869	869	97%	0.0	81%	CP014492.1
Escherichia coli O157:H7 EDL933, complete genome	867	867	97%	0.0	81%	AE005174.2
Escherichia coli PCN033, complete genome	865	865	97%	0.0	81%	CP006632.1
Escherichia coli genome assembly FHI40, scaffold scaffold-24_contig-48.20_717154_821891_[organism:Escherichia	865	865	97%	0.0	81%	LM996228.1
Escherichia coli strain ST540, complete genome	865	865	97%	0.0	81%	CP007391.1
Escherichia coli strain ST540, complete genome	865	865	97%	0.0	81%	CP007390.1
Escherichia coli strain ST540, complete genome	865	865	97%	0.0	81%	CP007265.1
Escherichia coli UMNK88, complete genome	865	865	97%	0.0	81%	CP002729.1
Escherichia coli ETEC H10407, complete genome	865	865	97%	0.0	81%	FN649414.1
Escherichia coli 042 complete genome	865	865	97%	0.0	81%	FN554766.1
Escherichia coli IAI39 chromosome, complete genome	865	865	97%	0.0	81%	CU928164.2
Escherichia coli isolate NCTC86EC genome assembly, chromosome: I	859	859	97%	0.0	81%	LT601384.1
Escherichia coli strain EC590, complete genome	859	859	97%	0.0	81%	CP016182.1
Escherichia coli strain GB089, complete genome	859	859	97%	0.0	81%	CP013663.1
Escherichia coli isolate E. coli RL465 genome assembly, chromosome: RL465_chromosome	859	859	97%	0.0	81%	LT594504.1
Escherichia coli strain 09-00049, complete genome	859	859	97%	0.0	81%	CP015228.1
Escherichia coli strain ER1821R, complete genome	859	859	97%	0.0	81%	CP016018.1
Escherichia coli strain 2011C-3911, complete genome	859	859	97%	0.0	81%	CP015240.1
Escherichia coli str. Sanji, complete genome	859	859	97%	0.0	81%	CP011061.1
Escherichia coli str. K-12 substr. MG1655 strain JW5437-1, complete genome	859	859	97%	0.0	81%	CP014348.1
Escherichia coli strain 268-78-1, complete genome	859	859	97%	0.0	81%	CP014092.1
Escherichia coli K-12 strain C3026, complete genome	859	859	97%	0.0	81%	CP014272.1
Escherichia coli K-12 strain DHB4, complete genome	859	859	97%	0.0	81%	CP014270.1
Escherichia coli str. K-12 substr. MG1655, complete genome	859	859	97%	0.0	81%	CP014225.1
Escherichia coli strain SEC470 genome	859	859	97%	0.0	81%	CP013962.1
Escherichia coli strain CQSW20, complete genome	859	859	97%	0.0	81%	CP013253.1

Escherichia coli strain YD786, complete genome	859	859	97%	0.0	81%	CP013112.1
Escherichia coli strain 2012C-4227, complete genome	859	859	97%	0.0	81%	CP013029.1
Escherichia coli K-12 GM4792 Lac-, complete genome	859	859	97%	0.0	81%	CP011343.2
Escherichia coli K-12 GM4792 Lac+, complete genome	859	859	97%	0.0	81%	CP011342.2
Escherichia coli strain K-12 substrain MG1655_TMP32XR2, complete genome	859	859	97%	0.0	81%	CP012870.1
Escherichia coli strain K-12 substrain MG1655_TMP32XR1, complete genome	859	859	97%	0.0	81%	CP012869.1
Escherichia coli str. K-12 substr. MG1655, complete genome	859	859	97%	0.0	81%	CP012868.1
Escherichia coli genome assembly ERS742059, chromosome : I	859	859	97%	0.0	81%	LN877770.1
Escherichia coli strain RR1, complete genome	859	859	97%	0.0	81%	CP011113.1
Escherichia coli strain DH1Ec169, complete genome	859	859	97%	0.0	81%	CP012127.1
Escherichia coli strain DH1Ec104, complete genome	859	859	97%	0.0	81%	CP012126.1
Escherichia coli strain DH1Ec095, complete genome	859	859	97%	0.0	81%	CP012125.1
Escherichia coli ACN001, complete genome	859	859	97%	0.0	81%	CP007442.1
Escherichia coli strain NCM3722, complete genome	859	859	97%	0.0	81%	CP011495.1
Escherichia coli strain 94-3024, complete genome	859	859	97%	0.0	81%	CP009106.2
Escherichia coli strain CFSAN029787, complete genome	859	859	97%	0.0	81%	CP011416.1
Escherichia coli strain SQ2203, complete genome	859	859	97%	0.0	81%	CP011324.1
Escherichia coli strain SQ171, complete genome	859	859	97%	0.0	81%	CP011323.1
Escherichia coli strain SQ110, complete genome	859	859	97%	0.0	81%	CP011322.1
Escherichia coli strain SQ88, complete genome	859	859	97%	0.0	81%	CP011321.1
Escherichia coli strain SQ37, complete genome	859	859	97%	0.0	81%	CP011320.1
Escherichia coli K-12 strain ER3435, complete genome	859	859	97%	0.0	81%	CP010445.1
Escherichia coli K-12 strain ER3475, complete genome	859	859	97%	0.0	81%	CP010444.1
Escherichia coli K-12 strain ER3446, complete genome	859	859	97%	0.0	81%	CP010443.1
Escherichia coli K-12 strain ER3466, complete genome	859	859	97%	0.0	81%	CP010442.1
Escherichia coli K-12 strain ER3445, complete genome	859	859	97%	0.0	81%	CP010441.1
Escherichia coli K-12 strain ER3476, complete genome	859	859	97%	0.0	81%	CP010440.1
Escherichia coli K-12 strain ER3440, complete genome	859	859	97%	0.0	81%	CP010439.1
Escherichia coli K-12 strain ER3454, complete genome	859	859	97%	0.0	81%	CP010438.1
Escherichia coli K-12 genome assembly EcoliK12AG100, chromosome : I	859	859	97%	0.0	81%	LN832404.1
Escherichia coli 1303, complete genome	859	859	97%	0.0	81%	CP009166.1
Synthetic Escherichia coli C321.deltaA substrain rEc.b.dC.12, complete sequence	859	859	97%	0.0	81%	CP010456.1
Synthetic Escherichia coli C321.deltaA substrain rEc.y.dC.46, complete sequence	859	859	97%	0.0	81%	CP010455.1
Escherichia coli strain 789, complete genome	859	859	97%	0.0	81%	CP010315.1
Escherichia coli strain 6409, complete genome	859	859	97%	0.0	81%	CP010371.1

Escherichia coli str. K-12 substr. MG1655, complete genome	859	859	97%	0.0	81%	CP009685.1
Escherichia coli strain RM9387, complete genome	859	859	97%	0.0	81%	CP009104.1
Escherichia coli K-12 strain ER3413, complete genome	859	859	97%	0.0	81%	CP009789.1
Escherichia coli ER2796, complete genome	859	859	97%	0.0	81%	CP009644.1
Escherichia coli FAP1 genome	859	859	97%	0.0	81%	CP009578.1
Escherichia coli genome assembly EcRV308Chr, chromosome : 1	859	859	97%	0.0	81%	LM995446.1
Escherichia coli genome assembly EcHMS174Chr, chromosome : 1	859	859	97%	0.0	81%	LM993812.1
Escherichia coli BW25113, complete genome	859	859	97%	0.0	81%	CP009273.1
Escherichia coli genome assembly FHI99, scaffold scaffold-31_contig-1.0_1_372097_[organism:Escherichia	859	859	97%	0.0	81%	LM997278.1
Escherichia coli genome assembly FHI97, scaffold scaffold-46_contig-0.4_143201_875885_[organism:Escherichia	859	859	97%	0.0	81%	LM997239.1
Escherichia coli genome assembly FHI70, scaffold scaffold-29_contig-11.0_1_208641_[organism:Escherichia	859	859	97%	0.0	81%	LM997124.1
Escherichia coli genome assembly FHI89, scaffold scaffold-20_contig-1.0_1_1073266_[organism:Escherichia	859	859	97%	0.0	81%	LM997039.1
Escherichia coli genome assembly FHI87, scaffold scaffold-23_contig-1.0_1_445869_[organism:Escherichia	859	859	97%	0.0	81%	LM997008.1
Escherichia coli genome assembly FHI85, scaffold scaffold-26_contig-7.0_1_315701_[organism:Escherichia	859	859	97%	0.0	81%	LM996958.1
Escherichia coli genome assembly FHI71, scaffold scaffold-25_contig-2.1_247176_685371_[organism:Escherichia	859	859	97%	0.0	81%	LM996837.1
Escherichia coli genome assembly FHI82, scaffold scaffold-27_contig-2.0_1_415497_[organism:Escherichia	859	859	97%	0.0	81%	LM996799.1
Escherichia coli genome assembly FHI8, scaffold scaffold-33_contig-0.0_1_783257_[organism:Escherichia	859	859	97%	0.0	81%	LM996756.1
Escherichia coli genome assembly FHI79, scaffold scaffold-25_contig-4.1_285641_497257_[organism:Escherichia	859	859	97%	0.0	81%	LM996698.1
Escherichia coli genome assembly FHI75, scaffold scaffold-23_contig-3.0_1_452278_[organism:Escherichia	859	859	97%	0.0	81%	LM996646.1
Escherichia coli genome assembly FHI65, scaffold scaffold-23_contig-3.0_1_516455_[organism:Escherichia	859	859	97%	0.0	81%	LM996534.1
Escherichia coli genome assembly FHI6, scaffold scaffold-27_contig-6.0_1_371677_[organism:Escherichia	859	859	97%	0.0	81%	LM996361.1
Escherichia coli genome assembly FHI4, scaffold scaffold-30_contig-3.0_1_381805_[organism:Escherichia	859	859	97%	0.0	81%	LM995944.1
Escherichia coli genome assembly FHI20, scaffold scaffold-26_contig-0.0_1_1200133_[organism:Escherichia	859	859	97%	0.0	81%	LM995838.1
Escherichia coli genome assembly FHI24, scaffold scaffold-38_contig-2.0_1_502403_[organism:Escherichia	859	859	97%	0.0	81%	LM995758.1
Escherichia coli genome assembly FHI27, scaffold scaffold-27_contig-0.0_1_488507_[organism:Escherichia	859	859	97%	0.0	81%	LM995705.1
Escherichia coli genome assembly FHI59, scaffold scaffold-21A	859	859	97%	0.0	81%	LK999983.1

Escherichia coli genome assembly FHI42, scaffold scaffold-21	859	859	97%	0.0	81%	LK999941.1
Escherichia coli genome assembly FHI100, scaffold scaffold-31	859	859	97%	0.0	81%	LK985432.1
Escherichia coli genome assembly FHI90, scaffold scaffold-21_contig-10.0_1_183019_[organism:Escherichia	859	859	97%	0.0	81%	LM997310.1
Escherichia coli B7A, complete genome	859	859	97%	0.0	81%	CP005998.1
Escherichia coli KLY, complete genome	859	859	97%	0.0	81%	CP008801.1
Escherichia coli O145:H28 str. RM12581, complete genome	859	859	97%	0.0	81%	CP007136.1
Escherichia coli O145:H28 str. RM12761, complete genome	859	859	97%	0.0	81%	CP007133.1
Escherichia coli O145:H28 str. RM13514, complete genome	859	859	97%	0.0	81%	CP006027.1
Escherichia coli O145:H28 str. RM13516, complete genome	859	859	97%	0.0	81%	CP006262.1
Escherichia coli str. K-12 substr. MC4100 complete genome	859	859	97%	0.0	81%	HG738867.1
Synthetic Escherichia coli C321.deltaA, complete sequence	859	859	97%	0.0	81%	CP006698.1
Escherichia coli str. K-12 substr. MG1655, complete genome	859	859	97%	0.0	81%	U00096.3
Escherichia coli LY180, complete genome	859	859	97%	0.0	81%	CP006584.1
Escherichia coli APEC O78, complete genome	859	859	97%	0.0	81%	CP004009.1
Escherichia coli W, complete genome	859	859	97%	0.0	81%	CP002967.1
Escherichia coli KO11FL, complete genome	859	859	97%	0.0	81%	CP002970.1
Escherichia coli KO11, complete genome	859	859	97%	0.0	81%	CP002516.1
Escherichia coli DH1 (ME8569) DNA, complete genome	859	859	97%	0.0	81%	AP012030.1
Escherichia coli W, complete genome	859	859	97%	0.0	81%	CP002185.1
Escherichia coli DH1, complete genome	859	859	97%	0.0	81%	CP001637.1
Escherichia coli O111:H- str. 11128 DNA, complete genome	859	859	97%	0.0	81%	AP010960.1
Escherichia coli O103:H2 str. 12009 DNA, complete genome	859	859	97%	0.0	81%	AP010958.1
Escherichia coli O26:H11 str. 11368 DNA, complete genome	859	859	97%	0.0	81%	AP010953.1
Escherichia coli BW2952, complete genome	859	859	97%	0.0	81%	CP001396.1
Escherichia coli ED1a chromosome, complete genome	859	859	97%	0.0	81%	CU928162.2
Escherichia coli IAI1 chromosome, complete genome	859	859	97%	0.0	81%	CU928160.2
Escherichia coli SE11 DNA, complete genome	859	859	97%	0.0	81%	AP009240.1
Escherichia coli str. K12 substr. DH10B, complete genome	859	859	97%	0.0	81%	CP000948.1
Escherichia coli E24377A, complete genome	859	859	97%	0.0	81%	CP000800.1
Escherichia coli str. K12 substr. W3110 DNA, complete genome	859	859	97%	0.0	81%	AP009048.1
Escherichia coli strain O177:H21, complete genome	854	854	97%	0.0	80%	CP016546.1
Escherichia coli strain ACN002, complete genome	854	854	97%	0.0	80%	CP007491.1
Escherichia coli strain ST648, complete genome	854	854	97%	0.0	80%	CP008697.1
Escherichia coli VR50, complete genome	854	854	97%	0.0	80%	CP011134.1
Escherichia coli O157:H16 strain Santai, complete genome	854	854	97%	0.0	80%	CP007592.1
Escherichia coli strain ECONIH1, complete genome	854	854	97%	0.0	80%	CP009859.1
Escherichia coli genome assembly FHI34,						

scaffold scaffold-17_contig-8.1_6482_122311_[organism:Escherichia	854	854	97%	0.0	80%	LM996485.1
Escherichia coli genome assembly FHI63, scaffold scaffold-41_contig-2.1_17391_311785_[organism:Escherichia	854	854	97%	0.0	80%	LM996460.1
Escherichia coli genome assembly FHI25, scaffold scaffold-25_contig-2.1_3116_432886_[organism:Escherichia	854	854	97%	0.0	80%	LM996317.1
Escherichia coli genome assembly FHI58, scaffold scaffold-26_contig-2.0_1_592239_[organism:Escherichia	854	854	97%	0.0	80%	LM995995.1
Escherichia coli genome assembly FHI30, scaffold scaffold-27_contig-0.0_1_1238179_[organism:Escherichia	854	854	97%	0.0	80%	LM995905.1
Escherichia coli genome assembly FHI29, scaffold scaffold-22_contig-0.0_1_876679_[organism:Escherichia	854	854	97%	0.0	80%	LM995865.1
Escherichia coli genome assembly FHI28, scaffold scaffold-21_contig-0.1_87396_1078369_[organism:Escherichia	854	854	97%	0.0	80%	LM995788.1
Escherichia coli genome assembly StOlav104, scaffold scaffold-71	854	854	97%	0.0	80%	LK931573.1
Escherichia coli P12b, complete genome	854	854	97%	0.0	80%	CP002291.1
Escherichia coli 0127:H6 E2348/69 complete genome, strain E2348/69	854	854	97%	0.0	80%	FM180568.1
Escherichia coli strain 210205630, complete genome	848	848	97%	0.0	80%	CP015912.1
Escherichia coli strain 06-00048, complete genome	848	848	97%	0.0	80%	CP015229.1
Escherichia coli isolate Co6114 genome	846	846	97%	0.0	80%	CP016034.1
Escherichia coli strain MRE600, complete genome	843	843	97%	0.0	80%	CP014197.1
Escherichia coli strain CI5, complete genome	843	843	98%	0.0	80%	CP011018.1
Escherichia coli genome assembly FHI92, scaffold scaffold-22_contig-2.0_1_542344_[organism:Escherichia	843	843	97%	0.0	80%	LM997172.1
Escherichia coli genome assembly FHI9, scaffold scaffold-33_contig-1.0_1_651525_[organism:Escherichia	843	843	97%	0.0	80%	LM997086.1
Escherichia coli genome assembly FHI83, scaffold scaffold-27_contig-0.0_1_839850_[organism:Escherichia	843	843	97%	0.0	80%	LM996927.1
Escherichia coli genome assembly FHI21, scaffold scaffold-29_contig-1.3_219222_681802_[organism:Escherichia	843	843	97%	0.0	80%	LM996580.1
Escherichia coli genome assembly FHI66, scaffold scaffold-32_contig-0.0_1_159744_[organism:Escherichia	843	843	97%	0.0	80%	LM996411.1
Escherichia coli genome assembly FHI14, scaffold scaffold-27_contig-1.1_1917_804052_[organism:Escherichia	843	843	97%	0.0	80%	LM996120.1
Escherichia coli genome assembly FHI15, scaffold scaffold-40_contig-1.0_1_708548_[organism:Escherichia	843	843	97%	0.0	80%	LM995611.1
Escherichia coli genome assembly FHI12, scaffold scaffold-27_contig-0.0_1_720970_[organism:Escherichia	843	843	97%	0.0	80%	LM995554.1
Escherichia coli genome assembly FHI11, scaffold scaffold-25_contig-0.0_1_704808_[organism:Escherichia	843	843	97%	0.0	80%	LM995481.1
Escherichia coli genome assembly FHI7, scaffold scaffold-26_contig-1.0_1_722455_[organism:Escherichia	843	843	97%	0.0	80%	LM997403.1
Escherichia coli UMN026 chromosome, complete genome	843	843	97%	0.0	80%	CU928163.2
Escherichia coli flagellar proteins FlhB (flhB),	843	843	97%	0.0	80%	U88319.1

FlhA (flhA) and FlhE (flhE) genes, complete cds						
Shigella flexneri 4c strain 1205, complete genome	841	841	97%	0.0	80%	CP012140.1
Shigella flexneri 2a strain 981, complete genome	841	841	97%	0.0	80%	CP012137.1
Shigella flexneri 1a strain 0228, complete genome	841	841	97%	0.0	80%	CP012735.1
Shigella flexneri G1663, complete genome	841	841	97%	0.0	80%	CP007037.1
Shigella flexneri genome assembly NCTC1, chromosome : 1	841	841	97%	0.0	80%	LM651928.1
Shigella flexneri Shi06HN006, complete genome	841	841	97%	0.0	80%	CP004057.1
Shigella flexneri 2003036, complete genome	841	841	97%	0.0	80%	CP004056.1
Shigella flexneri 2a str. 301, complete genome	841	841	97%	0.0	80%	AE005674.2
Shigella flexneri 2002017, complete genome	841	841	97%	0.0	80%	CP001383.1
Shigella flexneri 5 str. 8401, complete genome	841	841	97%	0.0	80%	CP000266.1
Shigella flexneri 2a str. 2457T, complete genome	841	841	97%	0.0	80%	AE014073.1
Enterobacter cloacae subsp. dissolvens SDM, complete genome	817	817	100%	0.0	80%	CP003678.1
Enterobacter cloacae subsp. cloacae ATCC 13047, complete genome	811	811	100%	0.0	79%	CP001918.1
Enterobacter asburiae LF7a, complete genome	808	808	98%	0.0	80%	CP003026.1
Enterobacter cloacae isolate SBP-8 genome	806	806	100%	0.0	79%	CP016906.1
Enterobacter cloacae strain GGT036, complete genome	806	806	100%	0.0	79%	CP009756.1
Escherichia coli strain ST2747, complete genome	784	784	91%	0.0	80%	CP007393.1
Shigella boydii CDC 3083-94, complete genome	774	774	91%	0.0	80%	CP001063.1
Shigella boydii strain ATCC 9210, complete genome	769	769	91%	0.0	80%	CP011511.1
Enterobacter cloacae P101, complete genome	767	767	100%	0.0	79%	CP006580.1
Shigella boydii Sb227, complete genome	763	763	91%	0.0	80%	CP000036.1
Enterobacter cloacae isolate MBRL1077 genome	756	756	100%	0.0	79%	CP014280.1
Leclercia adecarboxylata strain USDA-ARS-USMARC-60222, complete genome	756	756	100%	0.0	79%	CP013990.1
Enterobacter cloacae strain UW5, complete genome	756	756	100%	0.0	79%	CP011798.1
Enterobacter cloacae subsp. cloacae NCTC 9394 draft genome	756	756	100%	0.0	79%	FP929040.1
Enterobacter cloacae complex sp. 35734 chromosome 1, complete sequence	750	750	100%	0.0	79%	CP012162.1
Enterobacter cloacae EcWSU1, complete genome	750	750	100%	0.0	79%	CP002886.1
Enterobacter hormaechei strain CAV1176, complete genome	739	739	100%	0.0	78%	CP011662.1
Enterobacter asburiae strain ATCC 35953, complete sequence	739	739	100%	0.0	78%	CP011863.1
Enterobacter hormaechei subsp. steigerwaltii strain 34977, complete genome	739	739	100%	0.0	78%	CP010376.2
Enterobacter cloacae strain CAV1669, complete genome	739	739	100%	0.0	78%	CP011650.1
Enterobacter cloacae strain CAV1668, complete genome	739	739	100%	0.0	78%	CP011584.1
Enterobacter cloacae strain CAV1411, complete genome	739	739	100%	0.0	78%	CP011581.1
Enterobacter cloacae strain CAV1311, complete genome	739	739	100%	0.0	78%	CP011572.1
Enterobacter cloacae ECNIH2, complete genome	739	739	100%	0.0	78%	CP008823.1

Enterobacter hormaechei subsp. oharae strain 34399, complete genome	734	734	100%	0.0	78%	CP010384.1
Enterobacter hormaechei subsp. steigerwaltii strain 34998, complete genome	728	728	100%	0.0	78%	CP012167.1
Enterobacter hormaechei subsp. oharae strain 34978, complete genome	728	728	100%	0.0	78%	CP012165.1
Enterobacter hormaechei subsp. hormaechei strain 34983, complete genome	728	728	100%	0.0	78%	CP010377.1
Enterobacter asburiae L1, complete genome	717	717	100%	0.0	78%	CP007546.1
Enterobacter sp. ODB01, complete genome	712	712	100%	0.0	78%	CP015227.1
Enterobacter sp. E20, complete genome	712	712	100%	0.0	78%	CP012999.1
Enterobacter cloacae strain ECNIH5, complete genome	712	712	100%	0.0	78%	CP009854.1
Enterobacter cloacae ECR091, complete genome	712	712	100%	0.0	78%	CP008905.1
Enterobacter cloacae ECNIH3, complete genome	712	712	100%	0.0	78%	CP008897.1
Enterobacter cloacae strain ECNIH4, complete genome	701	701	100%	0.0	78%	CP009850.1
Enterobacter cloacae subsp. cloacae ENHKU01, complete genome	701	701	100%	0.0	78%	CP003737.1
Enterobacteriaceae bacterium strain FGI 57, complete genome	691	691	98%	0.0	78%	CP003938.1
Enterobacter cloacae strain colR/S, complete genome	689	689	100%	0.0	78%	CP010512.1
Enterobacter asburiae strain ENIPBJ-CG1, complete genome	689	689	100%	0.0	78%	CP014993.1
Enterobacter asburiae strain CAV1043, complete genome	678	678	100%	0.0	78%	CP011591.1
Shigella sonnei strain FDAARGOS_90, complete genome	669	669	78%	0.0	80%	CP014099.1
Shigella sonnei strain FORC_011, complete genome	669	669	79%	0.0	80%	CP010829.1
Shigella sonnei 53G main chromosome, complete genome	669	669	79%	0.0	80%	HE616528.1
Shigella sonnei Ss046, complete genome	669	669	79%	0.0	80%	CP000038.1
Lelliottia amnigena strain ZB04, complete genome	640	640	100%	3e-179	77%	CP015774.1
Enterobacter sp. 638, complete genome	640	640	100%	3e-179	77%	CP000653.1
Cronobacter universalis NCTC 9529, complete genome	606	606	98%	3e-169	77%	CP012257.1
Cronobacter sakazakii ES15, complete genome	573	573	98%	3e-159	76%	CP003312.1
Cronobacter turicensis z3032 complete genome	573	573	98%	3e-159	76%	FN543093.2
Cronobacter malonaticus LMG 23826, complete genome	568	568	98%	1e-157	76%	CP013940.1
Cronobacter sakazakii strain ATCC 29544, complete genome	568	568	98%	1e-157	76%	CP011047.1
Cronobacter sakazakii CMCC 45402, complete genome	568	568	98%	1e-157	76%	CP006731.1
Cronobacter sakazakii ATCC BAA-894, complete genome	568	568	98%	1e-157	76%	CP000783.1
Cronobacter sakazakii strain NCTC 8155, complete genome	562	562	98%	6e-156	76%	CP012253.1
Cronobacter sakazakii SP291, complete genome	562	562	98%	6e-156	76%	CP004091.1
Cronobacter muytjensii ATCC 51329, complete genome	545	545	98%	7e-151	76%	CP012268.1
Cronobacter condimenti 1330 strain LMG 26250, complete genome	523	523	98%	3e-144	75%	CP012264.1
Cronobacter dublinensis subsp. dublinensis LMG 23823, complete genome	518	518	98%	1e-142	75%	CP012266.1

Kluyvera intermedia strain CAV1151, complete genome	412	412	100%	7e-111	73%	CP011602.1
Pantoea sp. PSNIH2, complete genome	357	357	92%	3e-94	73%	CP009866.1

Alignments

Salmonella enterica strain FORC_019, complete genome
Sequence ID: **gb|CP012396.1|** Length: 4680751 Number of Matches: 1
Range 1: 2734993 to 2736144

Score	Expect	Identities	Gaps	Strand	Frame
2128 bits(1152)	0.0()	1152/1152(100%)	0/1152(0%)	Plus/Minus	

Features:

```
Query  1        GTGGCAGAAGAGAGCGACGACGACAAAACAGAAGCCCCCACACCCCACCGACTTGAAAAA  60
                ||||||||||||||||||||||||||||||||||||||||||||||||||||||||||||
Sbjct  2736144  GTGGCAGAAGAGAGCGACGACGACAAAACAGAAGCCCCCACACCCCACCGACTTGAAAAA  2736085
Query  61       GCGCGGGAAGAAGGGCAGATCCCCCGTTCCAGAGAACTGACCTCACTGCTGATATTGCTG  120
                ||||||||||||||||||||||||||||||||||||||||||||||||||||||||||||
Sbjct  2736084  GCGCGGGAAGAAGGGCAGATCCCCCGTTCCAGAGAACTGACCTCACTGCTGATATTGCTG  2736025
Query  121      GTGGGCGTTTGTATTATTTGGTTCGGCGGCGAGTCGTTAGCGCGGCAACTGGCGGGAATG  180
                ||||||||||||||||||||||||||||||||||||||||||||||||||||||||||||
Sbjct  2736024  GTGGGCGTTTGTATTATTTGGTTCGGCGGCGAGTCGTTAGCGCGGCAACTGGCGGGAATG  2735965
Query  181      CTCTCAGCAGGCCTGCACTTCGATCACCGTATGGTGAACGATCCTAACCTGATCCTGGGG  240
                ||||||||||||||||||||||||||||||||||||||||||||||||||||||||||||
Sbjct  2735964  CTCTCAGCAGGCCTGCACTTCGATCACCGTATGGTGAACGATCCTAACCTGATCCTGGGG  2735905
Query  241      CAGATAATTTTGCTGATTAAAGCGGCGATGATGGCACTGCTACCGCTCATCGCGGGCGTG  300
                ||||||||||||||||||||||||||||||||||||||||||||||||||||||||||||
Sbjct  2735904  CAGATAATTTTGCTGATTAAAGCGGCGATGATGGCACTGCTACCGCTCATCGCGGGCGTG  2735845
Query  301      GTACTGGTGGCGCTTATCTCGCCGGTTATGCTTGGCGGCCTGATTTTTAGCGGTAAGTCG  360
                ||||||||||||||||||||||||||||||||||||||||||||||||||||||||||||
Sbjct  2735844  GTACTGGTGGCGCTTATCTCGCCGGTTATGCTTGGCGGCCTGATTTTTAGCGGTAAGTCG  2735785
Query  361      CTACAGCCAAAATTTTCTAAATTAAACCCGCTGCCGGGAATTAAGCGCATGTTTTCGGCG  420
                ||||||||||||||||||||||||||||||||||||||||||||||||||||||||||||
Sbjct  2735784  CTACAGCCAAAATTTTCTAAATTAAACCCGCTGCCGGGAATTAAGCGCATGTTTTCGGCG  2735725
Query  421      CAGACCGGCGCGGAACTGCTAAAAGCGGTGTTGAAATCCACGCTGGTCGGCTGCGTTACC  480
                ||||||||||||||||||||||||||||||||||||||||||||||||||||||||||||
Sbjct  2735724  CAGACCGGCGCGGAACTGCTAAAAGCGGTGTTGAAATCCACGCTGGTCGGCTGCGTTACC  2735665
Query  481      GGCTTTTATCTCTGGTATCACTGGCCACAAATGATGCGCCTGATGGCGGAGTCGCCGATC  540
                ||||||||||||||||||||||||||||||||||||||||||||||||||||||||||||
Sbjct  2735664  GGCTTTTATCTCTGGTATCACTGGCCACAAATGATGCGCCTGATGGCGGAGTCGCCGATC  2735605
Query  541      GTCGCAATGGGGAATGCGCTGGATCTGGTTGGACTCTGCGCGTTACTGGTGGTACTGGGC  600
                ||||||||||||||||||||||||||||||||||||||||||||||||||||||||||||
Sbjct  2735604  GTCGCAATGGGGAATGCGCTGGATCTGGTTGGACTCTGCGCGTTACTGGTGGTACTGGGC  2735545
Query  601      GTGATTCCGATGGTGGGATTTGACGTGTTTTTCCAGATCTTTAGCCACCTGAAAAAATTA  660
                ||||||||||||||||||||||||||||||||||||||||||||||||||||||||||||
Sbjct  2735544  GTGATTCCGATGGTGGGATTTGACGTGTTTTTCCAGATCTTTAGCCACCTGAAAAAATTA  2735485
Query  661      CGCATGTCGCGGCAGGACATTCGCGACGAATTTAAAGAGAGCGAAGGCGATCCGCATGTT  720
                ||||||||||||||||||||||||||||||||||||||||||||||||||||||||||||
Sbjct  2735484  CGCATGTCGCGGCAGGACATTCGCGACGAATTTAAAGAGAGCGAAGGCGATCCGCATGTT  2735425
Query  721      AAGGGCAAAATTCGCCAGATGCAACGCGCCGCCGCGCAGCGCCGCATGATGGAAGATGTG  780
                ||||||||||||||||||||||||||||||||||||||||||||||||||||||||||||
Sbjct  2735424  AAGGGCAAAATTCGCCAGATGCAACGCGCCGCCGCGCAGCGCCGCATGATGGAAGATGTG  2735365
Query  781      CCGAAAGCGGACGTCATTGTCACTAACCCGACGCACTATTCCGTGGCGCTGCAGTATGAC  840
                ||||||||||||||||||||||||||||||||||||||||||||||||||||||||||||
Sbjct  2735364  CCGAAAGCGGACGTCATTGTCACTAACCCGACGCACTATTCCGTGGCGCTGCAGTATGAC  2735305
Query  841      GAAAACAAAATGAGCGCGCCGAAAGTGGTCGCGAAGGGGGCTGGATTAATAGCGCTGCGC  900
                ||||||||||||||||||||||||||||||||||||||||||||||||||||||||||||
Sbjct  2735304  GAAAACAAAATGAGCGCGCCGAAAGTGGTCGCGAAGGGGGCTGGATTAATAGCGCTGCGC  2735245
Query  901      ATTCGCGAGATCGGCGCTGAACATCGGGTTCCCACTTTAGAAGCGCCGCCGCTGGCGCGG  960
                ||||||||||||||||||||||||||||||||||||||||||||||||||||||||||||
Sbjct  2735244  ATTCGCGAGATCGGCGCTGAACATCGGGTTCCCACTTTAGAAGCGCCGCCGCTGGCGCGG  2735185
Query  961      GCATTATATCGCCACGCCGAAATCGGTCAGCAAATTCCCGGGCAGTTATATGCTGCCGTT  1020
                ||||||||||||||||||||||||||||||||||||||||||||||||||||||||||||
Sbjct  2735184  GCATTATATCGCCACGCCGAAATCGGTCAGCAAATTCCCGGGCAGTTATATGCTGCCGTT  2735125
Query  1021     GCGGAAGTGTTGGCCTGGGTCTGGCAGCTTAAACGCTGGCGGCTTGCGGGCGGGCAACGT  1080
                ||||||||||||||||||||||||||||||||||||||||||||||||||||||||||||
Sbjct  2735124  GCGGAAGTGTTGGCCTGGGTCTGGCAGCTTAAACGCTGGCGGCTTGCGGGCGGGCAACGT  2735065
Query  1081     CCTCCACAACCTGAGAACCTTCCGGTGCCAGAAGCGCTGGATTTTATGAACGAGAAGAAT  1140
                ||||||||||||||||||||||||||||||||||||||||||||||||||||||||||||
Sbjct  2735064  CCTCCACAACCTGAGAACCTTCCGGTGCCAGAAGCGCTGGATTTTATGAACGAGAAGAAT  2735005
Query  1141     ACTGATGGCTAA  1152
                ||||||||||||
Sbjct  2735004  ACTGATGGCTAA  2734993
```

Salmonella enterica subsp. enterica serovar Enteritidis strain OLF-00D989 87-1, complete genome

Sequence ID: **gb|CP011942.1|** Length: 4679487 Number of Matches: 1
Range 1: 1183615 to 1184766

Score	Expect	Identities	Gaps	Strand	Frame
2128 bits(1152)	0.0()	1152/1152(100%)	0/1152(0%)	Plus/Plus	

Features:

```
Query  1        GTGGCAGAAGAGAGCGACGACGACAAAACAGAAGCCCCCACACCCCACCGACTTGAAAAA  60
                ||||||||||||||||||||||||||||||||||||||||||||||||||||||||||||
Sbjct  1183615  GTGGCAGAAGAGAGCGACGACGACAAAACAGAAGCCCCCACACCCCACCGACTTGAAAAA  1183674

Query  61       GCGCGGGAAGAAGGGCAGATCCCCCGTTCCAGAGAACTGACCTCACTGCTGATATTGCTG  120
                ||||||||||||||||||||||||||||||||||||||||||||||||||||||||||||
Sbjct  1183675  GCGCGGGAAGAAGGGCAGATCCCCCGTTCCAGAGAACTGACCTCACTGCTGATATTGCTG  1183734

Query  121      GTGGGCGTTTGTATTATTTGGTTCGGCGGCGAGTCGTTAGCGCGGCAACTGGCGGGAATG  180
                ||||||||||||||||||||||||||||||||||||||||||||||||||||||||||||
Sbjct  1183735  GTGGGCGTTTGTATTATTTGGTTCGGCGGCGAGTCGTTAGCGCGGCAACTGGCGGGAATG  1183794

Query  181      CTCTCAGCAGGCCTGCACTTCGATCACCGTATGGTGAACGATCCTAACCTGATCCTGGGG  240
                ||||||||||||||||||||||||||||||||||||||||||||||||||||||||||||
Sbjct  1183795  CTCTCAGCAGGCCTGCACTTCGATCACCGTATGGTGAACGATCCTAACCTGATCCTGGGG  1183854

Query  241      CAGATAATTTTGCTGATTAAAGCGGCGATGATGGCACTGCTACCGCTCATCGCGGGCGTG  300
                ||||||||||||||||||||||||||||||||||||||||||||||||||||||||||||
Sbjct  1183855  CAGATAATTTTGCTGATTAAAGCGGCGATGATGGCACTGCTACCGCTCATCGCGGGCGTG  1183914

Query  301      GTACTGGTGGCGCTTATCTCGCCGGTTATGCTTGGCGGCCTGATTTTTAGCGGTAAGTCG  360
                ||||||||||||||||||||||||||||||||||||||||||||||||||||||||||||
Sbjct  1183915  GTACTGGTGGCGCTTATCTCGCCGGTTATGCTTGGCGGCCTGATTTTTAGCGGTAAGTCG  1183974

Query  361      CTACAGCCAAAATTTTCTAAATTAAACCCGCTGCCGGGAATTAAGCGCATGTTTTCGGCG  420
                ||||||||||||||||||||||||||||||||||||||||||||||||||||||||||||
Sbjct  1183975  CTACAGCCAAAATTTTCTAAATTAAACCCGCTGCCGGGAATTAAGCGCATGTTTTCGGCG  1184034

Query  421      CAGACCGGCGCGGAACTGCTAAAAGCGGTGTTGAAATCCACGCTGGTCGGCTGCGTTACC  480
                ||||||||||||||||||||||||||||||||||||||||||||||||||||||||||||
Sbjct  1184035  CAGACCGGCGCGGAACTGCTAAAAGCGGTGTTGAAATCCACGCTGGTCGGCTGCGTTACC  1184094

Query  481      GGCTTTTATCTCTGGTATCACTGGCCACAAATGATGCGCCTGATGGCGGAGTCGCCGATC  540
                ||||||||||||||||||||||||||||||||||||||||||||||||||||||||||||
Sbjct  1184095  GGCTTTTATCTCTGGTATCACTGGCCACAAATGATGCGCCTGATGGCGGAGTCGCCGATC  1184154

Query  541      GTCGCAATGGGGAATGCGCTGGATCTGGTTGGACTCTGCGCGTTACTGGTGGTACTGGGC  600
                ||||||||||||||||||||||||||||||||||||||||||||||||||||||||||||
Sbjct  1184155  GTCGCAATGGGGAATGCGCTGGATCTGGTTGGACTCTGCGCGTTACTGGTGGTACTGGGC  1184214

Query  601      GTGATTCCGATGGTGGGATTTGACGTGTTTTTCCAGATCTTTAGCCACCTGAAAAAATTA  660
                ||||||||||||||||||||||||||||||||||||||||||||||||||||||||||||
Sbjct  1184215  GTGATTCCGATGGTGGGATTTGACGTGTTTTTCCAGATCTTTAGCCACCTGAAAAAATTA  1184274

Query  661      CGCATGTCGCGGCAGGACATTCGCGACGAATTTAAAGAGAGCGAAGGCGATCCGCATGTT  720
                ||||||||||||||||||||||||||||||||||||||||||||||||||||||||||||
Sbjct  1184275  CGCATGTCGCGGCAGGACATTCGCGACGAATTTAAAGAGAGCGAAGGCGATCCGCATGTT  1184334

Query  721      AAGGGCAAAATTCGCCAGATGCAACGCGCCGCCGCGCAGCGCCGCATGATGGAAGATGTG  780
                ||||||||||||||||||||||||||||||||||||||||||||||||||||||||||||
Sbjct  1184335  AAGGGCAAAATTCGCCAGATGCAACGCGCCGCCGCGCAGCGCCGCATGATGGAAGATGTG  1184394

Query  781      CCGAAAGCGGACGTCATTGTCACTAACCCGACGCACTATTCCGTGGCGCTGCAGTATGAC  840
                ||||||||||||||||||||||||||||||||||||||||||||||||||||||||||||
Sbjct  1184395  CCGAAAGCGGACGTCATTGTCACTAACCCGACGCACTATTCCGTGGCGCTGCAGTATGAC  1184454

Query  841      GAAAACAAAATGAGCGCGCCGAAAGTGGTCGCGAAGGGGGCTGGATTAATAGCGCTGCGC  900
                ||||||||||||||||||||||||||||||||||||||||||||||||||||||||||||
Sbjct  1184455  GAAAACAAAATGAGCGCGCCGAAAGTGGTCGCGAAGGGGGCTGGATTAATAGCGCTGCGC  1184514

Query  901      ATTCGCGAGATCGGCGCTGAACATCGGGTTCCCACTTTAGAAGCGCCGCCGCTGGCGCGG  960
                ||||||||||||||||||||||||||||||||||||||||||||||||||||||||||||
Sbjct  1184515  ATTCGCGAGATCGGCGCTGAACATCGGGTTCCCACTTTAGAAGCGCCGCCGCTGGCGCGG  1184574

Query  961      GCATTATATCGCCACGCCGAAATCGGTCAGCAAATTCCCGGGCAGTTATATGCTGCCGTT  1020
                ||||||||||||||||||||||||||||||||||||||||||||||||||||||||||||
Sbjct  1184575  GCATTATATCGCCACGCCGAAATCGGTCAGCAAATTCCCGGGCAGTTATATGCTGCCGTT  1184634

Query  1021     GCGGAAGTGTTGGCCTGGGTCTGGCAGCTTAAACGCTGGCGGCTTGCGGGCGGGCAACGT  1080
                ||||||||||||||||||||||||||||||||||||||||||||||||||||||||||||
Sbjct  1184635  GCGGAAGTGTTGGCCTGGGTCTGGCAGCTTAAACGCTGGCGGCTTGCGGGCGGGCAACGT  1184694

Query  1081     CCTCCACAACCTGAGAACCTTCCGGTGCCAGAAGCGCTGGATTTTATGAACGAGAAGAAT  1140
                ||||||||||||||||||||||||||||||||||||||||||||||||||||||||||||
Sbjct  1184695  CCTCCACAACCTGAGAACCTTCCGGTGCCAGAAGCGCTGGATTTTATGAACGAGAAGAAT  1184754

Query  1141     ACTGATGGCTAA  1152
                ||||||||||||
Sbjct  1184755  ACTGATGGCTAA  1184766
```

Salmonella enterica subsp. enterica serovar Enteritidis str. SA20094177 genome
Sequence ID: **gb|CP007468.2|** Length: 4685839 Number of Matches: 1
Range 1: 1183630 to 1184781

Score	Expect	Identities	Gaps	Strand	Frame
2128 bits(1152)	0.0()	1152/1152(100%)	0/1152(0%)	Plus/Plus	

Features:

```
Query  1        GTGGCAGAAGAGAGCGACGACGACAAAACAGAAGCCCCCACACCCCACCGACTTGAAAAA  60
                ||||||||||||||||||||||||||||||||||||||||||||||||||||||||||||
Sbjct  1183630  GTGGCAGAAGAGAGCGACGACGACAAAACAGAAGCCCCCACACCCCACCGACTTGAAAAA  1183689
```

```
Query  61       GCGCGGGAAGAAGGGCAGATCCCCCGTTCCAGAGAACTGACCTCACTGCTGATATTGCTG  120
                ||||||||||||||||||||||||||||||||||||||||||||||||||||||||||||
Sbjct  1183690  GCGCGGGAAGAAGGGCAGATCCCCCGTTCCAGAGAACTGACCTCACTGCTGATATTGCTG  1183749

Query  121      GTGGGCGTTTGTATTATTTGGTTCGGCGGCGAGTCGTTAGCGCGGCAACTGGCGGGAATG  180
                ||||||||||||||||||||||||||||||||||||||||||||||||||||||||||||
Sbjct  1183750  GTGGGCGTTTGTATTATTTGGTTCGGCGGCGAGTCGTTAGCGCGGCAACTGGCGGGAATG  1183809

Query  181      CTCTCAGCAGGCCTGCACTTCGATCACCGTATGGTGAACGATCCTAACCTGATCCTGGGG  240
                ||||||||||||||||||||||||||||||||||||||||||||||||||||||||||||
Sbjct  1183810  CTCTCAGCAGGCCTGCACTTCGATCACCGTATGGTGAACGATCCTAACCTGATCCTGGGG  1183869

Query  241      CAGATAATTTTGCTGATTAAAGCGGCGATGATGGCACTGCTACCGCTCATCGCGGGCGTG  300
                ||||||||||||||||||||||||||||||||||||||||||||||||||||||||||||
Sbjct  1183870  CAGATAATTTTGCTGATTAAAGCGGCGATGATGGCACTGCTACCGCTCATCGCGGGCGTG  1183929

Query  301      GTACTGGTGGCGCTTATCTCGCCGGTTATGCTTGGCGGCCTGATTTTTAGCGGTAAGTCG  360
                ||||||||||||||||||||||||||||||||||||||||||||||||||||||||||||
Sbjct  1183930  GTACTGGTGGCGCTTATCTCGCCGGTTATGCTTGGCGGCCTGATTTTTAGCGGTAAGTCG  1183989

Query  361      CTACAGCCAAAATTTTCTAAATTAAACCCGCTGCCGGGAATTAAGCGCATGTTTTCGGCG  420
                ||||||||||||||||||||||||||||||||||||||||||||||||||||||||||||
Sbjct  1183990  CTACAGCCAAAATTTTCTAAATTAAACCCGCTGCCGGGAATTAAGCGCATGTTTTCGGCG  1184049

Query  421      CAGACCGGCGCGGAACTGCTAAAAGCGGTGTTGAAATCCACGCTGGTCGGCTGCGTTACC  480
                ||||||||||||||||||||||||||||||||||||||||||||||||||||||||||||
Sbjct  1184050  CAGACCGGCGCGGAACTGCTAAAAGCGGTGTTGAAATCCACGCTGGTCGGCTGCGTTACC  1184109

Query  481      GGCTTTTATCTCTGGTATCACTGGCCACAAATGATGCGCCTGATGGCGGAGTCGCCGATC  540
                ||||||||||||||||||||||||||||||||||||||||||||||||||||||||||||
Sbjct  1184110  GGCTTTTATCTCTGGTATCACTGGCCACAAATGATGCGCCTGATGGCGGAGTCGCCGATC  1184169

Query  541      GTCGCAATGGGGAATGCGCTGGATCTGGTTGGACTCTGCGCGTTACTGGTGGTACTGGGC  600
                ||||||||||||||||||||||||||||||||||||||||||||||||||||||||||||
Sbjct  1184170  GTCGCAATGGGGAATGCGCTGGATCTGGTTGGACTCTGCGCGTTACTGGTGGTACTGGGC  1184229

Query  601      GTGATTCCGATGGTGGGATTTGACGTGTTTTTCCAGATCTTTAGCCACCTGAAAAAAATTA  660
                ||||||||||||||||||||||||||||||||||||||||||||||||||||||||||||
Sbjct  1184230  GTGATTCCGATGGTGGGATTTGACGTGTTTTTCCAGATCTTTAGCCACCTGAAAAAAATTA  1184289

Query  661      CGCATGTCGCGGCAGGACATTCGCGACGAATTTAAAGAGAGCGAAGGCGATCCGCATGTT  720
                ||||||||||||||||||||||||||||||||||||||||||||||||||||||||||||
Sbjct  1184290  CGCATGTCGCGGCAGGACATTCGCGACGAATTTAAAGAGAGCGAAGGCGATCCGCATGTT  1184349

Query  721      AAGGGCAAAATTCGCCAGATGCAACGCGCCGCCGCGCAGCGCCGCATGATGGAAGATGTG  780
                ||||||||||||||||||||||||||||||||||||||||||||||||||||||||||||
Sbjct  1184350  AAGGGCAAAATTCGCCAGATGCAACGCGCCGCCGCGCAGCGCCGCATGATGGAAGATGTG  1184409

Query  781      CCGAAAGCGGACGTCATTGTCACTAACCCGACGCACTATTCCGTGGCGCTGCAGTATGAC  840
                ||||||||||||||||||||||||||||||||||||||||||||||||||||||||||||
Sbjct  1184410  CCGAAAGCGGACGTCATTGTCACTAACCCGACGCACTATTCCGTGGCGCTGCAGTATGAC  1184469

Query  841      GAAAACAAAATGAGCGCGCCGAAAGTGGTCGCGAAGGGGGCTGGATTAATAGCGCTGCGC  900
                ||||||||||||||||||||||||||||||||||||||||||||||||||||||||||||
Sbjct  1184470  GAAAACAAAATGAGCGCGCCGAAAGTGGTCGCGAAGGGGGCTGGATTAATAGCGCTGCGC  1184529

Query  901      ATTCGCGAGATCGGCGCTGAACATCGGGTTCCCACTTTAGAAGCGCCGCCGCTGGCGCGG  960
                ||||||||||||||||||||||||||||||||||||||||||||||||||||||||||||
Sbjct  1184530  ATTCGCGAGATCGGCGCTGAACATCGGGTTCCCACTTTAGAAGCGCCGCCGCTGGCGCGG  1184589

Query  961      GCATTATATCGCCACGCCGAAATCGGTCAGCAAATTCCCGGGCAGTTATATGCTGCCGTT  1020
                ||||||||||||||||||||||||||||||||||||||||||||||||||||||||||||
Sbjct  1184590  GCATTATATCGCCACGCCGAAATCGGTCAGCAAATTCCCGGGCAGTTATATGCTGCCGTT  1184649

Query  1021     GCGGAAGTGTTGGCCTGGGTCTGGCAGCTTAAACGCTGGCGGCTTGCGGGCGGGCAACGT  1080
                ||||||||||||||||||||||||||||||||||||||||||||||||||||||||||||
Sbjct  1184650  GCGGAAGTGTTGGCCTGGGTCTGGCAGCTTAAACGCTGGCGGCTTGCGGGCGGGCAACGT  1184709

Query  1081     CCTCCACAACCTGAGAACCTTCCGGTGCCAGAAGCGCTGGATTTTATGAACGAGAAGAAT  1140
                ||||||||||||||||||||||||||||||||||||||||||||||||||||||||||||
Sbjct  1184710  CCTCCACAACCTGAGAACCTTCCGGTGCCAGAAGCGCTGGATTTTATGAACGAGAAGAAT  1184769

Query  1141     ACTGATGGCTAA  1152
                ||||||||||||
Sbjct  1184770  ACTGATGGCTAA  1184781
```

Salmonella enterica subsp. enterica serovar Enteritidis str. EC20110222 genome

Sequence ID: **gb|CP007323.2|** Length: 4686198 Number of Matches: 1

Range 1: 1183630 to 1184781

Score	Expect	Identities	Gaps	Strand	Frame
2128 bits(1152)	0.0()	1152/1152(100%)	0/1152(0%)	Plus/Plus	

Features:

```
Query  1        GTGGCAGAAGAGAGCGACGACGACAAAACAGAAGCCCCCACACCCCACCGACTTGAAAAA  60
                ||||||||||||||||||||||||||||||||||||||||||||||||||||||||||||
Sbjct  1183630  GTGGCAGAAGAGAGCGACGACGACAAAACAGAAGCCCCCACACCCCACCGACTTGAAAAA  1183689

Query  61       GCGCGGGAAGAAGGGCAGATCCCCCGTTCCAGAGAACTGACCTCACTGCTGATATTGCTG  120
                ||||||||||||||||||||||||||||||||||||||||||||||||||||||||||||
Sbjct  1183690  GCGCGGGAAGAAGGGCAGATCCCCCGTTCCAGAGAACTGACCTCACTGCTGATATTGCTG  1183749

Query  121      GTGGGCGTTTGTATTATTTGGTTCGGCGGCGAGTCGTTAGCGCGGCAACTGGCGGGAATG  180
                ||||||||||||||||||||||||||||||||||||||||||||||||||||||||||||
Sbjct  1183750  GTGGGCGTTTGTATTATTTGGTTCGGCGGCGAGTCGTTAGCGCGGCAACTGGCGGGAATG  1183809

Query  181      CTCTCAGCAGGCCTGCACTTCGATCACCGTATGGTGAACGATCCTAACCTGATCCTGGGG  240
                ||||||||||||||||||||||||||||||||||||||||||||||||||||||||||||
Sbjct  1183810  CTCTCAGCAGGCCTGCACTTCGATCACCGTATGGTGAACGATCCTAACCTGATCCTGGGG  1183869

Query  241      CAGATAATTTTGCTGATTAAAGCGGCGATGATGGCACTGCTACCGCTCATCGCGGGCGTG  300
                ||||||||||||||||||||||||||||||||||||||||||||||||||||||||||||
Sbjct  1183870  CAGATAATTTTGCTGATTAAAGCGGCGATGATGGCACTGCTACCGCTCATCGCGGGCGTG  1183929
```

```
Query  301      GTACTGGTGGCGCTTATCTCGCCGGTTATGCTTGGCGGCCTGATTTTTAGCGGTAAGTCG  360
                ||||||||||||||||||||||||||||||||||||||||||||||||||||||||||||
Sbjct  1183930  GTACTGGTGGCGCTTATCTCGCCGGTTATGCTTGGCGGCCTGATTTTTAGCGGTAAGTCG  1183989

Query  361      CTACAGCCAAAATTTTCTAAATTAAACCCGCTGCCGGGAATTAAGCGCATGTTTTCGGCG  420
                ||||||||||||||||||||||||||||||||||||||||||||||||||||||||||||
Sbjct  1183990  CTACAGCCAAAATTTTCTAAATTAAACCCGCTGCCGGGAATTAAGCGCATGTTTTCGGCG  1184049

Query  421      CAGACCGGCGCGGAACTGCTAAAAGCGGTGTTGAAATCCACGCTGGTCGGCTGCGTTACC  480
                ||||||||||||||||||||||||||||||||||||||||||||||||||||||||||||
Sbjct  1184050  CAGACCGGCGCGGAACTGCTAAAAGCGGTGTTGAAATCCACGCTGGTCGGCTGCGTTACC  1184109

Query  481      GGCTTTTATCTCTGGTATCACTGGCCACAAATGATGCGCCTGATGGCGGAGTCGCCGATC  540
                ||||||||||||||||||||||||||||||||||||||||||||||||||||||||||||
Sbjct  1184110  GGCTTTTATCTCTGGTATCACTGGCCACAAATGATGCGCCTGATGGCGGAGTCGCCGATC  1184169

Query  541      GTCGCAATGGGGAATGCGCTGGATCTGGTTGGACTCTGCGCGTTACTGGTGGTACTGGGC  600
                ||||||||||||||||||||||||||||||||||||||||||||||||||||||||||||
Sbjct  1184170  GTCGCAATGGGGAATGCGCTGGATCTGGTTGGACTCTGCGCGTTACTGGTGGTACTGGGC  1184229

Query  601      GTGATTCCGATGGTGGGATTTGACGTGTTTTTCCAGATCTTTAGCCACCTGAAAAAATTA  660
                ||||||||||||||||||||||||||||||||||||||||||||||||||||||||||||
Sbjct  1184230  GTGATTCCGATGGTGGGATTTGACGTGTTTTTCCAGATCTTTAGCCACCTGAAAAAATTA  1184289

Query  661      CGCATGTCGCGGCAGGACATTCGCGACGAATTTAAAGAGAGCGAAGGCGATCCGCATGTT  720
                ||||||||||||||||||||||||||||||||||||||||||||||||||||||||||||
Sbjct  1184290  CGCATGTCGCGGCAGGACATTCGCGACGAATTTAAAGAGAGCGAAGGCGATCCGCATGTT  1184349

Query  721      AAGGGCAAAATTCGCCAGATGCAACGCGCCGCCGCGCAGCGCCGCATGATGGAAGATGTG  780
                ||||||||||||||||||||||||||||||||||||||||||||||||||||||||||||
Sbjct  1184350  AAGGGCAAAATTCGCCAGATGCAACGCGCCGCCGCGCAGCGCCGCATGATGGAAGATGTG  1184409

Query  781      CCGAAAGCGGACGTCATTGTCACTAACCCGACGCACTATTCCGTGGCGCTGCAGTATGAC  840
                ||||||||||||||||||||||||||||||||||||||||||||||||||||||||||||
Sbjct  1184410  CCGAAAGCGGACGTCATTGTCACTAACCCGACGCACTATTCCGTGGCGCTGCAGTATGAC  1184469

Query  841      GAAAACAAAATGAGCGCGCCGAAAGTGGTCGCGAAGGGGGCTGGATTAATAGCGCTGCGC  900
                ||||||||||||||||||||||||||||||||||||||||||||||||||||||||||||
Sbjct  1184470  GAAAACAAAATGAGCGCGCCGAAAGTGGTCGCGAAGGGGGCTGGATTAATAGCGCTGCGC  1184529

Query  901      ATTCGCGAGATCGGCGCTGAACATCGGGTTCCCACTTTAGAAGCGCCGCCGCTGGCGCGG  960
                ||||||||||||||||||||||||||||||||||||||||||||||||||||||||||||
Sbjct  1184530  ATTCGCGAGATCGGCGCTGAACATCGGGTTCCCACTTTAGAAGCGCCGCCGCTGGCGCGG  1184589

Query  961      GCATTATATCGCCACGCCGAAATCGGTCAGCAAATTCCCGGGCAGTTATATGCTGCCGTT  1020
                ||||||||||||||||||||||||||||||||||||||||||||||||||||||||||||
Sbjct  1184590  GCATTATATCGCCACGCCGAAATCGGTCAGCAAATTCCCGGGCAGTTATATGCTGCCGTT  1184649

Query  1021     GCGGAAGTGTTGGCCTGGGTCTGGCAGCTTAAACGCTGGCGGCTTGCGGGCGGGCAACGT  1080
                ||||||||||||||||||||||||||||||||||||||||||||||||||||||||||||
Sbjct  1184650  GCGGAAGTGTTGGCCTGGGTCTGGCAGCTTAAACGCTGGCGGCTTGCGGGCGGGCAACGT  1184709

Query  1081     CCTCCACAACCTGAGAACCTTCCGGTGCCAGAAGCGCTGGATTTTATGAACGAGAAGAAT  1140
                ||||||||||||||||||||||||||||||||||||||||||||||||||||||||||||
Sbjct  1184710  CCTCCACAACCTGAGAACCTTCCGGTGCCAGAAGCGCTGGATTTTATGAACGAGAAGAAT  1184769

Query  1141     ACTGATGGCTAA  1152
                ||||||||||||
Sbjct  1184770  ACTGATGGCTAA  1184781
```

Salmonella enterica subsp. enterica serovar Enteritidis str. EC20120685 genome

Sequence ID: **gb|CP007339.2|** Length: 4685833 Number of Matches: 1

Range 1: 1183629 to 1184780

**Score**	**Expect**	**Identities**	**Gaps**	**Strand**	**Frame**
2128 bits(1152)	0.0()	1152/1152(100%)	0/1152(0%)	Plus/Plus	

Features:

```
Query  1        GTGGCAGAAGAGAGCGACGACGACAAAACAGAAGCCCCCACACCCCACCGACTTGAAAAA  60
                ||||||||||||||||||||||||||||||||||||||||||||||||||||||||||||
Sbjct  1183629  GTGGCAGAAGAGAGCGACGACGACAAAACAGAAGCCCCCACACCCCACCGACTTGAAAAA  1183688

Query  61       GCGCGGGAAGAAGGGCAGATCCCCCGTTCCAGAGAACTGACCTCACTGCTGATATTGCTG  120
                ||||||||||||||||||||||||||||||||||||||||||||||||||||||||||||
Sbjct  1183689  GCGCGGGAAGAAGGGCAGATCCCCCGTTCCAGAGAACTGACCTCACTGCTGATATTGCTG  1183748

Query  121      GTGGGCGTTTGTATTATTTGGTTCGGCGGCGAGTCGTTAGCGCGGCAACTGGCGGGAATG  180
                ||||||||||||||||||||||||||||||||||||||||||||||||||||||||||||
Sbjct  1183749  GTGGGCGTTTGTATTATTTGGTTCGGCGGCGAGTCGTTAGCGCGGCAACTGGCGGGAATG  1183808

Query  181      CTCTCAGCAGGCCTGCACTTCGATCACCGTATGGTGAACGATCCTAACCTGATCCTGGGG  240
                ||||||||||||||||||||||||||||||||||||||||||||||||||||||||||||
Sbjct  1183809  CTCTCAGCAGGCCTGCACTTCGATCACCGTATGGTGAACGATCCTAACCTGATCCTGGGG  1183868

Query  241      CAGATAATTTTGCTGATTAAAGCGGCGATGATGGCACTGCTACCGCTCATCGCGGGCGTG  300
                ||||||||||||||||||||||||||||||||||||||||||||||||||||||||||||
Sbjct  1183869  CAGATAATTTTGCTGATTAAAGCGGCGATGATGGCACTGCTACCGCTCATCGCGGGCGTG  1183928

Query  301      GTACTGGTGGCGCTTATCTCGCCGGTTATGCTTGGCGGCCTGATTTTTAGCGGTAAGTCG  360
                ||||||||||||||||||||||||||||||||||||||||||||||||||||||||||||
Sbjct  1183929  GTACTGGTGGCGCTTATCTCGCCGGTTATGCTTGGCGGCCTGATTTTTAGCGGTAAGTCG  1183988

Query  361      CTACAGCCAAAATTTTCTAAATTAAACCCGCTGCCGGGAATTAAGCGCATGTTTTCGGCG  420
                ||||||||||||||||||||||||||||||||||||||||||||||||||||||||||||
Sbjct  1183989  CTACAGCCAAAATTTTCTAAATTAAACCCGCTGCCGGGAATTAAGCGCATGTTTTCGGCG  1184048

Query  421      CAGACCGGCGCGGAACTGCTAAAAGCGGTGTTGAAATCCACGCTGGTCGGCTGCGTTACC  480
                ||||||||||||||||||||||||||||||||||||||||||||||||||||||||||||
Sbjct  1184049  CAGACCGGCGCGGAACTGCTAAAAGCGGTGTTGAAATCCACGCTGGTCGGCTGCGTTACC  1184108

Query  481      GGCTTTTATCTCTGGTATCACTGGCCACAAATGATGCGCCTGATGGCGGAGTCGCCGATC  540
                ||||||||||||||||||||||||||||||||||||||||||||||||||||||||||||
```

```
Sbjct  1184109  GGCTTTTATCTCTGGTATCACTGGCCACAAATGATGCGCCTGATGGCGGAGTCGCCGATC  1184168

Query  541      GTCGCAATGGGGAATGCGCTGGATCTGGTTGGACTCTGCGCGTTACTGGTGGTACTGGGC  600
                ||||||||||||||||||||||||||||||||||||||||||||||||||||||||||||
Sbjct  1184169  GTCGCAATGGGGAATGCGCTGGATCTGGTTGGACTCTGCGCGTTACTGGTGGTACTGGGC  1184228

Query  601      GTGATTCCGATGGTGGGATTTGACGTGTTTTTCCAGATCTTTAGCCACCTGAAAAAATTA  660
                ||||||||||||||||||||||||||||||||||||||||||||||||||||||||||||
Sbjct  1184229  GTGATTCCGATGGTGGGATTTGACGTGTTTTTCCAGATCTTTAGCCACCTGAAAAAATTA  1184288

Query  661      CGCATGTCGCGGCAGGACATTCGCGACGAATTTAAAGAGAGCGAAGGCGATCCGCATGTT  720
                ||||||||||||||||||||||||||||||||||||||||||||||||||||||||||||
Sbjct  1184289  CGCATGTCGCGGCAGGACATTCGCGACGAATTTAAAGAGAGCGAAGGCGATCCGCATGTT  1184348

Query  721      AAGGGCAAAATTCGCCAGATGCAACGCGCCGCCGCGCAGCGCCGCATGATGGAAGATGTG  780
                ||||||||||||||||||||||||||||||||||||||||||||||||||||||||||||
Sbjct  1184349  AAGGGCAAAATTCGCCAGATGCAACGCGCCGCCGCGCAGCGCCGCATGATGGAAGATGTG  1184408

Query  781      CCGAAAGCGGACGTCATTGTCACTAACCCGACGCACTATTCCGTGGCGCTGCAGTATGAC  840
                ||||||||||||||||||||||||||||||||||||||||||||||||||||||||||||
Sbjct  1184409  CCGAAAGCGGACGTCATTGTCACTAACCCGACGCACTATTCCGTGGCGCTGCAGTATGAC  1184468

Query  841      GAAAACAAAATGAGCGCGCCGAAAGTGGTCGCGAAGGGGGCTGGATTAATAGCGCTGCGC  900
                ||||||||||||||||||||||||||||||||||||||||||||||||||||||||||||
Sbjct  1184469  GAAAACAAAATGAGCGCGCCGAAAGTGGTCGCGAAGGGGGCTGGATTAATAGCGCTGCGC  1184528

Query  901      ATTCGCGAGATCGGCGCTGAACATCGGGTTCCCACTTTAGAAGCGCCGCCGCTGGCGCGG  960
                ||||||||||||||||||||||||||||||||||||||||||||||||||||||||||||
Sbjct  1184529  ATTCGCGAGATCGGCGCTGAACATCGGGTTCCCACTTTAGAAGCGCCGCCGCTGGCGCGG  1184588

Query  961      GCATTATATCGCCACGCCGAAATCGGTCAGCAAATTCCCGGGCAGTTATATGCTGCCGTT  1020
                ||||||||||||||||||||||||||||||||||||||||||||||||||||||||||||
Sbjct  1184589  GCATTATATCGCCACGCCGAAATCGGTCAGCAAATTCCCGGGCAGTTATATGCTGCCGTT  1184648

Query  1021     GCGGAAGTGTTGGCCTGGGTCTGGCAGCTTAAACGCTGGCGGCTTGCGGGCGGGCAACGT  1080
                ||||||||||||||||||||||||||||||||||||||||||||||||||||||||||||
Sbjct  1184649  GCGGAAGTGTTGGCCTGGGTCTGGCAGCTTAAACGCTGGCGGCTTGCGGGCGGGCAACGT  1184708

Query  1081     CCTCCACAACCTGAGAACCTTCCGGTGCCAGAAGCGCTGGATTTTATGAACGAGAAGAAT  1140
                ||||||||||||||||||||||||||||||||||||||||||||||||||||||||||||
Sbjct  1184709  CCTCCACAACCTGAGAACCTTCCGGTGCCAGAAGCGCTGGATTTTATGAACGAGAAGAAT  1184768

Query  1141     ACTGATGGCTAA  1152
                ||||||||||||
Sbjct  1184769  ACTGATGGCTAA  1184780
```

Salmonella enterica subsp. enterica serovar Pullorum str. ATCC 9120, complete genome

Sequence ID: **gb|CP012347.1|** Length: 4694842 Number of Matches: 2

Range 1: 2686143 to 2686897

Score	Expect	Identities	Gaps	Strand	Frame
1395 bits(755)	0.0()	755/755(100%)	0/755(0%)	Plus/Minus	

Features:

```
Query  1        GTGGCAGAAGAGAGCGACGACGACAAAACAGAAGCCCCCACACCCCACCGACTTGAAAAA  60
                ||||||||||||||||||||||||||||||||||||||||||||||||||||||||||||
Sbjct  2686897  GTGGCAGAAGAGAGCGACGACGACAAAACAGAAGCCCCCACACCCCACCGACTTGAAAAA  2686838

Query  61       GCGCGGGAAGAAGGGCAGATCCCCCGTTCCAGAGAACTGACCTCACTGCTGATATTGCTG  120
                ||||||||||||||||||||||||||||||||||||||||||||||||||||||||||||
Sbjct  2686837  GCGCGGGAAGAAGGGCAGATCCCCCGTTCCAGAGAACTGACCTCACTGCTGATATTGCTG  2686778

Query  121      GTGGGCGTTTGTATTATTTGGTTCGGCGGCGAGTCGTTAGCGCGGCAACTGGCGGGAATG  180
                ||||||||||||||||||||||||||||||||||||||||||||||||||||||||||||
Sbjct  2686777  GTGGGCGTTTGTATTATTTGGTTCGGCGGCGAGTCGTTAGCGCGGCAACTGGCGGGAATG  2686718

Query  181      CTCTCAGCAGGCCTGCACTTCGATCACCGTATGGTGAACGATCCTAACCTGATCCTGGGG  240
                ||||||||||||||||||||||||||||||||||||||||||||||||||||||||||||
Sbjct  2686717  CTCTCAGCAGGCCTGCACTTCGATCACCGTATGGTGAACGATCCTAACCTGATCCTGGGG  2686658

Query  241      CAGATAATTTTGCTGATTAAAGCGGCGATGATGGCACTGCTACCGCTCATCGCGGGCGTG  300
                ||||||||||||||||||||||||||||||||||||||||||||||||||||||||||||
Sbjct  2686657  CAGATAATTTTGCTGATTAAAGCGGCGATGATGGCACTGCTACCGCTCATCGCGGGCGTG  2686598

Query  301      GTACTGGTGGCGCTTATCTCGCCGGTTATGCTTGGCGGCCTGATTTTTAGCGGTAAGTCG  360
                ||||||||||||||||||||||||||||||||||||||||||||||||||||||||||||
Sbjct  2686597  GTACTGGTGGCGCTTATCTCGCCGGTTATGCTTGGCGGCCTGATTTTTAGCGGTAAGTCG  2686538

Query  361      CTACAGCCAAAATTTTCTAAATTAAACCCGCTGCCGGGAATTAAGCGCATGTTTTCGGCG  420
                ||||||||||||||||||||||||||||||||||||||||||||||||||||||||||||
Sbjct  2686537  CTACAGCCAAAATTTTCTAAATTAAACCCGCTGCCGGGAATTAAGCGCATGTTTTCGGCG  2686478

Query  421      CAGACCGGCGCGGAACTGCTAAAAGCGGTGTTGAAATCCACGCTGGTCGGCTGCGTTACC  480
                ||||||||||||||||||||||||||||||||||||||||||||||||||||||||||||
Sbjct  2686477  CAGACCGGCGCGGAACTGCTAAAAGCGGTGTTGAAATCCACGCTGGTCGGCTGCGTTACC  2686418

Query  481      GGCTTTTATCTCTGGTATCACTGGCCACAAATGATGCGCCTGATGGCGGAGTCGCCGATC  540
                ||||||||||||||||||||||||||||||||||||||||||||||||||||||||||||
Sbjct  2686417  GGCTTTTATCTCTGGTATCACTGGCCACAAATGATGCGCCTGATGGCGGAGTCGCCGATC  2686358

Query  541      GTCGCAATGGGGAATGCGCTGGATCTGGTTGGACTCTGCGCGTTACTGGTGGTACTGGGC  600
                ||||||||||||||||||||||||||||||||||||||||||||||||||||||||||||
Sbjct  2686357  GTCGCAATGGGGAATGCGCTGGATCTGGTTGGACTCTGCGCGTTACTGGTGGTACTGGGC  2686298

Query  601      GTGATTCCGATGGTGGGATTTGACGTGTTTTTCCAGATCTTTAGCCACCTGAAAAAATTA  660
                ||||||||||||||||||||||||||||||||||||||||||||||||||||||||||||
Sbjct  2686297  GTGATTCCGATGGTGGGATTTGACGTGTTTTTCCAGATCTTTAGCCACCTGAAAAAATTA  2686238

Query  661      CGCATGTCGCGGCAGGACATTCGCGACGAATTTAAAGAGAGCGAAGGCGATCCGCATGTT  720
                ||||||||||||||||||||||||||||||||||||||||||||||||||||||||||||
Sbjct  2686237  CGCATGTCGCGGCAGGACATTCGCGACGAATTTAAAGAGAGCGAAGGCGATCCGCATGTT  2686178

Query  721      AAGGGCAAAATTCGCCAGATGCAACGCGCCGCCGC  755
```

```
             |||||||||||||||||||||||||||||||||||
Sbjct  2686177  AAGGGCAAAATTCGCCAGATGCAACGCGCCGCCGC  2686143
```

Range 2: 2685943 to 2686152

Score	Expect	Identities	Gaps	Strand	Frame
388 bits(210)	1e-103()	210/210(100%)	0/210(0%)	Plus/Minus	

Features:

```
Query  943      GCGCCGCCGCTGGCGCGGGCATTATATCGCCACGCCGAAATCGGTCAGCAAATTCCCGGG  1002
                ||||||||||||||||||||||||||||||||||||||||||||||||||||||||||||
Sbjct  2686152  GCGCCGCCGCTGGCGCGGGCATTATATCGCCACGCCGAAATCGGTCAGCAAATTCCCGGG  2686093

Query  1003     CAGTTATATGCTGCCGTTGCGGAAGTGTTGGCCTGGGTCTGGCAGCTTAAACGCTGGCGG  1062
                ||||||||||||||||||||||||||||||||||||||||||||||||||||||||||||
Sbjct  2686092  CAGTTATATGCTGCCGTTGCGGAAGTGTTGGCCTGGGTCTGGCAGCTTAAACGCTGGCGG  2686033

Query  1063     CTTGCGGGCGGGCAACGTCCTCCACAACCTGAGAACCTTCCGGTGCCAGAAGCGCTGGAT  1122
                ||||||||||||||||||||||||||||||||||||||||||||||||||||||||||||
Sbjct  2686032  CTTGCGGGCGGGCAACGTCCTCCACAACCTGAGAACCTTCCGGTGCCAGAAGCGCTGGAT  2685973

Query  1123     TTTATGAACGAGAAGAATACTGATGGCTAA  1152
                ||||||||||||||||||||||||||||||
Sbjct  2685972  TTTATGAACGAGAAGAATACTGATGGCTAA  2685943
```

Salmonella enterica subsp. enterica serovar Pullorum genome assembly S44987_1 ,chromosome : I
Sequence ID: **emb|LK931482.1|** Length: 4620579 Number of Matches: 2
Range 1: 1144166 to 1144920

Score	Expect	Identities	Gaps	Strand	Frame
1395 bits(755)	0.0()	755/755(100%)	0/755(0%)	Plus/Plus	

Features:

```
Query  1        GTGGCAGAAGAGAGCGACGACGACAAAACAGAAGCCCCCACACCCCACCGACTTGAAAAA  60
                ||||||||||||||||||||||||||||||||||||||||||||||||||||||||||||
Sbjct  1144166  GTGGCAGAAGAGAGCGACGACGACAAAACAGAAGCCCCCACACCCCACCGACTTGAAAAA  1144225

Query  61       GCGCGGGAAGAAGGGCAGATCCCCCGTTCCAGAGAACTGACCTCACTGCTGATATTGCTG  120
                ||||||||||||||||||||||||||||||||||||||||||||||||||||||||||||
Sbjct  1144226  GCGCGGGAAGAAGGGCAGATCCCCCGTTCCAGAGAACTGACCTCACTGCTGATATTGCTG  1144285

Query  121      GTGGGCGTTTGTATTATTTGGTTCGGCGGCGAGTCGTTAGCGCGGCAACTGGCGGGAATG  180
                ||||||||||||||||||||||||||||||||||||||||||||||||||||||||||||
Sbjct  1144286  GTGGGCGTTTGTATTATTTGGTTCGGCGGCGAGTCGTTAGCGCGGCAACTGGCGGGAATG  1144345

Query  181      CTCTCAGCAGGCCTGCACTTCGATCACCGTATGGTGAACGATCCTAACCTGATCCTGGGG  240
                ||||||||||||||||||||||||||||||||||||||||||||||||||||||||||||
Sbjct  1144346  CTCTCAGCAGGCCTGCACTTCGATCACCGTATGGTGAACGATCCTAACCTGATCCTGGGG  1144405

Query  241      CAGATAATTTTGCTGATTAAAGCGGCGATGATGGCACTGCTACCGCTCATCGCGGGCGTG  300
                ||||||||||||||||||||||||||||||||||||||||||||||||||||||||||||
Sbjct  1144406  CAGATAATTTTGCTGATTAAAGCGGCGATGATGGCACTGCTACCGCTCATCGCGGGCGTG  1144465

Query  301      GTACTGGTGGCGCTTATCTCGCCGGTTATGCTTGGCGGCCTGATTTTTAGCGGTAAGTCG  360
                ||||||||||||||||||||||||||||||||||||||||||||||||||||||||||||
Sbjct  1144466  GTACTGGTGGCGCTTATCTCGCCGGTTATGCTTGGCGGCCTGATTTTTAGCGGTAAGTCG  1144525

Query  361      CTACAGCCAAAATTTTCTAAATTAAACCCGCTGCCGGGAATTAAGCGCATGTTTTCGGCG  420
                ||||||||||||||||||||||||||||||||||||||||||||||||||||||||||||
Sbjct  1144526  CTACAGCCAAAATTTTCTAAATTAAACCCGCTGCCGGGAATTAAGCGCATGTTTTCGGCG  1144585

Query  421      CAGACCGGCGCGGAACTGCTAAAAGCGGTGTTGAAATCCACGCTGGTCGGCTGCGTTACC  480
                ||||||||||||||||||||||||||||||||||||||||||||||||||||||||||||
Sbjct  1144586  CAGACCGGCGCGGAACTGCTAAAAGCGGTGTTGAAATCCACGCTGGTCGGCTGCGTTACC  1144645

Query  481      GGCTTTTATCTCTGGTATCACTGGCCACAAATGATGCGCCTGATGGCGGAGTCGCCGATC  540
                ||||||||||||||||||||||||||||||||||||||||||||||||||||||||||||
Sbjct  1144646  GGCTTTTATCTCTGGTATCACTGGCCACAAATGATGCGCCTGATGGCGGAGTCGCCGATC  1144705

Query  541      GTCGCAATGGGGAATGCGCTGGATCTGGTTGGACTCTGCGCGTTACTGGTGGTACTGGGC  600
                ||||||||||||||||||||||||||||||||||||||||||||||||||||||||||||
Sbjct  1144706  GTCGCAATGGGGAATGCGCTGGATCTGGTTGGACTCTGCGCGTTACTGGTGGTACTGGGC  1144765

Query  601      GTGATTCCGATGGTGGGATTTGACGTGTTTTTCCAGATCTTTAGCCACCTGAAAAAAATTA  660
                ||||||||||||||||||||||||||||||||||||||||||||||||||||||||||||
Sbjct  1144766  GTGATTCCGATGGTGGGATTTGACGTGTTTTTCCAGATCTTTAGCCACCTGAAAAAAATTA  1144825

Query  661      CGCATGTCGCGGCAGGACATTCGCGACGAATTTAAAGAGAGCGAAGGCGATCCGCATGTT  720
                ||||||||||||||||||||||||||||||||||||||||||||||||||||||||||||
Sbjct  1144826  CGCATGTCGCGGCAGGACATTCGCGACGAATTTAAAGAGAGCGAAGGCGATCCGCATGTT  1144885

Query  721      AAGGGCAAAATTCGCCAGATGCAACGCGCCGCCGC  755
                |||||||||||||||||||||||||||||||||||
Sbjct  1144886  AAGGGCAAAATTCGCCAGATGCAACGCGCCGCCGC  1144920
```

Range 2: 1144911 to 1145120

Score	Expect	Identities	Gaps	Strand	Frame
388 bits(210)	1e-103()	210/210(100%)	0/210(0%)	Plus/Plus	

Features:

```
Query  943      GCGCCGCCGCTGGCGCGGGCATTATATCGCCACGCCGAAATCGGTCAGCAAATTCCCGGG  1002
```

```
                ||||||||||||||||||||||||||||||||||||||||||||||||||||||||||||
Sbjct  1144911  GCGCCGCCGCTGGCGCGGGCATTATATCGCCACGCCGAAATCGGTCAGCAAATTCCCGGG  1144970
Query  1003     CAGTTATATGCTGCCGTTGCGGAAGTGTTGGCCTGGGTCTGGCAGCTTAAACGCTGGCGG  1062
                ||||||||||||||||||||||||||||||||||||||||||||||||||||||||||||
Sbjct  1144971  CAGTTATATGCTGCCGTTGCGGAAGTGTTGGCCTGGGTCTGGCAGCTTAAACGCTGGCGG  1145030
Query  1063     CTTGCGGGCGGGCAACGTCCTCCACAACCTGAGAACCTTCCGGTGCCAGAAGCGCTGGAT  1122
                ||||||||||||||||||||||||||||||||||||||||||||||||||||||||||||
Sbjct  1145031  CTTGCGGGCGGGCAACGTCCTCCACAACCTGAGAACCTTCCGGTGCCAGAAGCGCTGGAT  1145090
Query  1123     TTTATGAACGAGAAGAATACTGATGGCTAA  1152
                ||||||||||||||||||||||||||||||
Sbjct  1145091  TTTATGAACGAGAAGAATACTGATGGCTAA  1145120
```

Salmonella enterica subsp. enterica serovar Gallinarum/pullorum str. CDC1983-67, complete genome
Sequence ID: **gb|CP003786.1|** Length: 4623089 Number of Matches: 2
Range 1: 1796153 to 1796907

Score	Expect	Identities	Gaps	Strand	Frame
1395 bits(755)	0.0()	755/755(100%)	0/755(0%)	Plus/Minus	

Features:

```
Query  1        GTGGCAGAAGAGAGCGACGACGACAAAACAGAAGCCCCCACACCCCACCGACTTGAAAAA  60
                ||||||||||||||||||||||||||||||||||||||||||||||||||||||||||||
Sbjct  1796907  GTGGCAGAAGAGAGCGACGACGACAAAACAGAAGCCCCCACACCCCACCGACTTGAAAAA  1796848
Query  61       GCGCGGGAAGAAGGGCAGATCCCCCGTTCCAGAGAACTGACCTCACTGCTGATATTGCTG  120
                ||||||||||||||||||||||||||||||||||||||||||||||||||||||||||||
Sbjct  1796847  GCGCGGGAAGAAGGGCAGATCCCCCGTTCCAGAGAACTGACCTCACTGCTGATATTGCTG  1796788
Query  121      GTGGGCGTTTGTATTATTTGGTTCGGCGGCGAGTCGTTAGCGCGGCAACTGGCGGGAATG  180
                ||||||||||||||||||||||||||||||||||||||||||||||||||||||||||||
Sbjct  1796787  GTGGGCGTTTGTATTATTTGGTTCGGCGGCGAGTCGTTAGCGCGGCAACTGGCGGGAATG  1796728
Query  181      CTCTCAGCAGGCCTGCACTTCGATCACCGTATGGTGAACGATCCTAACCTGATCCTGGGG  240
                ||||||||||||||||||||||||||||||||||||||||||||||||||||||||||||
Sbjct  1796727  CTCTCAGCAGGCCTGCACTTCGATCACCGTATGGTGAACGATCCTAACCTGATCCTGGGG  1796668
Query  241      CAGATAATTTTGCTGATTAAAGCGGCGATGATGGCACTGCTACCGCTCATCGCGGGCGTG  300
                ||||||||||||||||||||||||||||||||||||||||||||||||||||||||||||
Sbjct  1796667  CAGATAATTTTGCTGATTAAAGCGGCGATGATGGCACTGCTACCGCTCATCGCGGGCGTG  1796608
Query  301      GTACTGGTGGCGCTTATCTCGCCGGTTATGCTTGGCGGCCTGATTTTTAGCGGTAAGTCG  360
                ||||||||||||||||||||||||||||||||||||||||||||||||||||||||||||
Sbjct  1796607  GTACTGGTGGCGCTTATCTCGCCGGTTATGCTTGGCGGCCTGATTTTTAGCGGTAAGTCG  1796548
Query  361      CTACAGCCAAAATTTTCTAAATTAAACCCGCTGCCGGGAATTAAGCGCATGTTTTCGGCG  420
                ||||||||||||||||||||||||||||||||||||||||||||||||||||||||||||
Sbjct  1796547  CTACAGCCAAAATTTTCTAAATTAAACCCGCTGCCGGGAATTAAGCGCATGTTTTCGGCG  1796488
Query  421      CAGACCGGCGCGGAACTGCTAAAAGCGGTGTTGAAATCCACGCTGGTCGGCTGCGTTACC  480
                ||||||||||||||||||||||||||||||||||||||||||||||||||||||||||||
Sbjct  1796487  CAGACCGGCGCGGAACTGCTAAAAGCGGTGTTGAAATCCACGCTGGTCGGCTGCGTTACC  1796428
Query  481      GGCTTTTATCTCTGGTATCACTGGCCACAAATGATGCGCCTGATGGCGGAGTCGCCGATC  540
                ||||||||||||||||||||||||||||||||||||||||||||||||||||||||||||
Sbjct  1796427  GGCTTTTATCTCTGGTATCACTGGCCACAAATGATGCGCCTGATGGCGGAGTCGCCGATC  1796368
Query  541      GTCGCAATGGGGAATGCGCTGGATCTGGTTGGACTCTGCGCGTTACTGGTGGTACTGGGC  600
                ||||||||||||||||||||||||||||||||||||||||||||||||||||||||||||
Sbjct  1796367  GTCGCAATGGGGAATGCGCTGGATCTGGTTGGACTCTGCGCGTTACTGGTGGTACTGGGC  1796308
Query  601      GTGATTCCGATGGTGGGATTTGACGTGTTTTTCCAGATCTTTAGCCACCTGAAAAAATTA  660
                ||||||||||||||||||||||||||||||||||||||||||||||||||||||||||||
Sbjct  1796307  GTGATTCCGATGGTGGGATTTGACGTGTTTTTCCAGATCTTTAGCCACCTGAAAAAATTA  1796248
Query  661      CGCATGTCGCGGCAGGACATTCGCGACGAATTTAAAGAGAGCGAAGGCGATCCGCATGTT  720
                ||||||||||||||||||||||||||||||||||||||||||||||||||||||||||||
Sbjct  1796247  CGCATGTCGCGGCAGGACATTCGCGACGAATTTAAAGAGAGCGAAGGCGATCCGCATGTT  1796188
Query  721      AAGGGCAAAATTCGCCAGATGCAACGCGCCGCCGC  755
                |||||||||||||||||||||||||||||||||||
Sbjct  1796187  AAGGGCAAAATTCGCCAGATGCAACGCGCCGCCGC  1796153
```

Range 2: 1795953 to 1796162

Score	Expect	Identities	Gaps	Strand	Frame
388 bits(210)	1e-103()	210/210(100%)	0/210(0%)	Plus/Minus	

Features:

```
Query  943      GCGCCGCCGCTGGCGCGGGCATTATATCGCCACGCCGAAATCGGTCAGCAAATTCCCGGG  1002
                ||||||||||||||||||||||||||||||||||||||||||||||||||||||||||||
Sbjct  1796162  GCGCCGCCGCTGGCGCGGGCATTATATCGCCACGCCGAAATCGGTCAGCAAATTCCCGGG  1796103
Query  1003     CAGTTATATGCTGCCGTTGCGGAAGTGTTGGCCTGGGTCTGGCAGCTTAAACGCTGGCGG  1062
                ||||||||||||||||||||||||||||||||||||||||||||||||||||||||||||
Sbjct  1796102  CAGTTATATGCTGCCGTTGCGGAAGTGTTGGCCTGGGTCTGGCAGCTTAAACGCTGGCGG  1796043
Query  1063     CTTGCGGGCGGGCAACGTCCTCCACAACCTGAGAACCTTCCGGTGCCAGAAGCGCTGGAT  1122
                ||||||||||||||||||||||||||||||||||||||||||||||||||||||||||||
Sbjct  1796042  CTTGCGGGCGGGCAACGTCCTCCACAACCTGAGAACCTTCCGGTGCCAGAAGCGCTGGAT  1795983
Query  1123     TTTATGAACGAGAAGAATACTGATGGCTAA  1152
                ||||||||||||||||||||||||||||||
Sbjct  1795982  TTTATGAACGAGAAGAATACTGATGGCTAA  1795953
```

Salmonella enterica subsp. enterica serovar Pullorum str. S06004, complete genome
Sequence ID: **gb|CP006575.1|** Length: 4682599 Number of Matches: 2
Range 1: 1769012 to 1769766

Score	Expect	Identities	Gaps	Strand	Frame
1395 bits(755)	0.0()	755/755(100%)	0/755(0%)	Plus/Minus	

Features:

```
Query  1        GTGGCAGAAGAGAGCGACGACGACAAAACAGAAGCCCCCACACCCCACCGACTTGAAAAA  60
                ||||||||||||||||||||||||||||||||||||||||||||||||||||||||||||
Sbjct  1769766  GTGGCAGAAGAGAGCGACGACGACAAAACAGAAGCCCCCACACCCCACCGACTTGAAAAA  1769707

Query  61       GCGCGGGAAGAAGGGCAGATCCCCCGTTCCAGAGAACTGACCTCACTGCTGATATTGCTG  120
                ||||||||||||||||||||||||||||||||||||||||||||||||||||||||||||
Sbjct  1769706  GCGCGGGAAGAAGGGCAGATCCCCCGTTCCAGAGAACTGACCTCACTGCTGATATTGCTG  1769647

Query  121      GTGGGCGTTTGTATTATTTGGTTCGGCGGCGAGTCGTTAGCGCGGCAACTGGCGGGAATG  180
                ||||||||||||||||||||||||||||||||||||||||||||||||||||||||||||
Sbjct  1769646  GTGGGCGTTTGTATTATTTGGTTCGGCGGCGAGTCGTTAGCGCGGCAACTGGCGGGAATG  1769587

Query  181      CTCTCAGCAGGCCTGCACTTCGATCACCGTATGGTGAACGATCCTAACCTGATCCTGGGG  240
                ||||||||||||||||||||||||||||||||||||||||||||||||||||||||||||
Sbjct  1769586  CTCTCAGCAGGCCTGCACTTCGATCACCGTATGGTGAACGATCCTAACCTGATCCTGGGG  1769527

Query  241      CAGATAATTTTGCTGATTAAAGCGGCGATGATGGCACTGCTACCGCTCATCGCGGGCGTG  300
                ||||||||||||||||||||||||||||||||||||||||||||||||||||||||||||
Sbjct  1769526  CAGATAATTTTGCTGATTAAAGCGGCGATGATGGCACTGCTACCGCTCATCGCGGGCGTG  1769467

Query  301      GTACTGGTGGCGCTTATCTCGCCGGTTATGCTTGGCGGCCTGATTTTTAGCGGTAAGTCG  360
                ||||||||||||||||||||||||||||||||||||||||||||||||||||||||||||
Sbjct  1769466  GTACTGGTGGCGCTTATCTCGCCGGTTATGCTTGGCGGCCTGATTTTTAGCGGTAAGTCG  1769407

Query  361      CTACAGCCAAAATTTTCTAAATTAAACCCGCTGCCGGGAATTAAGCGCATGTTTTCGGCG  420
                ||||||||||||||||||||||||||||||||||||||||||||||||||||||||||||
Sbjct  1769406  CTACAGCCAAAATTTTCTAAATTAAACCCGCTGCCGGGAATTAAGCGCATGTTTTCGGCG  1769347

Query  421      CAGACCGGCGCGGAACTGCTAAAAGCGGTGTTGAAATCCACGCTGGTCGGCTGCGTTACC  480
                ||||||||||||||||||||||||||||||||||||||||||||||||||||||||||||
Sbjct  1769346  CAGACCGGCGCGGAACTGCTAAAAGCGGTGTTGAAATCCACGCTGGTCGGCTGCGTTACC  1769287

Query  481      GGCTTTTATCTCTGGTATCACTGGCCACAAATGATGCGCCTGATGGCGGAGTCGCCGATC  540
                ||||||||||||||||||||||||||||||||||||||||||||||||||||||||||||
Sbjct  1769286  GGCTTTTATCTCTGGTATCACTGGCCACAAATGATGCGCCTGATGGCGGAGTCGCCGATC  1769227

Query  541      GTCGCAATGGGGAATGCGCTGGATCTGGTTGGACTCTGCGCGTTACTGGTGGTACTGGGC  600
                ||||||||||||||||||||||||||||||||||||||||||||||||||||||||||||
Sbjct  1769226  GTCGCAATGGGGAATGCGCTGGATCTGGTTGGACTCTGCGCGTTACTGGTGGTACTGGGC  1769167

Query  601      GTGATTCCGATGGTGGGATTTGACGTGTTTTTCCAGATCTTTAGCCACCTGAAAAAATTA  660
                ||||||||||||||||||||||||||||||||||||||||||||||||||||||||||||
Sbjct  1769166  GTGATTCCGATGGTGGGATTTGACGTGTTTTTCCAGATCTTTAGCCACCTGAAAAAATTA  1769107

Query  661      CGCATGTCGCGGCAGGACATTCGCGACGAATTTAAAGAGAGCGAAGGCGATCCGCATGTT  720
                ||||||||||||||||||||||||||||||||||||||||||||||||||||||||||||
Sbjct  1769106  CGCATGTCGCGGCAGGACATTCGCGACGAATTTAAAGAGAGCGAAGGCGATCCGCATGTT  1769047

Query  721      AAGGGCAAAATTCGCCAGATGCAACGCGCCGCCGC  755
                |||||||||||||||||||||||||||||||||||
Sbjct  1769046  AAGGGCAAAATTCGCCAGATGCAACGCGCCGCCGC  1769012
```

Range 2: 1768812 to 1769021

Score	Expect	Identities	Gaps	Strand	Frame
388 bits(210)	1e-103()	210/210(100%)	0/210(0%)	Plus/Minus	

Features:

```
Query  943      GCGCCGCCGCTGGCGCGGGCATTATATCGCCACGCCGAAATCGGTCAGCAAATTCCCGGG  1002
                ||||||||||||||||||||||||||||||||||||||||||||||||||||||||||||
Sbjct  1769021  GCGCCGCCGCTGGCGCGGGCATTATATCGCCACGCCGAAATCGGTCAGCAAATTCCCGGG  1768962

Query  1003     CAGTTATATGCTGCCGTTGCGGAAGTGTTGGCCTGGGTCTGGCAGCTTAAACGCTGGCGG  1062
                ||||||||||||||||||||||||||||||||||||||||||||||||||||||||||||
Sbjct  1768961  CAGTTATATGCTGCCGTTGCGGAAGTGTTGGCCTGGGTCTGGCAGCTTAAACGCTGGCGG  1768902

Query  1063     CTTGCGGGCGGGCAACGTCCTCCACAACCTGAGAACCTTCCGGTGCCAGAAGCGCTGGAT  1122
                ||||||||||||||||||||||||||||||||||||||||||||||||||||||||||||
Sbjct  1768901  CTTGCGGGCGGGCAACGTCCTCCACAACCTGAGAACCTTCCGGTGCCAGAAGCGCTGGAT  1768842

Query  1123     TTTATGAACGAGAAGAATACTGATGGCTAA  1152
                ||||||||||||||||||||||||||||||
Sbjct  1768841  TTTATGAACGAGAAGAATACTGATGGCTAA  1768812
```

Salmonella enterica subsp. enterica serovar Gallinarum/pullorum str. RKS5078, complete genome
Sequence ID: **gb|CP003047.1|** Length: 4637962 Number of Matches: 2
Range 1: 1804085 to 1804839

Score	Expect	Identities	Gaps	Strand	Frame
1395 bits(755)	0.0()	755/755(100%)	0/755(0%)	Plus/Minus	

Features:

```
Query  1        GTGGCAGAAGAGAGCGACGACGACAAAACAGAAGCCCCCACACCCCACCGACTTGAAAAA  60
                ||||||||||||||||||||||||||||||||||||||||||||||||||||||||||||
```

```
Sbjct  1804839  GTGGCAGAAGAGAGCGACGACGACAAAACAGAAGCCCCCACACCCCACCGACTTGAAAAA  1804780
Query  61       GCGCGGGAAGAAGGGCAGATCCCCCGTTCCAGAGAACTGACCTCACTGCTGATATTGCTG  120
                ||||||||||||||||||||||||||||||||||||||||||||||||||||||||||||
Sbjct  1804779  GCGCGGGAAGAAGGGCAGATCCCCCGTTCCAGAGAACTGACCTCACTGCTGATATTGCTG  1804720
Query  121      GTGGGCGTTTGTATTATTTGGTTCGGCGGCGAGTCGTTAGCGCGGCAACTGGCGGGAATG  180
                ||||||||||||||||||||||||||||||||||||||||||||||||||||||||||||
Sbjct  1804719  GTGGGCGTTTGTATTATTTGGTTCGGCGGCGAGTCGTTAGCGCGGCAACTGGCGGGAATG  1804660
Query  181      CTCTCAGCAGGCCTGCACTTCGATCACCGTATGGTGAACGATCCTAACCTGATCCTGGGG  240
                ||||||||||||||||||||||||||||||||||||||||||||||||||||||||||||
Sbjct  1804659  CTCTCAGCAGGCCTGCACTTCGATCACCGTATGGTGAACGATCCTAACCTGATCCTGGGG  1804600
Query  241      CAGATAATTTTGCTGATTAAAGCGGCGATGATGGCACTGCTACCGCTCATCGCGGGCGTG  300
                ||||||||||||||||||||||||||||||||||||||||||||||||||||||||||||
Sbjct  1804599  CAGATAATTTTGCTGATTAAAGCGGCGATGATGGCACTGCTACCGCTCATCGCGGGCGTG  1804540
Query  301      GTACTGGTGGCGCTTATCTCGCCGGTTATGCTTGGCGGCCTGATTTTTAGCGGTAAGTCG  360
                ||||||||||||||||||||||||||||||||||||||||||||||||||||||||||||
Sbjct  1804539  GTACTGGTGGCGCTTATCTCGCCGGTTATGCTTGGCGGCCTGATTTTTAGCGGTAAGTCG  1804480
Query  361      CTACAGCCAAAATTTTCTAAATTAAACCCGCTGCCGGGAATTAAGCGCATGTTTTCGGCG  420
                ||||||||||||||||||||||||||||||||||||||||||||||||||||||||||||
Sbjct  1804479  CTACAGCCAAAATTTTCTAAATTAAACCCGCTGCCGGGAATTAAGCGCATGTTTTCGGCG  1804420
Query  421      CAGACCGGCGCGGAACTGCTAAAAGCGGTGTTGAAATCCACGCTGGTCGGCTGCGTTACC  480
                ||||||||||||||||||||||||||||||||||||||||||||||||||||||||||||
Sbjct  1804419  CAGACCGGCGCGGAACTGCTAAAAGCGGTGTTGAAATCCACGCTGGTCGGCTGCGTTACC  1804360
Query  481      GGCTTTTATCTCTGGTATCACTGGCCACAAATGATGCGCCTGATGGCGGAGTCGCCGATC  540
                ||||||||||||||||||||||||||||||||||||||||||||||||||||||||||||
Sbjct  1804359  GGCTTTTATCTCTGGTATCACTGGCCACAAATGATGCGCCTGATGGCGGAGTCGCCGATC  1804300
Query  541      GTCGCAATGGGGAATGCGCTGGATCTGGTTGGACTCTGCGCGTTACTGGTGGTACTGGGC  600
                ||||||||||||||||||||||||||||||||||||||||||||||||||||||||||||
Sbjct  1804299  GTCGCAATGGGGAATGCGCTGGATCTGGTTGGACTCTGCGCGTTACTGGTGGTACTGGGC  1804240
Query  601      GTGATTCCGATGGTGGGATTTGACGTGTTTTTCCAGATCTTTAGCCACCTGAAAAAATTA  660
                ||||||||||||||||||||||||||||||||||||||||||||||||||||||||||||
Sbjct  1804239  GTGATTCCGATGGTGGGATTTGACGTGTTTTTCCAGATCTTTAGCCACCTGAAAAAATTA  1804180
Query  661      CGCATGTCGCGGCAGGACATTCGCGACGAATTTAAAGAGAGCGAAGGCGATCCGCATGTT  720
                ||||||||||||||||||||||||||||||||||||||||||||||||||||||||||||
Sbjct  1804179  CGCATGTCGCGGCAGGACATTCGCGACGAATTTAAAGAGAGCGAAGGCGATCCGCATGTT  1804120
Query  721      AAGGGCAAAATTCGCCAGATGCAACGCGCCGCCGC  755
                |||||||||||||||||||||||||||||||||||
Sbjct  1804119  AAGGGCAAAATTCGCCAGATGCAACGCGCCGCCGC  1804085
```

Range 2: 1803885 to 1804094

Score	Expect	Identities	Gaps	Strand	Frame
388 bits(210)	1e-103()	210/210(100%)	0/210(0%)	Plus/Minus	

Features:

```
Query  943      GCGCCGCCGCTGGCGCGGGCATTATATCGCCACGCCGAAATCGGTCAGCAAATTCCCGGG  1002
                ||||||||||||||||||||||||||||||||||||||||||||||||||||||||||||
Sbjct  1804094  GCGCCGCCGCTGGCGCGGGCATTATATCGCCACGCCGAAATCGGTCAGCAAATTCCCGGG  1804035
Query  1003     CAGTTATATGCTGCCGTTGCGGAAGTGTTGGCCTGGGTCTGGCAGCTTAAACGCTGGCGG  1062
                ||||||||||||||||||||||||||||||||||||||||||||||||||||||||||||
Sbjct  1804034  CAGTTATATGCTGCCGTTGCGGAAGTGTTGGCCTGGGTCTGGCAGCTTAAACGCTGGCGG  1803975
Query  1063     CTTGCGGGCGGGCAACGTCCTCCACAACCTGAGAACCTTCCGGTGCCAGAAGCGCTGGAT  1122
                ||||||||||||||||||||||||||||||||||||||||||||||||||||||||||||
Sbjct  1803974  CTTGCGGGCGGGCAACGTCCTCCACAACCTGAGAACCTTCCGGTGCCAGAAGCGCTGGAT  1803915
Query  1123     TTTATGAACGAGAAGAATACTGATGGCTAA  1152
                ||||||||||||||||||||||||||||||
Sbjct  1803914  TTTATGAACGAGAAGAATACTGATGGCTAA  1803885
```

Salmonella enterica subsp. enterica serovar Gallinarum str. 287/91 complete genome
Sequence ID: **emb|AM933173.1|** Length: 4658697 Number of Matches: 2
Range 1: 1220311 to 1221065

Score	Expect	Identities	Gaps	Strand	Frame
1395 bits(755)	0.0()	755/755(100%)	0/755(0%)	Plus/Plus	

Features:

```
Query  1        GTGGCAGAAGAGAGCGACGACGACAAAACAGAAGCCCCCACACCCCACCGACTTGAAAAA  60
                ||||||||||||||||||||||||||||||||||||||||||||||||||||||||||||
Sbjct  1220311  GTGGCAGAAGAGAGCGACGACGACAAAACAGAAGCCCCCACACCCCACCGACTTGAAAAA  1220370
Query  61       GCGCGGGAAGAAGGGCAGATCCCCCGTTCCAGAGAACTGACCTCACTGCTGATATTGCTG  120
                ||||||||||||||||||||||||||||||||||||||||||||||||||||||||||||
Sbjct  1220371  GCGCGGGAAGAAGGGCAGATCCCCCGTTCCAGAGAACTGACCTCACTGCTGATATTGCTG  1220430
Query  121      GTGGGCGTTTGTATTATTTGGTTCGGCGGCGAGTCGTTAGCGCGGCAACTGGCGGGAATG  180
                ||||||||||||||||||||||||||||||||||||||||||||||||||||||||||||
Sbjct  1220431  GTGGGCGTTTGTATTATTTGGTTCGGCGGCGAGTCGTTAGCGCGGCAACTGGCGGGAATG  1220490
Query  181      CTCTCAGCAGGCCTGCACTTCGATCACCGTATGGTGAACGATCCTAACCTGATCCTGGGG  240
                ||||||||||||||||||||||||||||||||||||||||||||||||||||||||||||
Sbjct  1220491  CTCTCAGCAGGCCTGCACTTCGATCACCGTATGGTGAACGATCCTAACCTGATCCTGGGG  1220550
Query  241      CAGATAATTTTGCTGATTAAAGCGGCGATGATGGCACTGCTACCGCTCATCGCGGGCGTG  300
                ||||||||||||||||||||||||||||||||||||||||||||||||||||||||||||
```

```
Sbjct  1220551  CAGATAATTTTGCTGATTAAAGCGGCGATGATGGCACTGCTACCGCTCATCGCGGGCGTG  1220610
Query  301      GTACTGGTGGCGCTTATCTCGCCGGTTATGCTTGGCGGCCTGATTTTTAGCGGTAAGTCG  360
Sbjct  1220611  GTACTGGTGGCGCTTATCTCGCCGGTTATGCTTGGCGGCCTGATTTTTAGCGGTAAGTCG  1220670
Query  361      CTACAGCCAAAATTTTCTAAATTAAACCCGCTGCCGGGAATTAAGCGCATGTTTTCGGCG  420
Sbjct  1220671  CTACAGCCAAAATTTTCTAAATTAAACCCGCTGCCGGGAATTAAGCGCATGTTTTCGGCG  1220730
Query  421      CAGACCGGCGCGGAACTGCTAAAAGCGGTGTTGAAATCCACGCTGGTCGGCTGCGTTACC  480
Sbjct  1220731  CAGACCGGCGCGGAACTGCTAAAAGCGGTGTTGAAATCCACGCTGGTCGGCTGCGTTACC  1220790
Query  481      GGCTTTTATCTCTGGTATCACTGGCCACAAATGATGCGCCTGATGGCGGAGTCGCCGATC  540
Sbjct  1220791  GGCTTTTATCTCTGGTATCACTGGCCACAAATGATGCGCCTGATGGCGGAGTCGCCGATC  1220850
Query  541      GTCGCAATGGGGAATGCGCTGGATCTGGTTGGACTCTGCGCGTTACTGGTGGTACTGGGC  600
Sbjct  1220851  GTCGCAATGGGGAATGCGCTGGATCTGGTTGGACTCTGCGCGTTACTGGTGGTACTGGGC  1220910
Query  601      GTGATTCCGATGGTGGGATTTGACGTGTTTTTCCAGATCTTTAGCCACCTGAAAAAATTA  660
Sbjct  1220911  GTGATTCCGATGGTGGGATTTGACGTGTTTTTCCAGATCTTTAGCCACCTGAAAAAATTA  1220970
Query  661      CGCATGTCGCGGCAGGACATTCGCGACGAATTTAAAGAGAGCGAAGGCGATCCGCATGTT  720
Sbjct  1220971  CGCATGTCGCGGCAGGACATTCGCGACGAATTTAAAGAGAGCGAAGGCGATCCGCATGTT  1221030
Query  721      AAGGGCAAAATTCGCCAGATGCAACGCGCCGCCGC  755
Sbjct  1221031  AAGGGCAAAATTCGCCAGATGCAACGCGCCGCCGC  1221065
```

Range 2: 1221056 to 1221265

Score	Expect	Identities	Gaps	Strand	Frame
388 bits(210)	1e-103()	210/210(100%)	0/210(0%)	Plus/Plus	

Features:

```
Query  943      GCGCCGCCGCTGGCGCGGGCATTATATCGCCACGCCGAAATCGGTCAGCAAATTCCCGGG  1002
Sbjct  1221056  GCGCCGCCGCTGGCGCGGGCATTATATCGCCACGCCGAAATCGGTCAGCAAATTCCCGGG  1221115
Query  1003     CAGTTATATGCTGCCGTTGCGGAAGTGTTGGCCTGGGTCTGGCAGCTTAAACGCTGGCGG  1062
Sbjct  1221116  CAGTTATATGCTGCCGTTGCGGAAGTGTTGGCCTGGGTCTGGCAGCTTAAACGCTGGCGG  1221175
Query  1063     CTTGCGGGCGGGCAACGTCCTCCACAACCTGAGAACCTTCCGGTGCCAGAAGCGCTGGAT  1122
Sbjct  1221176  CTTGCGGGCGGGCAACGTCCTCCACAACCTGAGAACCTTCCGGTGCCAGAAGCGCTGGAT  1221235
Query  1123     TTTATGAACGAGAAGAATACTGATGGCTAA  1152
Sbjct  1221236  TTTATGAACGAGAAGAATACTGATGGCTAA  1221265
```

S.typhimurium cheZ gene ,complete cds cheY gene, 3' end, and flaM gene (put.), 5' end
Sequence ID: **gb|M16691.1|STYCHEZ** Length: 1506 Number of Matches: 1
Range 1: 868 to 1506

Score	Expect	Identities	Gaps	Strand	Frame
1153 bits(624)	0.0()	634/639(99%)	0/639(0%)	Plus/Plus	

Features:

```
Query  1     GTGGCAGAAGAGAGCGACGACGACAAAACAGAAGCCCCCACACCCCACCGACTTGAAAAA  60
Sbjct  868   GTGGCAGAAGAGAGCGACGACGACAAAACAGAAGCCCCCACACCCCACCGACTTGAAAAA  927
Query  61    GCGCGGGAAGAAGGGCAGATCCCCCGTTCCAGAGAACTGACCTCACTGCTGATATTGCTG  120
Sbjct  928   GCGCGGGAAGAAGGGCAGATCCCCCGTTCCAGAGAACTGACCTCACTGCTGATATTGCTG  987
Query  121   GTGGGCGTTTGTATTATTTGGTTCGGCGGCGAGTCGTTAGCGCGGCAACTGGCGGGAATG  180
Sbjct  988   GTGGGCGTTTGTATTATTTGGTTCGGCGGCGAGTCGTTAGCGCGGCAGCTGGCGGGAATG  1047
Query  181   CTCTCAGCAGGCCTGCACTTCGATCACCGTATGGTGAACGATCCTAACCTGATCCTGGGG  240
Sbjct  1048  CTCTCAGCAGGCCTGCACTTCGATCACCGTATGGTGAACGATCCTAACCTGATCCTGGGG  1107
Query  241   CAGATAATTTTGCTGATTAAAGCGGCGATGATGGCACTGCTACCGCTCATCGCGGGCGTG  300
Sbjct  1108  CAGATAATTTTGCTGATTAAAGCGGCGATGATGGCACTGCTACCGCTCATCGCGGGCGTG  1167
Query  301   GTACTGGTGGCGCTTATCTCGCCGGTTATGCTTGGCGGCCTGATTTTTAGCGGTAAGTCG  360
Sbjct  1168  GTGCTGGTGGCGCTTATCTCGCCGGTTATGCTTGGCGGCCTGATTTTTAGCGGTAAGTCG  1227
Query  361   CTACAGCCAAAATTTTCTAAATTAAACCCGCTGCCGGGAATTAAGCGCATGTTTTCGGCG  420
Sbjct  1228  CTACAGCCAAAATTTTCTAAATTAAACCCGCTGCCGGGAATTAAGCGCATGTTTTCGGCG  1287
Query  421   CAGACCGGCGCGGAACTGCTAAAAGCGGTGTTGAAATCCACGCTGGTCGGCTGCGTTACC  480
Sbjct  1288  CAGACCGGCGCGGAATTGCTAAAAGCGGTGTTGAAATCCACGCTGGTCGGCTGCGTTACC  1347
Query  481   GGCTTTTATCTCTGGTATCACTGGCCACAAATGATGCGCCTGATGGCGGAGTCGCCGATC  540
```

```
Sbjct  1348      GGCTTTTATCTCTGGCATCACTGGCCGCAAATGATGCGCCTGATGGCGGAGTCGCCGATC  1407

Query  541       GTCGCAATGGGGAATGCGCTGGATCTGGTTGGACTCTGCGCGTTACTGGTGGTACTGGGC  600
Sbjct  1408      GTCGCAATGGGGAATGCGCTGGATCTGGTTGGACTCTGCGCGTTACTGGTGGTACTGGGC  1467

Query  601       GTGATTCCGATGGTGGGATTTGACGTGTTTTTCCAGATC  639
Sbjct  1468      GTGATTCCGATGGTGGGATTTGACGTGTTTTTCCAGATC  1506
```

Citrobacter koseri genome assembly PRJEB6512_assembly_1 ,scaffold CONTIG000001

Sequence ID: **emb|LK931336.1|** Length: 4763704 Number of Matches: 1

Range 1: 1092738 to 1093889

Score	Expect	Identities	Gaps	Strand	Frame
1131 bits(612)	0.0()	972/1152(84%)	0/1152(0%)	Plus/Plus	

Features:

```
Query  1         GTGGCAGAAGAGAGCGACGACGACAAAACAGAAGCCCCCACACCCCACCGACTTGAAAAA  60
Sbjct  1092738   GTGGCAGAAGAGAGCGACGACGACAAAACAGAAGCCCCCACACCCCACCGACTTGAAAAA  1092797

Query  61        GCGCGGGAAGAAGGGCAGATCCCCCGTTCCAGAGAACTGACCTCACTGCTGATATTGCTG  120
Sbjct  1092798   GCGCGGGAGGAAGGGCAAGTCCCCCGATCGAAGGAGTTGACCTCACTGCTGATTTTGCTG  1092857

Query  121       GTGGGCGTTTGTATTATTTGGTTCGGCGGCGAGTCGTTAGCGCGGCAACTGGCGGGAATG  180
Sbjct  1092858   GTGGGCGTTTGCATTATCTGGATCGGCGGTGAGTCGCTGGCGCGTCGCCTGGCGGGGATG  1092917

Query  181       CTCTCAGCAGGCCTGCACTTCGATCACCGTATGGTGAACGATCCTAACCTGATCCTGGGG  240
Sbjct  1092918   TTGTCGGCTGGGCTGCGTTTCGATCACCGCATGGTGAACGACCCCAATTTGATCCTCGGG  1092977

Query  241       CAGATAATTTTGCTGATTAAAGCGGCGATGATGGCACTGCTACCGCTCATCGCGGGCGTG  300
Sbjct  1092978   CAGATTATTTTGCTGATTAAAGAGGCGATGATTGCGTTGCTGCCGCTCATCACCGGCGTT  1093037

Query  301       GTACTGGTGGCGCTTATCTCGCCGGTTATGCTTGGCGGCCTGATTTTTAGCGGTAAGTCG  360
Sbjct  1093038   GTGCTGGTCGCGCTGATCTCGCCGGTCATGCTCGGCGGCCTGATTTTCAGCGGTAAATCA  1093097

Query  361       CTACAGCCAAAATTTTCTAAATTAAACCCGCTGCCGGGAATTAAGCGCATGTTTTCGGCG  420
Sbjct  1093098   CTGCAACCCAAATTTTCAAAACTGAATCCGCTATCGGGGATTAAACGCATGTTCTCCGCC  1093157

Query  421       CAGACCGGCGCGGAACTGCTAAAAGCGGTGTTGAAATCCACGCTGGTCGGCTGCGTTACC  480
Sbjct  1093158   CAGACCGGCGCGGAACTGCTGAAAGCAATCCTGAAATCGACGCTGGTGGGAAGCGTGGCG  1093217

Query  481       GGCTTTTATCTCTGGTATCACTGGCCACAAATGATGCGCCTGATGGCGGAGTCGCCGATC  540
Sbjct  1093218   GGATTTTATCTCTGGCATCACTGGCCGGAGATGATGCGCCTGATGGCGGAATCTCCGGTT  1093277

Query  541       GTCGCAATGGGGAATGCGCTGGATCTGGTTGGACTCTGCGCGTTACTGGTGGTACTGGGC  600
Sbjct  1093278   ACGGCGATGGGCAACGCGCTTGATTTAGTCGGGCTGTGCGCGCTTCTGGTTGTACTGGGC  1093337

Query  601       GTGATTCCGATGGTGGGATTTGACGTGTTTTTCCAGATCTTTAGCCACCTGAAAAAATTA  660
Sbjct  1093338   GTGATTCCGATGGTGGCGTTTGACGTCGTCTTCCAGATCTTCAGCCACCTGAAGAAACTG  1093397

Query  661       CGCATGTCGCGGCAGGACATTCGCGACGAATTTAAAGAGAGCGAAGGCGATCCGCATGTT  720
Sbjct  1093398   CGGATGTCGCGTAAAGATATTCGCGATGAATTTAAAGAGAGTGAAGGCGATCCGCACATC  1093457

Query  721       AAGGGCAAAATTCGCCAGATGCAACGCGCCGCCGCGCAGCGCCGCATGATGGAAGATGTG  780
Sbjct  1093458   AAAGGGAAAATTCGCCAGATGCAGCGTGCGGCTGCGCAGCGGCGCATGATGGAAGATGTG  1093517

Query  781       CCGAAAGCGGACGTCATTGTCACTAACCCGACGCACTATTCCGTGGCGCTGCAGTATGAC  840
Sbjct  1093518   CCCAAAGCGGATGTCATCGTGACTAACCCGACGCATTACTCGGTTGCGTTGCAGTACGAC  1093577

Query  841       GAAAACAAAATGAGCGCGCCGAAAGTGGTCGCGAAGGGGGCTGGATTAATAGCGCTGCGC  900
Sbjct  1093578   GAAAACAAAATGAGTGCGCCAAAAGTGGTCGCCAAAGGCGCGGGACTGGTGGCGCTGCGC  1093637

Query  901       ATTCGCGAGATCGGCGCTGAACATCGGGTTCCCACTTTAGAAGCGCCGCCGCTGGCGCGG  960
Sbjct  1093638   ATTCGCGAAATCGGCGCGGAACATCGGGTGCCGACCTTAGAAGCGCCCCCGCTGGCGCGC  1093697

Query  961       GCATTATATCGCCACGCCGAAATCGGTCAGCAAATTCCCGGGCAGTTATATGCTGCCGTT  1020
Sbjct  1093698   GCCTTGTATCGGCATGCTGAAATTGGTCAGCAAATCCCCGGCCAGCTCTATGCCGCAGTG  1093757

Query  1021      GCGGAAGTGTTGGCCTGGGTCTGGCAGCTTAAACGCTGGCGGCTTGCGGGCGGGCAACGT  1080
Sbjct  1093758   GCAGAAGTGCTGGCATGGGTATGGCAGCTCAAACGCTGGCGTCTTGCCGGTGGACAACGT  1093817

Query  1081      CCTCCACAACCTGAGAACCTTCCGGTGCCAGAAGCGCTGGATTTTATGAACGAGAAGAAT  1140
Sbjct  1093818   CCTCCACAACCTGAAAATCTTCCTGTGCCTGAAGCACTGGATTTTATGAACGAGAAGAAT  1093877

Query  1141      ACTGATGGCTAA  1152
Sbjct  1093878   ACTGATGGCTAA  1093889
```

Citrobacter koseri ATCC BAA-895, complete genome

Sequence ID: **gb|CP000822.1|** Length: 4720462 Number of Matches: 1
Range 1: 1056740 to 1057891

Score	Expect	Identities	Gaps	Strand	Frame
1125 bits(609)	0.0()	971/1152(84%)	0/1152(0%)	Plus/Plus	

Features:

```
Query  1        GTGGCAGAAGAGAGCGACGACGACAAAACAGAAGCCCCCACACCCCACCGACTTGAAAAA  60
Sbjct  1056740  GTGGCAGAAGAGAGCGACGACGACAAAACAGAAGCCCCCACACCCCACCGACTTGAAAAA  1056799
Query  61       GCGCGGGAAGAAGGGCAGATCCCCCGTTCCAGAGAACTGACCTCACTGCTGATATTGCTG  120
Sbjct  1056800  GCGCGGGAGGAAGGGCAAGTCCCCCGATCGAAGGAGTTGACCTCACTGCTGATTTTGCTG  1056859
Query  121      GTGGGCGTTTGTATTATTTGGTTCGGCGGCGAGTCGTTAGCGCGGCAACTGGCGGGAATG  180
Sbjct  1056860  GTGGGCGTTTGCATTATCTGGATCGGCGGTGAGTCGCTGGCGCGTCGCCTGGCGGGGATG  1056919
Query  181      CTCTCAGCAGGCCTGCACTTCGATCACCGTATGGTGAACGATCCTAACCTGATCCTGGGG  240
Sbjct  1056920  TTGTCGGCTGGGCTGCGTTTCGATCACAGCATGGTGAACGACCCCAATTTGATCCTCGGG  1056979
Query  241      CAGATAATTTTGCTGATTAAAGCGGCGATGATGGCACTGCTACCGCTCATCGCGGGCGTG  300
Sbjct  1056980  CAGATTATTTTGCTGATTAAAGAGGCGATGATTGCGTTGCTGCCGCTCATCACCGGCGTT  1057039
Query  301      GTACTGGTGGCGCTTATCTCGCCGGTTATGCTTGGCGGCCTGATTTTTAGCGGTAAGTCG  360
Sbjct  1057040  GTGCTGGTCGCGCTGATCTCGCCGGTCATGCTCGGCGGCCTGATTTTCAGCGGTAAATCA  1057099
Query  361      CTACAGCCAAAATTTTCTAAATTAAACCCGCTGCCGGGAATTAAGCGCATGTTTTCGGCG  420
Sbjct  1057100  CTGCAACCCAAATTTTCAAAACTGAATCCGCTATCGGGGATTAAACGCATGTTCTCCGCC  1057159
Query  421      CAGACCGGCGCGGAACTGCTAAAAGCGGTGTTGAAATCCACGCTGGTCGGCTGCGTTACC  480
Sbjct  1057160  CAGACCGGCGCGGAACTGCTGAAAGCAATCCTGAAATCGACGCTGGTGGGAAGCGTGGCT  1057219
Query  481      GGCTTTTATCTCTGGTATCACTGGCCACAAATGATGCGCCTGATGGCGGAGTCGCCGATC  540
Sbjct  1057220  GGATTTTATCTCTGGCATCACTGGCCGGAGATGATGCGCCTGATGGCGGAATCTCCGGTT  1057279
Query  541      GTCGCAATGGGGAATGCGCTGGATCTGGTTGGACTCTGCGCGTTACTGGTGGTACTGGGC  600
Sbjct  1057280  ACGGCGATGGGCAACGCGCTTGATTTAGTCGGGCTGTGCGCGCTTCTGGTTGTACTGGGC  1057339
Query  601      GTGATTCCGATGGTGGGATTTGACGTGTTTTTCCAGATCTTTAGCCACCTGAAAAAATTA  660
Sbjct  1057340  GTGATTCCGATGGTGGCGTTTGACGTCGTCTTCCAGATCTTCAGCCACCTGAAGAAACTG  1057399
Query  661      CGCATGTCGCGGCAGGACATTCGCGACGAATTTAAAGAGAGCGAAGGCGATCCGCATGTT  720
Sbjct  1057400  CGGATGTCGCGTAAAGATATTCGCGATGAATTTAAAGAGAGTGAAGGCGATCCGCACATC  1057459
Query  721      AAGGGCAAAATTCGCCAGATGCAACGCGCCGCCGCGCAGCGCCGCATGATGGAAGATGTG  780
Sbjct  1057460  AAAGGGAAAATTCGCCAGATGCAGCGTGCGGCTGCGCAGCGGCGCATGATGGAAGATGTG  1057519
Query  781      CCGAAAGCGGACGTCATTGTCACTAACCCGACGCACTATTCCGTGGCGCTGCAGTATGAC  840
Sbjct  1057520  CCCAAAGCGGATGTTATCGTGACTAACCCGACGCATTACTCGGTTGCGTTGCAGTACGAC  1057579
Query  841      GAAAACAAAATGAGCGCGCCGAAAGTGGTCGCGAAGGGGGCTGGATTAATAGCGCTGCGC  900
Sbjct  1057580  GAAAACAAAATGAGTGCGCCAAAAGTGGTCGCCAAAGGCGCGGGACTGGTGGCGCTGCGC  1057639
Query  901      ATTCGCGAGATCGGCGCTGAACATCGGGTTCCCACTTTAGAAGCGCCGCCGCTGGCGCGG  960
Sbjct  1057640  ATTCGCGAAATCGGCGCGGAACATCGGGTGCCGACCTTAGAAGCGCCCCCGCTGGCGCGC  1057699
Query  961      GCATTATATCGCCACGCCGAAATCGGTCAGCAAATTCCCGGGCAGTTATATGCTGCCGTT  1020
Sbjct  1057700  GCCTTGTATCGGCATGCTGAAATTGGTCAGCAAATCCCCGGCCAGCTCTATGCCGCAGTG  1057759
Query  1021     GCGGAAGTGTTGGCCTGGGTCTGGCAGCTTAAACGCTGGCGGCTTGCGGGCGGGCAACGT  1080
Sbjct  1057760  GCAGAAGTGCTGGCATGGGTATGGCAGCTCAAACGCTGGCGTCTTGCCGGTGGGCAACGT  1057819
Query  1081     CCTCCACAACCTGAGAACCTTCCGGTGCCAGAAGCGCTGGATTTTATGAACGAGAAGAAT  1140
Sbjct  1057820  CCTCCACAACCTGAAAATCTTCCTGTGCCTGAAGCACTGGATTTTATGAACGAGAAGAAT  1057879
Query  1141     ACTGATGGCTAA  1152
Sbjct  1057880  ACTGATGGCTAA  1057891
```

Citrobacter rodentium ICC168, complete genome
Sequence ID: **emb|FN543502.1|** Length: 5346659 Number of Matches: 1
Range 1: 2043223 to 2044358

Score	Expect	Identities	Gaps	Strand	Frame
1040 bits(563)	0.0()	953/1144(83%)	16/1144(1%)	Plus/Minus	

Features:
flagellar biosynthesis protein FlhAflagellar biosynthesis protein FlhB

```
Query  17       ACGACGACAAAACAGAAGCCCCCACACCCCACCGACTTGAAAAAGCGCGGGAAGAAGGGC  76
Sbjct  2044358  ACGACGACAAAACAGAAGCCCCCACACCCCACCGACTTGAAAAAGCGCGGGAAGAAGGGC  2044299
```

```
Query  77       AGATCCCCCGTTCCAGAGAACTGACCTCACTGCTGATATTGCTGGTGGGCGTTTGTATTA  136
Sbjct  2044298  AGATCCCCCGTTCCAGAGAGCTGACCTCGCTGCTGATCTTGCTGGTGGGCGTTTGTATTA  2044239
Query  137      TTTGGTTCGGCGGCGAGTCGTTAGC--G-CGGCAA-CTGGCGGGAATGCTCTCAGCAGGC  192
Sbjct  2044238  TCTGGATTGGCGGCGAGTCGCTGGCCAGACGGCTCTCTG-CG---ATGCTGTCGGCAGGG  2044183
Query  193      CTGCACTTCGATCACCGTATGGTGAACGATCCTAACCTGATCCTGGGGCAGATAATTTTG  252
Sbjct  2044182  CTGCGCTTTGACCACAGTATGGTCAACGATCCCAGTCTGATCCTCGGGCAAATCATTCTG  2044123
Query  253      CTGATTAAAGCGGCGATGATGGCACTGCTACCGCTCATCGCGGGCGTGGTACTGGTGGCG  312
Sbjct  2044122  CTGATAAAAGAAGCGATGATCGCGCTGCTGCCGCTGATTACCGGCGTGGTGCTGGTGGCG  2044063
Query  313      CTTATCTCGCCGGTTATGCTTGGCGGCCTGATTTTTAGCGGTAAGTCGCTACAGCCAAAA  372
Sbjct  2044062  CTGATCTCGCCGGTGCTGCTCGGCGGGCTGATCTTTAGCGGTAAATCGCTGCAACCGAAA  2044003
Query  373      TTTTCTAAATTAAACCCGCTGCCGGGAATTAAGCGCATGTTTTCGGCGCAGACCGGCGCG  432
Sbjct  2044002  TTTTCTAAACTCAATCCGCTGCCCGGCATCAAGCGTATGTTCTCCGCGCAGTCCGGGGCG  2043943
Query  433      GAACTGCTAAAAGCGGTGTTGAAATCCACGCTGGTCGGCTGCGTTACCG-GCTTTTATCT  491
Sbjct  2043942  GAGTTGCTGAAAGCGGTGCTGAAATCGACGCTGGTCGGCGGCGTGACGGCGCTG-TATCT  2043884
Query  492      CTGGTATCACTGGCCACAAATGATGCGCCTGATGGCGGAGTCGCCGATCGTCGCAATGGG  551
Sbjct  2043883  GTGGCATAAATGGCCGCAGATGATGCGTCTGATGGCGGAATCGCCCATCACGGCGATGGG  2043824
Query  552      GAATGCGCTGGATCTGGTTGGACTCTGCGCGTTACTGGTGGTACTGGGCGTGATTCCGAT  611
Sbjct  2043823  CGACGCGCTGGATCTCGTCGGGCTGTGCGCGCTGCTGGTGGTGCTGGGCGTTATCCCGAT  2043764
Query  612      GGTGGGATTTGACGTGTTTTTCCAGATCTTTAGCCACCTGAAAAAATTACGCATGTCGCG  671
Sbjct  2043763  GGTCGGCTTTGACGTTATCTTCCAGATCTTCAGCCACCTGAAAAAATTACGCATGTCGCG  2043704
Query  672      GCAGGACATTCGCGACGAATTTAAAGAGAGCGAAGGCGATCCGCATGTTAA-GGGCAAAA  730
Sbjct  2043703  TCAGGATATCCGCGATGAATTCAAACAGAGCGAGGGCGACCCGCATGTGAAAGGGCGC-A  2043645
Query  731      TTCGCCAGATGCAACGCGCCGCCGCGCAGCGCCGCATGATGGAAGATGTGCCGAAAGCGG  790
Sbjct  2043644  TCCGCCAGATGCAGCGGGCGGCGGCGCGCCAGCGCATGATGAGCGACGTGCCCAACGCTG  2043585
Query  791      ACGTCATTGTCACTAACCCGACGCACTATTCCGTGGCGCTGCAGTATGACGAAAACAAAA  850
Sbjct  2043584  ACGTGATTGTCACTAACCCTACCCACTACTCGGTGGCGTTACAGTATGACGAAAACAAAA  2043525
Query  851      TGAGCGCGCCGAAAGTGGTCGCGAAGGGGGCTGGATTAATAGCGCTGCGCATTCGCGAGA  910
Sbjct  2043524  TGAGCGCGCCAAAAGTGGTCGCCAAAGGGGCGGGACTGGTGGCGCTGCGCATTCGCGAGA  2043465
Query  911      TCGGCGCTGAACATCGGGTTCCCACTTTAGAAGCGCCGCCGCTGGCGCG-G-GCATTATA  968
Sbjct  2043464  TTGGCGCGGAAAACAAAATTCCCACTCTGGAAGCGCCCGCCGCTGGCGCGCGCGC-TT-TA  2043407
Query  969      TCGCCACGCCGAAATCGGTCAGCAAATTCCCGGGCAGTTATATGCTGCCGTTGCGGAAGT  1028
Sbjct  2043406  TCGCCATGCTGAAATTGGTCAGCAAATTCCCGGGCAGCTGTACGCCGCGGTGGCGGAGGT  2043347
Query  1029     GTTGGCCTGGGTCTGGCAGCTTAAACGCTGGCGGCTTGCGGGCGGGCAACGTCCTCCACA  1088
Sbjct  2043346  CCTGGCCTGGGTGTTGCAGCTTAAGCGCTGGCGGCTTGCCGGCGGGGAACGCCCACCACA  2043287
Query  1089     ACCTGAGAACCTTCCGGTGCCAGAAGCGCTGGATTTTATGAACGAGAAGAATACTGATGG  1148
Sbjct  2043286  ACCTGCAAATCTTCCGGTGCCTGAAGCACTGGATTTTATGAACGAGAAGACTACCGATGG  2043227
Query  1149     CTAA  1152
Sbjct  2043226  CTAA  2043223
```

Supplementary FIGURE 1 | BLAST search results using *Salmonella* Typhimurium *flhB* nucleotide sequence (GenBank accession no. NC_003197.1 segment 2010283-2011434) against the nucleotide collection (nr/nt) database. The maximum number of aligned sequences to display was set to the maximum value of 20,000, and the other parameters were set to default values. The results showed that *flhB* gene of *S.* Pullorum/Gallinarum covered 83% of that of other serovars in length (red box), indicating a deficient *flhB* exists only in *S.* Pullorum/Gallinarum.
